# Spatial Patterns in Water Quality Changes during Dredging in Tropical Environments

**DOI:** 10.1371/journal.pone.0143309

**Published:** 2015-12-02

**Authors:** Rebecca Fisher, Clair Stark, Peter Ridd, Ross Jones

**Affiliations:** 1 Australian Institute of Marine Science, Perth, Western Australia, Australia; 2 Western Australian Marine Science Institution, Perth, Western Australia, Australia; 3 Oceans Institute, University of Western Australia, Perth, Western Australia, Australia; 4 School of Engineering and Physical Sciences, James Cook University, Townsville, Queensland, Australia; Università della Calabria, ITALY

## Abstract

Dredging poses a potential risk to tropical ecosystems, especially in turbidity-sensitive environments such as coral reefs, filter feeding communities and seagrasses. There is little detailed observational time-series data on the spatial effects of dredging on turbidity and light and defining likely footprints is a fundamental task for impact prediction, the EIA process, and for designing monitoring projects when dredging is underway. It is also important for public perception of risks associated with dredging. Using an extensive collection of *in situ* water quality data (73 sites) from three recent large scale capital dredging programs in Australia, and which included extensive pre-dredging baseline data, we describe relationships with distance from dredging for a range of water quality metrics. Using a criterion to define a zone of potential impact of where the water quality value exceeds the 80^th^ percentile of the baseline value for turbidity-based metrics or the 20^th^ percentile for the light based metrics, effects were observed predominantly up to three km from dredging, but in one instance up to nearly 20 km. This upper (~20 km) limit was unusual and caused by a local oceanographic feature of consistent unidirectional flow during the project. Water quality loggers were located along the principal axis of this flow (from 200 m to 30 km) and provided the opportunity to develop a matrix of exposure based on running means calculated across multiple time periods (from hours to one month) and distance from the dredging, and summarized across a broad range of percentile values. This information can be used to more formally develop water quality thresholds for benthic organisms, such as corals, filter-feeders (e.g. sponges) and seagrasses in future laboratory- and field-based studies using environmentally realistic and relevant exposure scenarios, that may be used to further refine distance based analyses of impact, potentially further reducing the size of the dredging footprint.

## Introduction

Dredging and dredge material (spoil) disposal releases sediments into the water column, creating turbid plumes that can drift onto nearby marine habitats [1]. The elevated suspended sediment concentrations and the eventual settlement of the sediments can have a range of negative effects on benthic filter and suspension feeding organisms [1–7]. By altering the characteristics of underwater light, the increased turbidity can also have marked effects on primary producers. This is of particular significance for habitat-forming groups such as corals and seagrasses, as their loss would also result in loss of the habitat-associated biodiversity [8,9]. There are many examples of dredging programs that have had widespread environmental effects on these communities [10–13] and dredging programs usually require active management when underway to minimize environmental harm [14–18].

Despite the well-known effects of dredging there have been surprisingly few peer reviewed studies of water quality conditions associated with dredging in tropical environments. Published studies include [19–21] and a number of publically available technical reports and higher level summaries of individual projects [15,22,23]. Suspended sediment concentrations in the hoppers of trailing suction hopper dredges (TSHDs, considered the workhorse of the dredging fleet (see [24])), can reach tens of grams L^-1^, but typically undergo an initial rapid 10–100 fold dilution when overflowing to the receiving water [25–29]. Suspended sediments in the associated plumes decrease with both time [28,30,31] and distance from dredging, as lateral dispersion, mixing with ambient water and settling at the seabed occurs (see for example [26,29,32]).

The lateral movement of dredging plumes, and diminution in space and time, is especially important for impact prediction purposes and the Environmental Impact Assessment (EIA) process. Environmental policy for dredging, in Australia at least, is based on this principle, with dredging proponents required to manage projects according to a spatially-based zonation scheme identifying areas which could be exposed to plumes (referred to as a ‘zone of influence’) and where effects (i.e. mortality) of underlying communities could occur [17,33]. Although highly site and project specific, some dredging plumes can travel up to 70 km [34] and a basic task is to quantify the intensity, frequency and duration of pressure fields (see [35] for a definition of the term pressure field) with respect to distance from the dredging activities and ultimately understand any possible effects on the local ecology.

In addition to the EIA process, establishing an evidence-based footprint of the scale of potential impacts is becoming increasingly important for public perception [29]. Effects on water quality associated with the operations of TSHDs in the UK marine aggregate industry has recently been reviewed, and effects typically occurred from a few hundred metres to up three km from the point of dredging [29]. This three km limit is useful as a broad limit of potential impact, but TSHDs in the aggregate industry are generally smaller than those used in maintenance and capital dredging for channel widening and deepening, and tend to produce less fines because of the coarser nature of the material being dredged.

Recently several large water quality data sets have become available from a sequence of major capital dredging campaigns in the Pilbara region of tropical Western Australia (WA [18]). Three of the larger projects involved dredging and subsequent marine disposal of ~34 Mm^3^ or ~60 Mt tonnes of sediment (using a conversion factor of 1.7 g/cm^3^ see [36]). For comparative purposes the UK marine aggregate industry extracts on average 20 Mt tonnes of sediment annually. The Australian state and federal regulatory conditions for the Australian projects (see Ministerial approval statements MS757, MS800, MS840 searchable on the WA EPA website) required detailed baseline, surveillance and compliance water quality and biological monitoring programs (for a discussion of these terms see [35]). The water quality monitoring included measurements of turbidity and light levels on sub-hourly time scales at multiple reference and potential impact sites. Measurements were also made at different distances from the dredging, over extended periods (>1 year) and in many cases included extended pre-dredging baseline periods. This has provided water quality data where the detailed effects of dredging can be assessed with respect to distance from the turbidity-generating events as well as allowing the changes to be placed within the context of natural background turbidity events associated with wind and waves [37–40].

Analyses of the temporal characteristics of the water quality from the Pilbara datasets close to the dredging have already highlighted the variable nature of the plumes with fluctuations of turbidity of 2–3 orders of magnitude over the course of a day [21]. Dredging was found to change the overall probability distribution of turbidity values, increasing the frequency of extreme values and altering the intensity, duration and frequency of the turbidity events over background levels. There were marked changes in photosynthetically active radiation (PAR) in the shallow reef environment associated with the turbidity, including frequent daytime ‘twilight’ periods and occasionally periods of complete darkness. However, a more common feature was extended periods (i.e. days to weeks) of low light. The choice of summary statistics used (mean versus median etc), as well as the temporal scale examined (hours, days, weeks etc) was found to be very important for interpreting the data. Upper percentile values (e.g. 99^th^, 95^th^) of water quality parameters were highly elevated over short periods, but converged to values only slightly above baseline states over longer periods (weeks to months).

In this study we further analyse the Pilbara datasets using similar statistical summary techniques, but this time examine the spatial characteristics of the data. The information has provided a first order approximation of the distance where any dredging related effects become indistinguishable from natural variation. The information has also provided a matrix of data that can be used to design future manipulative experiments on the effects of dredging pressure on tropical marine organisms using environmentally realistic/relevant exposure scenarios.

## Methods

### Study sites

**Fig 1:**
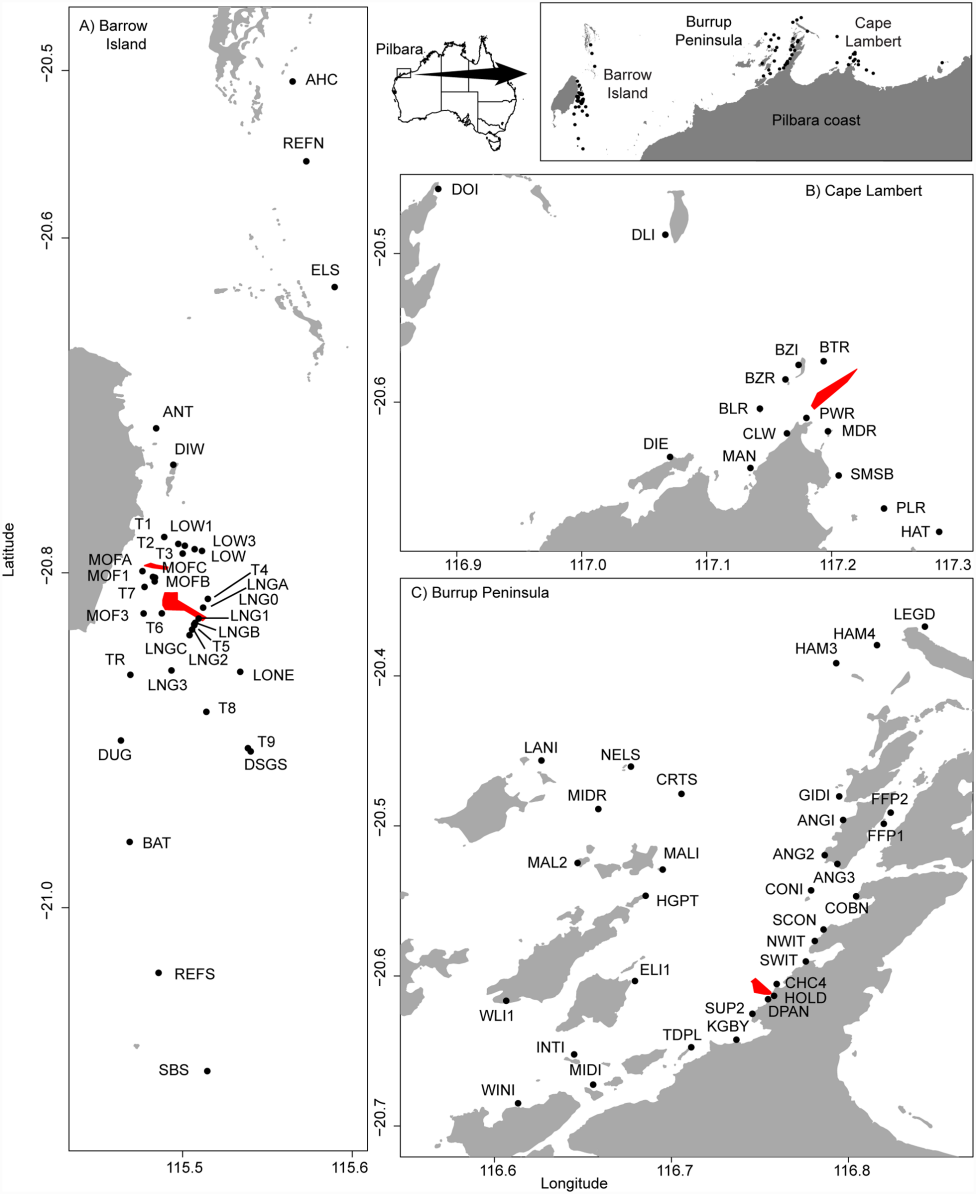
Water quality monitoring sites for three capital dredging projects in the Pilbara region (Western Australia). Shown are sites for the Barrow Island, Burrup Peninsula and Cape Lambert dredging projects (see Ministerial approval statements MS757, MS800, MS840 searchable on the WA EPA website). Polygons in red show the primary location(s) of dredging activity, including: the materials offloading facility (MOF) and the LNG jetty access channel and turning basin for (A) the Barrow Island project (B) the Cape Lambert project and (C) the Burrup Peninsula project. Maps were constructed in R using the package rgdal [41] based on the GA 2004 coastline dataset [42], and arranged using gridBase [43], with additional edits carried out using Adobe Illustrator [44].

All necessary permits for deploying the instrumentation were sought from the relevant state authority, the Western Australia Department of Environment and Conservation. The field studies did not involve endangered or protected species. Water quality data was collected at 32 sites for the Burrup Peninsula Project, 26 sites during the Barrow Island project, and 15 sites during the Cape Lambert project (Fig 1). Full details for each site sampled, including total baseline and dredge period sampling days, water depth (where available) and distances of the monitoring sites from the main dredging activities are listed in S1 Table and [21]. All three projects had sites spanning distances of up to ~30 km from the location of dredging activities.

While the three projects were relatively near to each other, spanning a total distance of <250 km, they did occur in slightly different marine settings and therefore represent a range of coral reef environments; with Barrow Island representing an offshore ‘clear water’ environment, Cape Lambert an exposed nearshore cape or headland, and Burrup Peninsula an enclosed inshore turbid reef environment. Sediment characteristics varied somewhat among the three projects, although all three were generally characterised by unconsolidated carbonate sediments, ranging in grain size from gravel to fine silts [21]. Both the Cape Lambert and Burrup Peninsula projects tended to have finer sediments grain sizes at inshore sites closer to the dredging areas.

All water quality data was processed similarly to ensure data integrity and to remove potentially erroneous values. Full details of the data processing and cleaning steps can be found in [21]. Briefly, all turbidity data was aggregated for all sites and retained at the finest temporal resolution (10 or 30 min, depending on the logger type and project) or aggregated to a daily mean or percentile value as required for the various analyses. Light data at the finest temporal resolution were fitted using a Generalised Additive Model (GAM) for each day separately using the mgcv package [45] in R [46]. Days for which insufficient light data were available throughout the full light cycle were removed and not included in the analysis. Each fitted daily model was then used to estimate photosynthetically active radiation (400–750 nm, PAR) values for every second throughout the daylight period, based on monthly sunset and sunrise times. The sum of the per second quantum flux measurements were then added together to calculate the daily light integral (DLI) as mol photons m^-2^.

Turbidity (NTU) and light (PAR as DLI) data were summarised using a range of methods that represent different water quality hazard metrics that might have a negative impact on surrounding benthic communities such as coral reefs. For all three dredging projects we examined a range of turbidity metrics, including: mean, median, 80^th^ percentile, 95^th^ percentile and maximum daily turbidity values, and running 7 and 14 day mean, median and 80^th^ percentile turbidity values. Running mean and percentile values were calculated using the runmean and runquantile functions from the caTools package in R [47]. In addition, several light based metrics were examined for the Barrow Island project where sufficient light data were available across both dredging and baseline periods, including: mean DLI, 7 and 14 day running mean DLI; the mean portion of the day <5, 10, 15 and 20 μmol photons m^-2^s^-1^ (equivalent to 0.2, 0.4, 0.6 and 0.8 mol photons m^-2^ assuming 12 h per day of light at those irradiance levels); and the total number of days (per year) < 1, 12 and 46 μmol photons m^-2^s^-1^ (equivalent to ~ 0, 0.5 and 2 DLI). Running means (or percentiles) of the 10 or 30 min turbidity data were calculated using the 7 and 14 day running time periods by converting the data series for each site into an S3 time series object using the zoo function from the zoo library [48] then applying the runmean or runquantile function from the caTools library [47].

### Spatial patterns in turbidity & light

A generalised additive mixed modelling (GAMM [49]) approach was used to examine spatial variation and the effect of distance from dredging on the calculated turbidity and light summary metrics. Distances from dredge activities were calculated using the ArcGis 10.2 Esri software [50], and represent the nearest distance to shape file features representing the channels for the Burrup Peninsula and the Cape Lambert projects, and MOF and LNG footprints for the Barrow Island project (Fig 1). At Barrow Island, four sites (DSGS, LONE, T8 and T9) were near the spoil disposal area (<3 km) and were not included in statistical analyses of distance from dredging. For simplicity, analyses were carried out for all three dredging projects separately, as preliminary examination indicated there would be slightly different directional effects among the three projects which would lead to high order interactions if they were analysed together. Broad-scale distance decay relationships were initially examined for all three studies across a range of time scales (hourly, daily, and fortnightly running means for NTU; daily weekly and fortnightly running means for DLI) based on either the 80^th^ (for NTU) or 20^th^ (for DLI) percentile values for each site during the pre-dredging and dredging phases. Formal statistical analyses were undertaken at a finer temporal scale to more closely examine spatial patterns in distance decay relationships. For the detailed distance analysis, data were summarized as 95^th^ percentiles, quarterly for each year of data from both the pre-dredging and dredging phases for each site. Quarterly summaries were used in these analyses because they allowed a reasonable level of temporal variation to be included whilst avoiding issues associated with serial autocorrelation inherent in shorter summaries [51]. While there are time series analysis methods available to account for such autocorrelation in models [52], an analysis at the daily level was also prohibited by the large amount of data and available computing power. Quarterly summaries were based on a 95^th^ percentile (i.e. the near worst case scenario during that quarter at that site). This upper percentile value was used in preference to a median, as this better identifies times and sites when and where high turbidity events occurred, as median values can miss important turbidity events associated with dredging (see [21]). Quarterly summaries were only used where at least three weeks of valid data were available.

For all turbidity based metrics, only distance from dredging was included as a continuous variable in the models. For the light based metrics depth was included as an additional continuous variable to account for the effects of attenuation through the water column [53,54]. Where depth was included in the best model (see below), the relationships with distance were plotted after effectively removing any depth effects. Continuous variables (distance and depth) were fitted as smoothers using cubic regression splines via the gamm4 function in the package gamm4 [49]. To ensure monotonic relationships with distance and depth (when included), and to more generally avoid over-fitting smoothers, the k parameter (basis dimension, see [45]) in the smoother argument was set at 4. Both site and yearly quarter were included as independent random effects.

The factors considered included a treatment effect (during baseline/during dredging) and two spatial directional variables (N/S and E/W) representing either North or South, or East versus West of the primary dredging activity. Two-way interactions between each of the directional variables and the baseline versus dredging treatment variables were also included, such that an effect during dredging for only one direction could be accommodated. The factor variables were included as an offset term (moving the overall relationship up or down), or as a ‘by’ argument to the gam smooths (see [45]), representing an interaction between the distance from dredge effect and each factor (a different smooth is fitted for each level of the factor). The full (most complex model fitted) included the three way interaction between distance, dredging treatment and either one of the directional variables, and was thus:

R~s(Dep) + s(Di)*Dr*NS + Dr*NS + Dr + NS + (1|Site) + (1|quarter)

Where: *R* represents the particular response metric being examined (light or turbidity based); s(Dep) represents the smoothing function applied to depth (only included for light based metrics); s(Di) represents the smoothing function applied to distance from dredging; NS represents the fixed factor delineating North versus South of the dredging location (inter-changeable with EW, which delineates East from West of the dredging location); (1|Site) signifies inclusion of a random site effect; and (1|quarter) signifies inclusion of a random quarterly effect. Due to limited baseline data at some sites for the Burrup Peninsula project, the full three way interaction was not included, with all baseline combined and only the during dredging data delineated into spatial levels.

A full subsets analysis approach was used, where all possible models are compared (including an intercept only ‘null’ model) using the model selection statistic AICc [55], with the model having fewest parameters within 2 AICc units of that with the lowest AICc value selected as the ‘best’ or most parsimonious model [56].

Once the optimal model structure was determined using GAMM the equivalent parametric power decay model was fitted using the nls function from the stats package in R [46,57,58]. For those models where there was evidence of an effect of distance from the dredge site, the distance at which the fitted curve (which essentially represents the median value) falls below the 80^th^ percentile of the baseline value was calculated. This test of distance of effect is effectively the *P*_50_–*P*_80_ approach of the ANZECC/ARMCANZ guidelines [59] which is used to define water quality changes that may result in a ‘measurable perturbation’ [59]. These distances of effect were calculated separately for each level of any factors identified as important in the most likely model (e.g. north versus south), and used to compare the relative distances at which the effects of dredging are observed.

### Detailed plume analysis at Barrow Island

The dredge plume at Barrow Island was unusual in that it moved predominantly in a southward direction [60]. With a large number of water quality monitoring sites regularly spaced along the principal axis of flow, and at increasing distance from a fairly focal point of dredging, this provided an ideal opportunity to examine the spatial structure of dredge plumes in much finer detail. High resolution satellite imagery during the baseline period (23^rd^ November 2008) and during dredging (24 July 2010 & 29 August 2010) were also analysed to examine the relationship between visually apparent plumes and real time water quality. Images from were sourced either from the Japan Aerospace Exploration Agency (JAXA) Advanced Land Observing Satellite (ALOS) Advanced Visible and Near Infrared Radiometer (AVNIR-2) and the Landsat 5 Thermal Mapper. In addition, we examined the cumulative probability function of turbidity (NTU) and light (DLI) and how these change with distance from dredging using the series of sites south of the primary dredging activity at Barrow Island.

## Results

### Broad-scale patterns of distance decay

**Fig 2:**
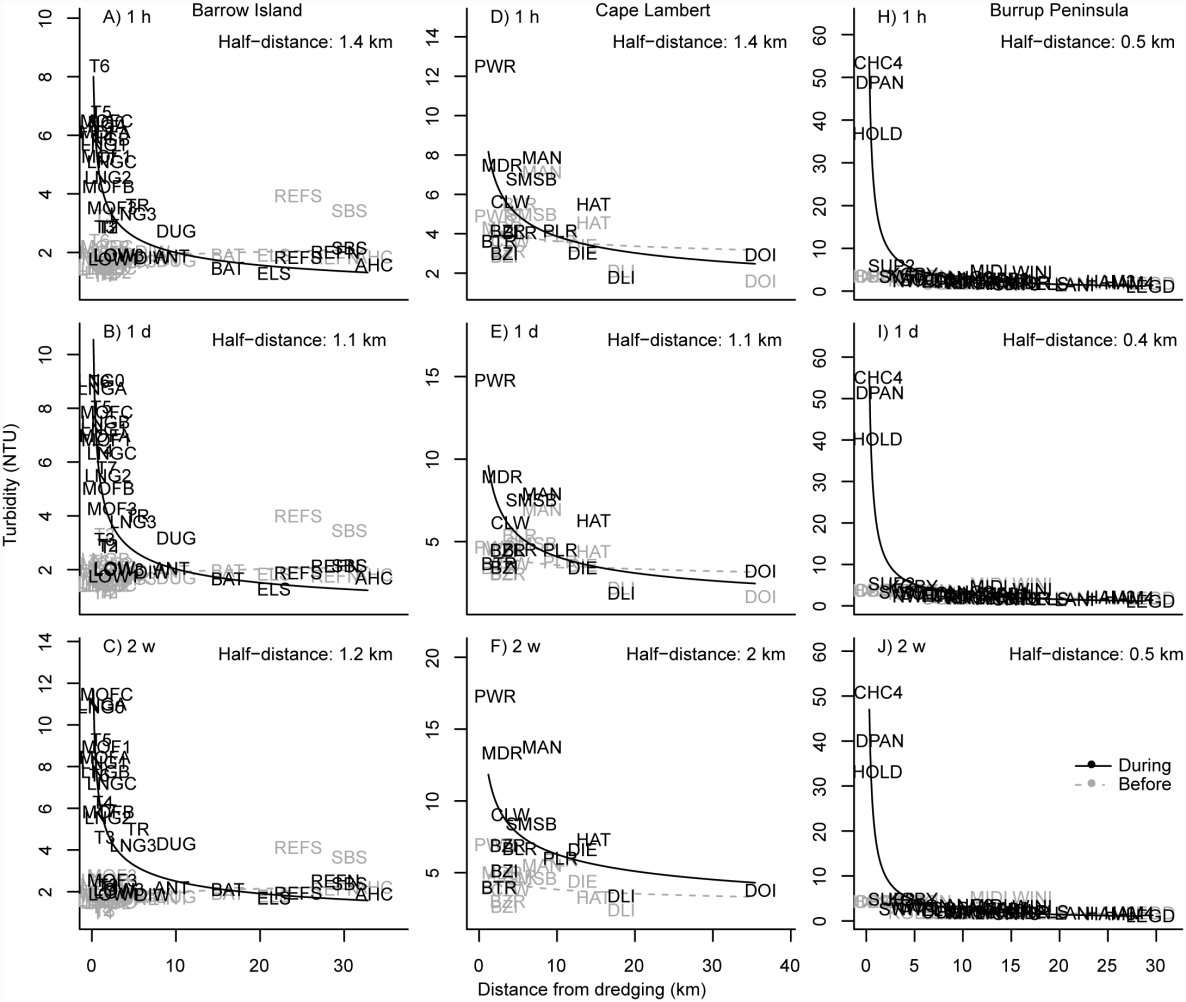
Distance decay relationships based on turbidity (80^th^ percentile NTU) across the three different dredging programs (Barrow Island, Cape Lambert and Burrup Peninsula). Shown are decay relationships based on the 80^th^ percentile value for each site for the hourly (panels A, D, H), daily (panels B, E, I) and fortnightly (panels C, F, J) running means. Half distance values represent that distance at which each turbidity metric decays to half of the predicted value at 200 m from the dredging activity.

**Fig 3:**
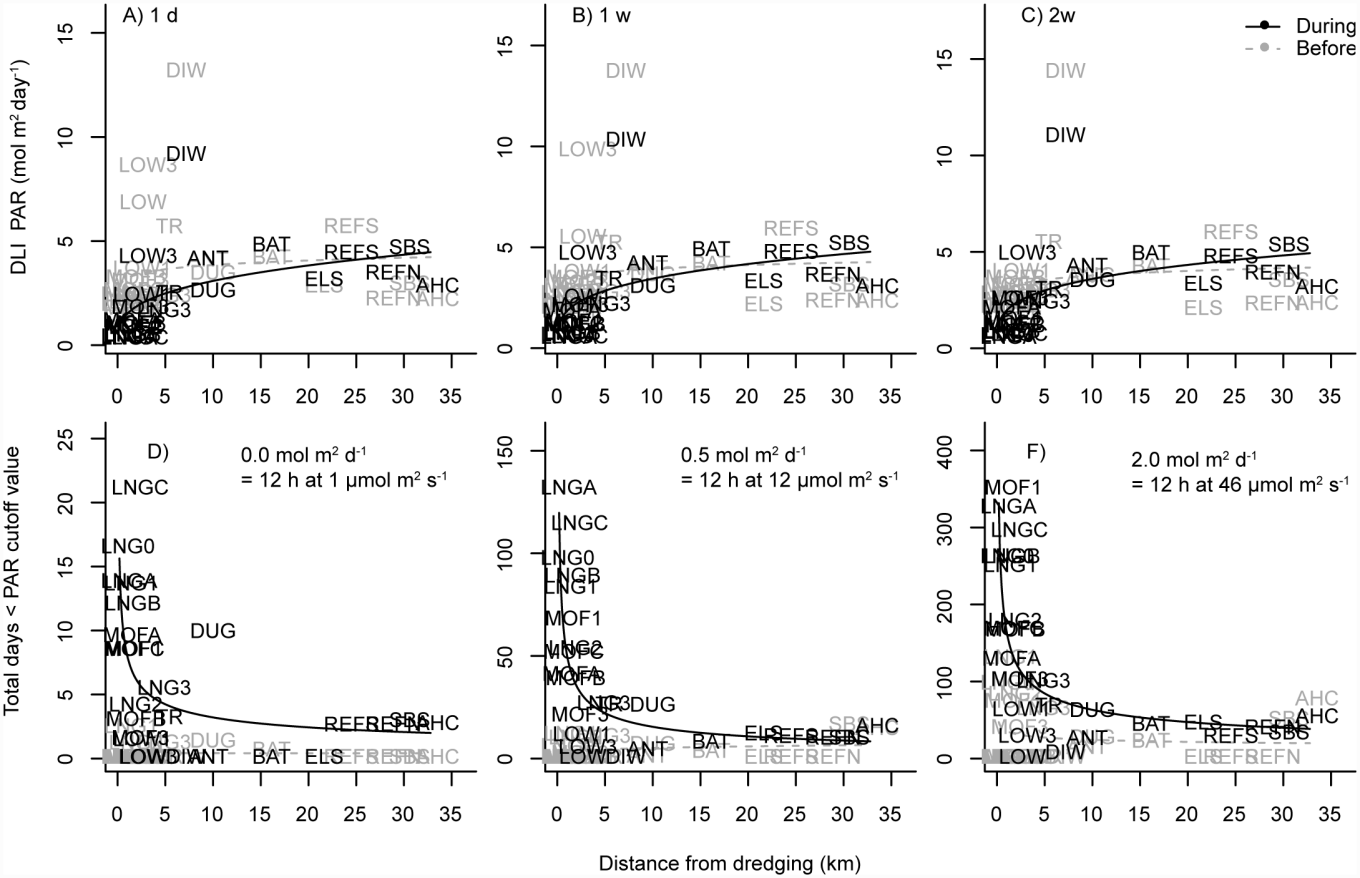
Distance decay relationships based on light for the Barrow Island dredging program. Shown are distance relationships based on the 20^th^ percentile of the daily light integral (DLI) value for 1 day (A), 1 week (B) and 2 week running means (C); and the total number of days in near-darkness (normalised to 1 year) for DLI threshold values of ~0 mol m^-2^ photons (D), 0.5 mol m^-2^ photons (E) and 2.0 mol m^-2^ photons (F).

Scatterplots of a range of turbidity (all projects) and light based metrics (Barrow Island only) of water quality clearly indicated a strong power-decay effect with distance from dredging for all three projects, which was absent during the baseline phase (Figs 2 & 3). Distances from dredging effects were apparent in the 80^th^ percentile values observed across sites during the dredging phase for a range of temporal scales, from 1 h to 2 week running mean turbidity values, with no such spatial patterns apparent prior to dredging (Fig 2). The 80^th^ percentiles values for turbidity decayed rapidly with increasing distance from dredging across all studies, with half-distance values (the distances at which turbidity values fell to half of those observed at 200 m of dredging) from just over 1 km for the Barrow Island project (Fig 2A, 2B and 2C), 400 m for Burrup Peninsula project (Fig 2D and 2E) and up to 2 km for the Cape Lambert project (Fig 2H, 2I and 2J). Similar relationships with distance from dredging were also observed for light related water quality metrics at Barrow Island, with DLI values increasing rapidly with increasing distance (Fig 3A, 3B and 3C), and the number of observed days at various darkness-cut off levels declining rapidly with distance (Fig 3D, 3E and 3F). Near dredging some sites can experience over 20 days per year where the DLI is near 0 mol photos m^-2^, over 120 days per year where DLI values are less than 0.5 mol photos m^-2^ and upwards of 340 days per year where DLI levels are less than 2 mol photos m^-2^ (Fig 3).

**Fig 4:**
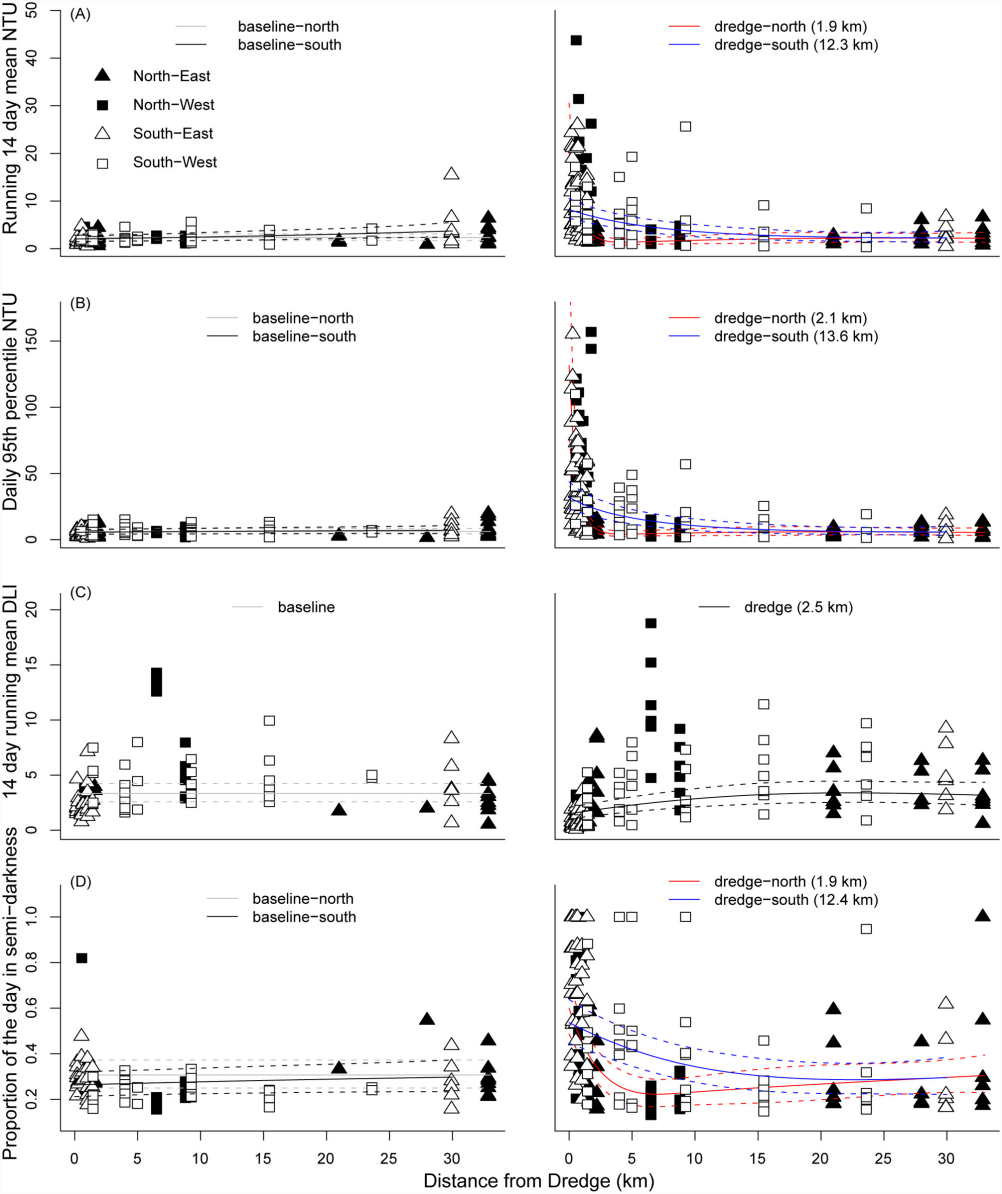
Distance decay relationships for four representative water quality metrics during the Barrow Island project. Shown are: (A) Daily 95^th^ percentile of turbidity, (B) running 14 day mean turbidity, (C) running 14 day mean DLI and (D) proportion of the day below 0.2 DLI. Fitted curves represent fitted best fit Generalised Additive Mixed Models ± 95% confidence. Baseline and dredge periods were fitted as a two way interaction with distance from dredge, or as a three way interaction as appropriate (North/South or East/West of the location of the primary dredging activity, see methods for further details). Values in parentheses indicate the distance at which the fitted curve falls below the 80^th^ percentile of the baseline value (i.e. the dredging effect becomes negligible). Data points represent quarterly 95^th^ percentile values for each site and period (baseline or dredge).

Detailed statistical analysis incorporating spatial and temporal variability indicated that for the Barrow Island project there was strong evidence of an effect of distance from dredging for all the water quality metrics examined including those based on turbidity (Fig 4, Table 1A) and light (Fig 4, Table 1B, see also S1 Table). Relationships with distance were relatively strong, with 36 to 55% of the variance explained by the best model fit (Table 1). Most of the turbidity-based water quality metrics showed a significant three-way interaction effect between baseline/dredging, North/South and distance (Table 1), with no discernible relationship with distance occurring for the baseline data, a very sharp relationship occurring for sites north of the dredging site, and a strong but more gradual relationship occurring for the southern sites during the dredging period (Fig 4, Table 1, S1 File). Effects of an impact on water quality were evident at distances of up to only 2.1 km for the Northern sites at Barrow Island, whereas the Southern sites appeared to show evidence of an effect of distances of up to 20 km (Fig 4, Table 1, S1 File).

**Table 1:** Distance from the dredging activity relationships for the Barrow Island project. Shown are results for 11 turbidity (NTU) and 6 light based water quality metrics. The notation of *P*_80_ and *P*_95_ represents the 80^th^ and 95^th^ percentiles. Shown are the ‘best’ model as selected by AICc (see methods for more details), R^2^ values, along with estimated distance of effects and power decay functions (Equation; in the form a*d^-b^, where d is distance from the primary dredging activity), divided into spatial components (N-S—North or South, E—W–East or West) where required according to the best model. The distance of effect values represent the distance at which the fitted curve falls below the 80^th^ percentile of the baseline value (i.e. the dredging effect becomes negligible). Notation for the ‘best’ model are as follows: NS—a North versus South factor; EW—an East versus West fixed factor; Dr—a factor delineating the pre-dredge versus during dredging; Di—a continuous predictor representing the distance from dredging; Dep—a continuous predictor representing the depth of sites; “:” indicates an interaction among the predictors.

Metric	Best Model	R^2^	Distance (km)	Equation
**Turbidity (NTU)**				
Mean daily	NS:Dr+Di:NS:Dr	0.46	1.9(N); 15.6(S)	11.8d^−1.42^(N); 12.6d^−0.22^(S)
Running 7 d mean	NS:Dr+Di:NS:Dr	0.48	1.9(N); 15.9(S)	10.3d^−1.54^(N); 10.8d^−0.23^(S)
Running 14 d mean	NS:Dr+Di:NS:Dr	0.48	1.9(N); 13.2(S)	8.2d^−1.32^(N); 8.6d^−0.23^(S)
Running 7 d median	NS:Dr+Di:NS:Dr	0.44	2.0(N); 12.4(S)	8.6^−1.47^(N); 9.1d^−0.26^(S)
Running 14 d median	NS:Dr+Di:NS:Dr	0.47	2.1(N); 10.5(S)	5.8d^−0.88^(N); 6.9d^−0.26^(S)
Running 7 d *P*_80_	NS:Dr+Di:NS:Dr	0.44	1.8(N); 16.5(S)	15.0d^−1.64^(N); 15.6d^−0.22^(S)
Running 14 d *P*_80_	NS:Dr+Di:NS:Dr	0.47	1.9(N); 13.3(S)	11.7d^−1.39^(N); 12.5d^−0.25^(S)
Median daily	NS:Dr+Di:NS:Dr	0.36	1.6(N); 19.6(S)	7.3d^−1.64^(N); 9.0d^−0.12^(S)
Daily *P*_80_	NS:Dr+Di:NS:Dr	0.46	1.8(N); 15.5(S)	17.3d^−1.70^(N); 19.1d^−0.24^(S)
Daily *P*_95_	NS:Dr+Di:NS:Dr	0.53	2.1(N); 13.6(S)	37.4d^−1.12^(N); 33.2d^−0.35^(S)
Daily maximum	EW:Dr+Di:EW:Dr	0.55	1.9(E); 8.8(W)	44.4d^−0.52^(E); 63.3d^−0.54^(W)
**Light (DLI)**				
Mean	Dep+Dr+Di:Dr	0.40	4.6	1.35d^0.28^
7 d running mean	Dep+Dr+Di:Dr	0.48	3.3	2.02d^0.26^
14 d running mean	Dep+Dr+Di:Dr	0.49	2.6	2.35d^0.25^
Proportion d <5	Dep+ NS:Dr+Di:NS:Dr	0.44	1.9(N); 12.6(S)	0.50d^−0.25^(N); 0.59d^−0.18^(S)
Proportion d <10	Dep+NS:Dr+Di:NS:Dr	0.46	1.6(N); 13.3(S)	0.59^−0.23^(N); 0.69d^−0.16^(S)
Proportion d <15	Dep+NS:Dr+Di:NS:Dr	0.45	1.6(N); 12.6(S)	0.64d^−0.20^(N); 0.74d^−0.15^(S)
Proportion d <20	Dep+NS:Dr+Di:NS:Dr	0.44	1.5(N); 11.6(S)	0.68d^−0.17^(N); 0.79d^−0.13^(S)

**Fig 5:**
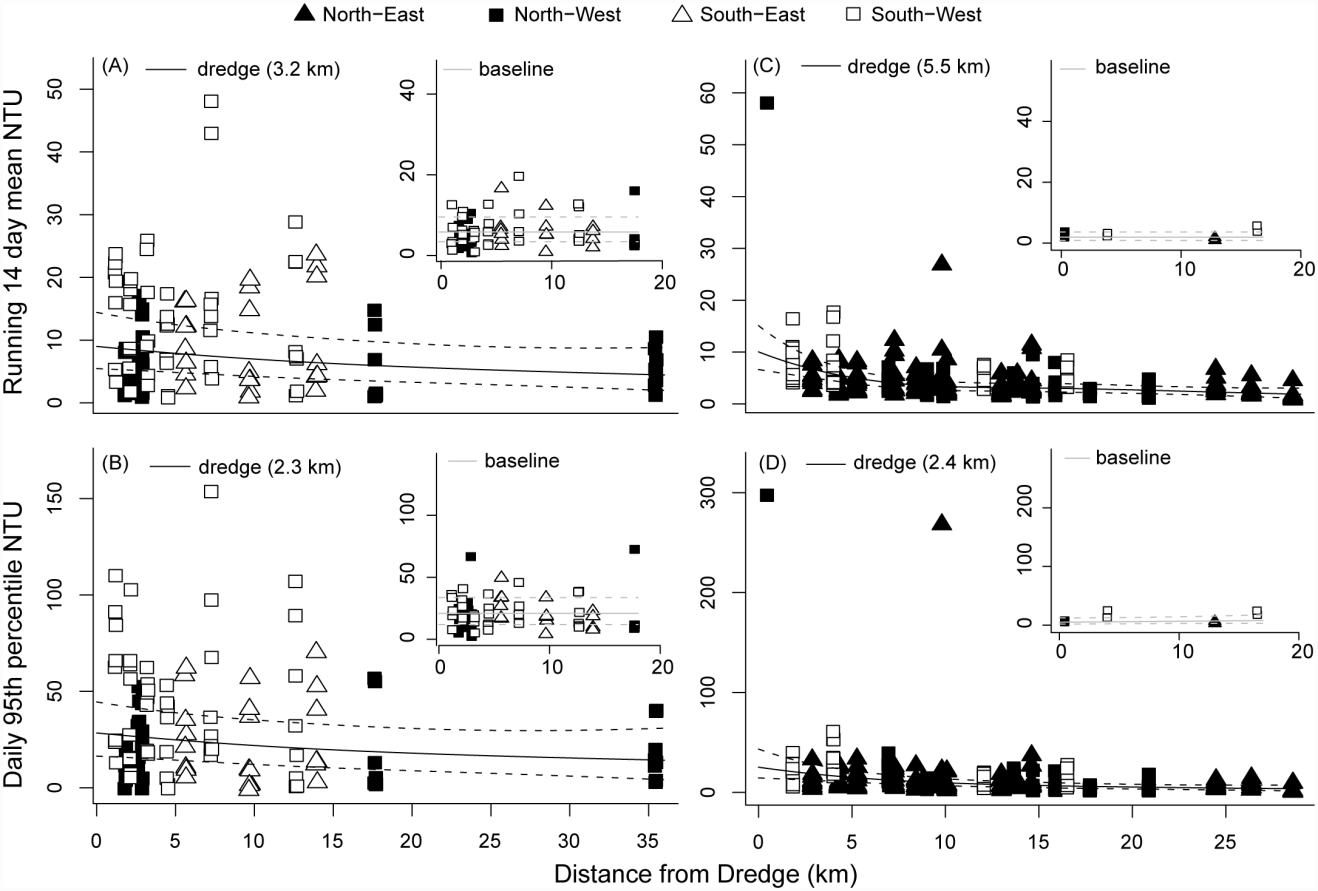
Distance decay relationships for the Cape lambert and Burrup Peninsula dredging projects. Shown are the running 14 day mean turbidity (NTU, A) and daily 95^th^ percentile of turbidity (B) at Cape Lambert, and the running 14 day mean turbidity (C) and daily 95^th^ percentile of turbidity (D) at Burrup Peninsula. Fitted curves represent fitted best fit Generalised Additive Mixed Models ± 95% confidence bounds. Baseline and dredge periods were fitted as a two way interaction with distance from dredge, or as a three way interaction as appropriate (North/South or East/West of the location of the primary dredging activity, see methods for further details). Values in parentheses indicate the distance at which the fitted curve falls below the 80^th^ percentile of the baseline value (i.e. the dredging effect becomes negligible).

Distances from dredging relationships were much weaker for the Cape Lambert and Burrup Peninsula projects (Fig 5, Table 2, S1 File). For the Cape Lambert project, R^2^ values were exceptionally low (<16% of the variance explained across all metrics) and the best fit models tended to delineate patterns in space rather than an effect of distance to dredging (Fig 5, Table 2A). Baseline data was sparse for the Burrup Peninsula project, as was data at sites very close to the primary dredging activity (S1 Table). What data there is available indicates a potential East/West interaction during the dredging period, with highly elevated turbidity close to the dredge activity for the Western sites, although this relationship is driven by a single point (CHC4, early in 2008). Estimated distances of impact for Burrup Peninsula ranged from 2.1 to 6.0 km, depending on the metric examined (Fig 5, Table 2).

**Table 2:** Distance from the primary dredging activity relationships. Shown are results for 11 turbidity (NTU) based water quality metrics for the Cape Lambert (A) and Burrup peninsula (B) projects. The notation of *P*_80_ and *P*_95_ represents the 80^th^ and 95^th^ percentiles. Shown are the ‘best’ model as selected by AICc (see methods for more details), R^2^ values, along with estimated distance of effects (Distance) and power decay functions (Equation; in the form a.d-b, where d is distance from the primary dredging activity), divided into spatial components where required according to the best model. The distance of effect values represent the distance at which the fitted curve falls below the 80^th^ percentile of the baseline value (i.e. the dredging effect becomes negligible). Notation for the ‘best model’ are as follows: NS—a North versus South factor; EW—an East versus West fixed factor; Dr—a factor delineating the pre-dredge versus during dredging; Di—a continuous predictor representing the distance from dredging; Dep—a continuous predictor representing site depth; “:” indicates an interaction among the predictors.

Turbidity Metric (NTU)	Best Model	R^2^	Distance (km)	Equation
(**A) Cape Lambert—turbidity**				
Mean daily	NS	0.09		20.4d^−0.13^
Running 7 d mean	NS+EW	0.10		15.6d^−0.11^
Running 14 d mean	NS+EW	0.10	2.3	12.4d^−0.11^
Running 7 d median	NS+EW	0.12		13.7d^−0.13^
Running 14 d median	NS	0.09		9.6d^−0.12^
Running 7 d *P*_80_	NS+EW	0.09		25.9d^−0.10^
Running 14 d *P*_80_	EW+NS:Dr	0.16	2.7	19.0d^−0.09^
Median daily	NS	0.10		17.9d^−0.12^
Daily *P*_80_	NS	0.08	0.5	28.5d^−0.14^
Daily *p*95	NS+EW	0.09	2.3	44.6d^−0.16^
Daily maximum	NS:Dr	0.09	2.0	58.4d^−0.16^
**(B) Burrup Peninsula**				
Mean daily	EW:Dr+Di:EW:Dr	0.21	5.0 (W)	8.8d^−0.27^ (W)
Running 7 d mean	Di+NS	0.26	5.1	24.0d^−0.88^
Running 14 d mean	Dr+Di:Dr	0.21	5.5	21.5d^−0.97^
Running 7 d median	Di+NS	0.28	4.5	23.5d^−1.07^
Running 14 d median	EW:Dr+Di:EW:Dr	0.29	4.7 (W)	4.6d^−0.27^(E); 19.2d^−1.15^(W)
Running 7 d *P*_80_	Di+NS	0.21	6.0	32.6d^−0.81^
Running 14 d *P*_80_	Di+NS	0.23	5.5	26.6d^−0.90^
Median daily	EW:Dr+Di:EW:Dr	0.20	4.1 (W)	7.1d^−0.28^(E); 24.1d^−1.07^ (W)
Daily *P*_80_	EW:Dr+Di:EW:Dr	0.19	3.9 (W)	11.8d^−0.28^ (W)
Daily *P*_95_	Dr+Di:Dr	0.19	2.1	95.2d^−1.25^
Daily maximum	Dr+Di:Dr	0.18	2.6	139.6d^−1.30^

### Detailed plume analysis at Barrow Island

**Fig 6:**
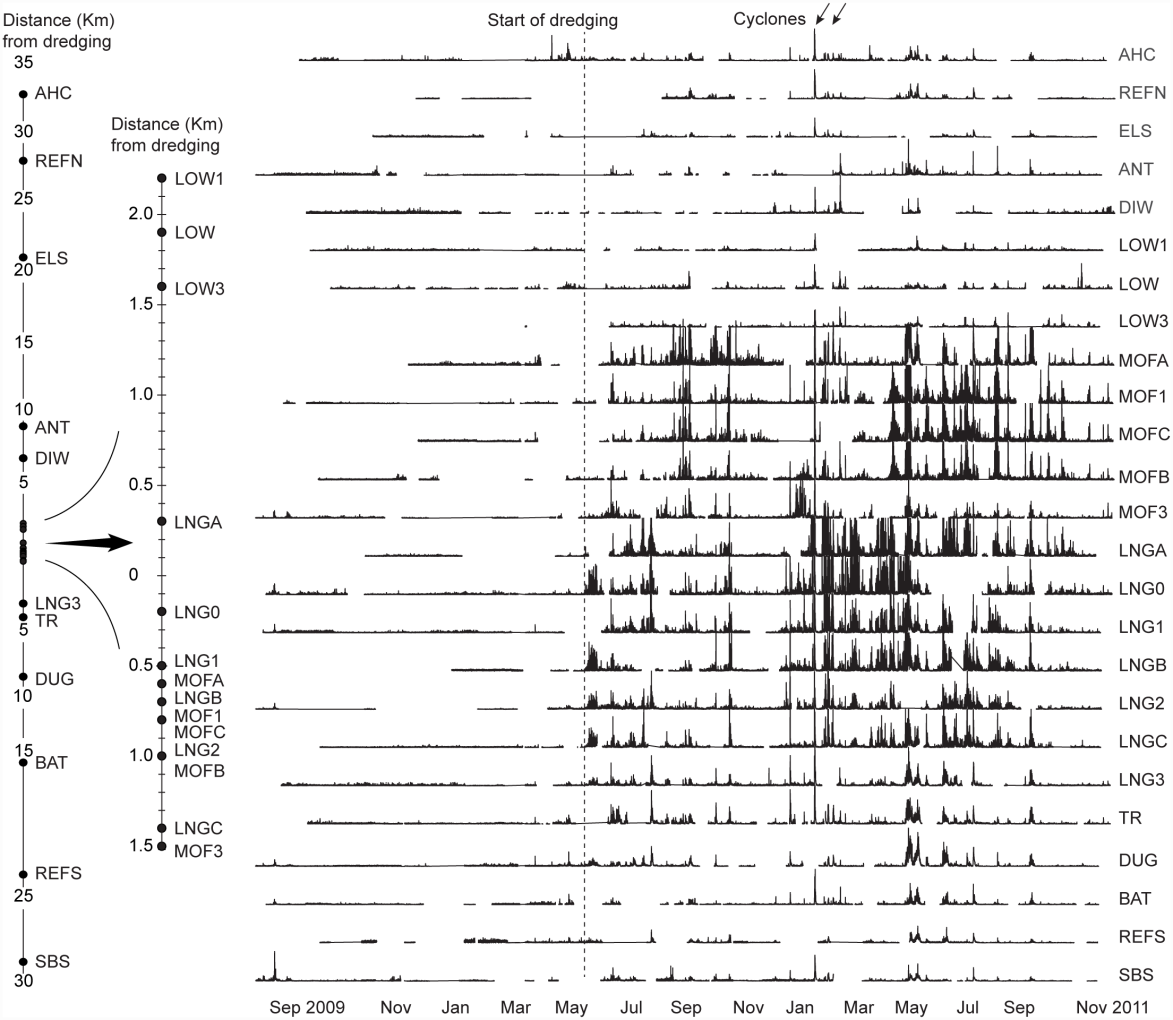
Turbidity time series for the Barrow Island project. Shown are turbidity (NTU) measured every 10 mins from September 2009 to November 2011 at 25 water quality monitoring sites located from ~30 km north to ~30 km south of the main dredging areas (see Fig 1 for sites names and details). Gaps in the data represent occasional failure of the loggers. Each figure is scaled identically from 0–100 NTU. Occasionally readings exceed 100 NTU (see [21] and S1 Table contains full, non-truncated summary statistics).

The predominantly southerly movement of the dredge plume during the dredging project at Barrow Island, as well as the overall temporal variability in plume extent, can be seen through the sequential time series of the turbidity data across the sites from the north of Barrow Island (the AHC, REFN, ELS, ANT, and LOW sites), through the region of high dredging activity (the MOF and LNG sites) and down through the southern sites (the TR, DUG, BAT, REFS and SBS sites; Fig 6). The time series shows some periods where the turbidity is relatively widespread across many sites, extending to both northern and southern control sites. This is likely to be associated with storm events such as the one occurring in late February associated with tropical cyclone Carlos. At other times the turbidity events are highly contracted, impacting only those sites close to the dredging, and are clearly the result of dredging plumes (Fig 6)

**Fig 7:**
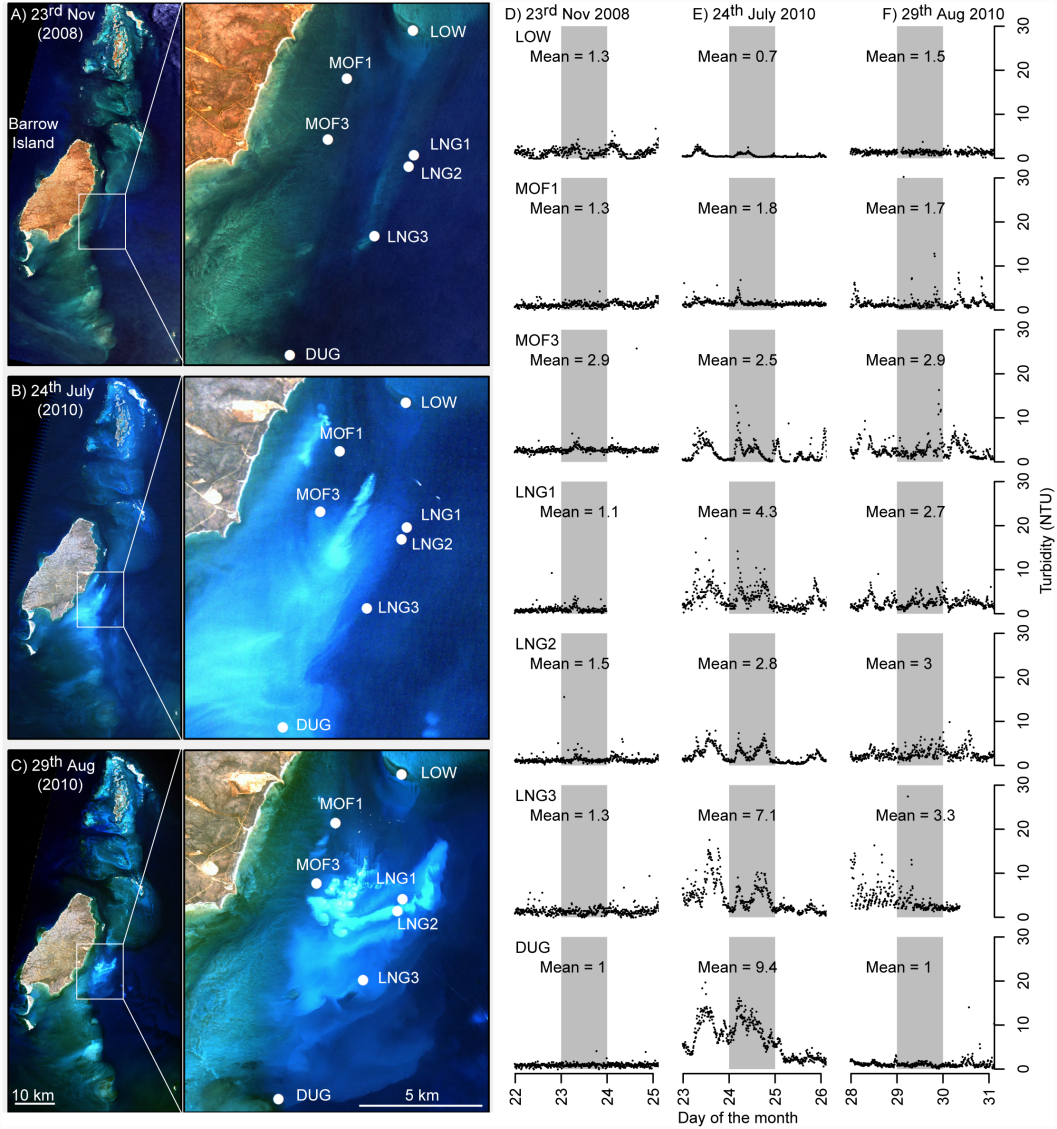
A comparison of satellite images and turbidity. Images are shown for three periods during the Barrow Island dredging program, taken on: (A) 23^rd^ of November 2008 (baseline phase), (B) 24^th^ of July 2010 (dredging phase), and (C) 29^th^ August 2010 (dredging phase). Images from (A) and (C) were sourced from the Japan Aerospace Exploration Agency (JAXA) Advanced Land Observing Satellite (ALOS) Advanced Visible and Near Infrared Radiometer type 2 (AVNIR-2) satellite. image (10 m pixel resolution). The image in (B) was sourced from the Landsat 5 Thermal Mapper (Path/Rows 114/74-75) 30 m resolution (courtesy of the U.S. Geological Survey)(see also Fig 1 for sites names). Turbidity data (NTU) are shown for the three days surrounding the image date for each image (D, E and F), including all sites for which there were data across all three periods. The grey shaded area indicates the data for the specific date of each image.

The satellite imagery shows that during the dredging period there were clearly visible plumes which generally travelled in a southerly direction, (Fig 7B & 7C). In the July 2010 image, the plume was relatively widespread and well mixed, with clear evidence of high suspended sediment concentrations near the primary dredging activity as well as at sites as far away as DUG (~9 km, with mean of 9.4 NTU), followed by LNG3 (mean of 7.1 NTU) and LNG1 (mean of 4.3 NTU; Fig 7B & 7E). In the August 2010 image the plume was highly spatially complex, and despite being readily apparent on satellite imagery, resulted in only marginal increases in turbidity across the sites (Fig 7F).

**Fig 8:**
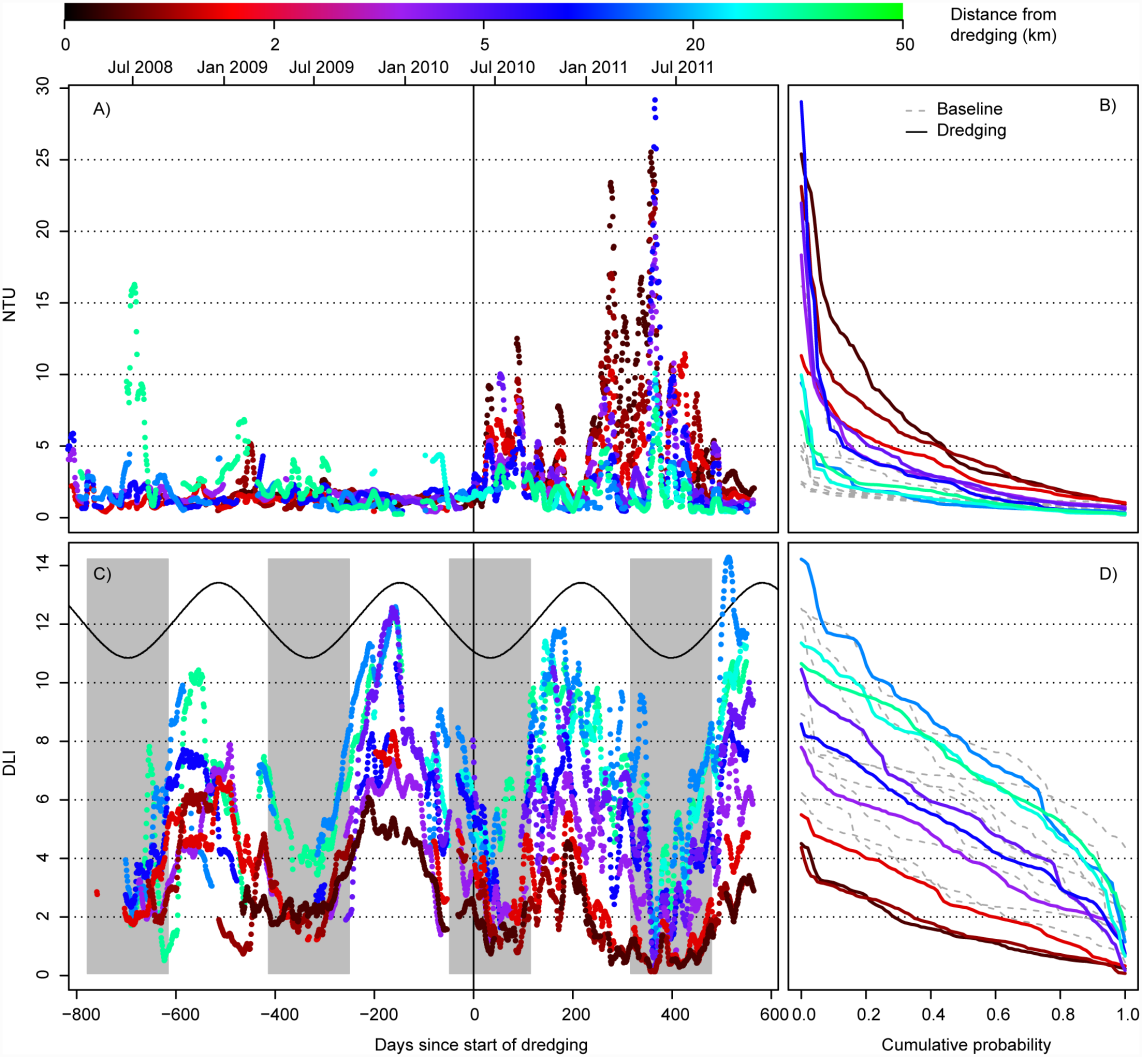
Daily time series and cumulative probability plots. Shown are running 14 day mean turbidity (NTU; A and C) and light (DLI; B and D), with a colour ramp indicating relative distance from dredging activity. Only sites south of the LNG dredging activity (see Fig 4) are included to aid figure clarity. Grey panels indicate the six shortest-day months of the year, based on sun-rise and sun-set data in the region.

The relatively systematic decline in water quality impacts from dredging across these southern sites at Barrow Island can be seen clearly in daily time series data for both turbidity and light across the southern transect of sites at Barrow Island; with very high peaks in turbidity (Fig 8A) and associated declines in light evident throughout the dredging period (Fig 8B). There was a clear shift across this southern transect in terms of the cumulative probability distribution curves for both turbidity (Fig 8C) and light (Fig 8D), with dredging causing a positive shift in turbidity (Fig 8C) and a negative shift in light (Fig 8D) across the full range of probabilities.

The high turbidity during the dredging period resulted in sites close to the dredging activity having DLI levels of <2 mol photons m^-2^ for up to 80% of the time, with values of less than 4 mol photons m^-2^ being relatively commonplace (Fig 8D). There is a clear seasonal pattern in light levels following annual changes in daylight hours, with the low light conditions associated with high turbidity being most pronounced during the already lower light winter months (Fig 8C). Importantly, even for a strongly directionally biased plume such as that seen in the Barrow Island project, the effects of dredging appear to decline relatively rapidly with distance, with impacts becoming minimal at distances of around 5 km, and completely indistinguishable from baseline at distances of ~15–20 km (Fig 8).

## Discussion

This is one of the first published studies to examine in detail the spatial impacts of large scale capital dredging operations in a tropical, coral reef setting. Overall there was strong evidence of a relationship with distance from dredging with all the water quality metrics examined, particularly for the dredging program at Barrow Island. The impacts of dredging followed a steep power-law decay relationship, with sites near dredging experiencing much greater changes to water quality than the more distant ones, supporting the use of spatial zoning to manage dredging projects [17,33]. The study has also provided valuable information of water quality conditions during large scale capital dredging operations, allowing the design of future studies on the effects of turbidity on tropical species using environmental relevant or realistic exposure scenarios (see [61]).

How far dredging plumes can travel has important implications for the EIA process and compliance water quality and biological monitoring programs. Recently Evans et al. (2012) visually interpreted MODIS images to map the dredge plume boundaries in the shallow, clear water environment of the Barrow Island project. Their analyses showed that occasionally sediment plumes could be observed over 30 km away from the dredging activities. Such observations define a ‘zone of influence’ i.e. areas where changes in turbidity can occur, but are not necessarily associated with detectable impacts on the benthic biota. Aerial and satellite images are able to detect very small quantities of suspended material if the turbid water is juxtaposed to clear oceanic water. The blue light scattering from the oceanic water can contrast very strongly with the integrated scattering of sediment and organic material over the water column due to subtle changes in ocean colour. During the EIA process, zones of influence are often predicted (by modelling) and the primary reason is so that authorities can be made aware beforehand of potential social issues such as plumes impacting swimming beaches or marine recreational areas. However, at the outer limits of the zone suspended sediment concentrations are, by definition, at the limits of the detection techniques, and are likely to be very low and within the range of turbidity naturally experienced during wind and wave events. It is questionable whether such weak plumes will exert any significant biological effects; An unintended consequence, however, could be a public misconception of the scale of potential deleterious effects (for further discussion of the issue see [29]).

For the purpose of defining the extent of the plume footprint in this study, we used a criterion where the value of the fitted curve (representing a median) intersects the 80^th^ percentile (*P*_80_) of the baseline value for turbidity (or the 20^th^ percentile for the light data). This comparison procedure (*P*_50_–*P*_80_) has its origins in the Australia and New Zealand water quality guidelines for fresh and marine waters [59]. The basis is somewhat arbitrary but also pragmatic and associated with a notion of the developers that a median value at an impact site above the 80^th^ percentile of a reference site represents a ‘measurable perturbation’, and thus worth investigating [59]. The approach is nevertheless useful as it links water quality with the possibility of ecological change and is also based on a relative change rather than an absolute value [62]. In this study the *P*_50_–*P*_80_ approach was compared to pre-dredging baseline period (as opposed to comparing to control of reference sites) and impacts of dredging on water quality appear to extend distances of ~3 km from the dredging, although in one instance extended as far as 15–20 km. The larger estimate for potential distances of measurable effect occurred during the Barrow Island project, where local oceanographic features produced an unusual pattern of a near unidirectional flow southwards over the duration of the project, with minimal days of northward movement. This pattern resulted in the significant three-way interaction between the baseline-versus-dredging periods, distance from dredging, and a north versus south characterisation of sites. The outcome of interaction was that there was a slower decline of water quality with distance south of the primary dredging area (*P*_50_–*P*_80_ distances of 8.8–19.6 km), with a correspondingly much faster decline in the north (*P*_50_–*P*_80_ distance of 1.5–2.1 km).

Overall the strength of the relationship with distance from dredging was much weaker for the Cape Lambert and Burrup Peninsula projects. The general dredging activity may have been less concentrated given the length of the shipping channels. Both locations are also nearer the mainland and likely to show stronger underlying onshore-offshore gradients in water quality that may have masked patterns associated with dredging. Also, there were much fewer water quality monitoring sites close to the dredging activities in the projects because the regulatory conditions at the time were most concerned with establishing that water quality and ecological change did not occur at more distant sites, than showing effects did occur close to dredging where habitat loss was allowed. This policy direction has recently changed (see [17]).

The spatial analysis carried out here are based on a range of metrics that capture site level summaries across time using a range of temporal scales (hours, days, weeks) and summary metrics (e.g. means, percentiles). However, it is important to remember that such metrics do not necessarily capture the realised *in-situ* water quality conditions across all sites at instantaneous time-scales. While the distance from dredging activity plots may seem relatively consistent once potential effects of overall plume direction are taken into account, the reality is that at any given time turbidity plumes appear to be highly spatially heterogeneous as clearly shown in the satellite images (Fig 7B & 7C). A peak in turbidity occurring at one location may not be evident at sites only a few hundred metres away. High levels of variation among sites within regions appears to be a consistent feature of turbidity data [63]. Fine scale spatial structure in turbidity raises two issues with respect to dredging management and monitoring that have not yet been thoroughly addressed. First is the issue of whether previously adopted water quality monitoring designs are spatially sufficient, or should more effort be made to establish more optimal designs (e.g. spatially hierarchical and/or stratified sampling [64] or grid sampling [65]) that may be better suited to demonstrating dredging impacts. While power analysis [66–68], principles of optimal sampling design [64,69–71] and before-after-control-impact assessment [72,73], as well as cost benefit analysis [74] are widespread in ecology, such principles are not often applied to water quality sampling. Second is the issue that if there is poor temporal correlation in water quality readings among sites even at relatively small spatial scales, monitoring protocols and threshold values based on the use of comparisons to control or reference sites may be of limited value unless extreme care is taken to ensure they adequately represent the impact locations [39].

The focus of this study has been the spatial effects from the excavation itself (including spillage from drags heads and hopper overflow). However, disposal of sediments at offshore dredge material placement sites (spoil grounds) is also a significant turbidity-generating activity associated with dredging. Preliminary analyses were carried out to attempt to examine patterns in turbidity with distance from spoil disposal sites across the three studies, and no strong relationships were revealed. Admittedly, however, none of the three projects had a sampling design that was spatially designed for looking at effects of distance from the placement sites, rendering the conclusion of such analyses relatively weak. The effect of the disposal at Barrow Island can be seen in the satellite images in Fig 7B & 7C (bottom left hand corner of enlarged panels), and generally appears relatively minor compared to the turbidity generated at the point of excavation. For the Barrow Island project the spoil disposal site was situated to the south east of the dredging activities and may in fact partially account for some of the southerly extent of the Barrow Island dredge plume. In this context the distance analysis reported here potentially represents the total effect of the whole dredge operation (both excavation and disposal), with anything over ~15–20 km not affected.

### Water Quality thresholds for reef biota

The *P*_50_–*P*_80_ approach of ANZECC/ARMCANZ to estimate distances of detectable effects is recommended where information on biological responses is absent, and is considered to be reasonably conservative. Other statistical criteria based on water quality could be used, that might yield substantially different estimated distances. For example, it could be defined as the distance at which the predicted (best fit value, representing a mean or median) crosses the upper 95^th^ percentile value of the baseline state. Such a definition would likely yield shorter distances of potential impact than currently reported here.

What is really needed to define the distance of effects are water quality thresholds which relate changes in the physical parameters (light reduction, total suspended sediment, sediment deposition) to biological responses (sublethal and lethal) of the underlying organisms. Such thresholds are not yet available for reef biota such as coral, seagrasses and filter feeders and require laboratory and/or manipulative field based studies and subsequent verification before being used. The spatial analyses described here and the temporal analyses described in Jones et al. [21] have however provided some insights into the problems that need to be addressed when developing such thresholds, and especially how to incorporate exposure across varying temporal scales. For example, during the Barrow Island project, >50% of the daily light integrals were very low (i.e. <1.5 mol photons m^-2^) at sites within a few hundred metres of the dredging, as opposed to 3–8 mol photons m^-2^ during the baseline period. Clearly light was affected by dredging but it is very significant for the underlying communities whether these low light values occur at once or intermittently. Theoretically, an intermittent pattern could afford the opportunity for primary producers such as corals to recover energy deficits between the low light periods. This has already been suggested as a mechanism for how corals survive natural resuspension events ([9,75]). Simple inspection of the data shows many low light days occurred in a near continuous block in the winter period, where a combination of low seasonal light availability and more intense turbidity generating events resulted in a 6 month period of DLIs <1 mol photons m^2^. The pattern suggests one possible management practice could be timing maintenance and/or short-term capital dredging programs to avoid seasonal lows in light availability if light is considered a key pressure parameter (i.e. dredging near seagrass beds). However the data also suggests that analyses of water quality data using the whole dredging or baseline periods using cumulative probability plots (see Fig 8) although instructive for characterizing effects on a broad scale, is much too coarse for threshold development.

The recent study of the temporal patterns of changes in water quality close to dredging indicated that dredging changes the overall probability distribution of turbidity values and the upper/lower percentile values (e.g. 99^th^, 95^th^ for NTU or 1^st^ 5^th^ for light) were highly elevated/lowered over short periods, but converged to values close to the baseline states over longer periods (weeks to months) [21]. The running means calculated across multiple time periods (from hours to a month), summarized across a broad range of percentiles values [21], and expressed in terms of distance from the dredging activities (this study) has provided a matrix of environmentally realistic exposure conditions that can be used to explore lethal and sub-lethal water quality thresholds in future laboratory- and field-based manipulative studies (see online S1 Table). This could ultimately lead to a more accurate definition of the potential ecological footprint of plumes from dredging projects than the *P*_50_–*P*_80_ approach used here or other statistical approaches.

The three projects described here spanned a range of environmental settings including an offshore, ‘clear water’ environment (Barrow Island), an exposed nearshore cape or headland (Cape Lambert), and an enclosed inshore turbid reef environment (Mermaid Sound, Burrup Peninsula). Nevertheless, the patterns of turbidity generation will be highly site and project specific and will vary with production rates (volumes dredged) and dredge types (cutter suction dredge versus back hoe or TSHD) and methodology used (overflow etc). Other factors include the nature of the sediments being dredged and the oceanographic conditions such as tidal and current strengths and wind- and wave-induced resuspension associated with seabreezes. For the upper (15–20 Km) bound identified for the Barrow Island project, it should be recognized that was a very large scale capital dredging operation (8 Mm^3^) with multiple dredges working 24 a day, in a clear water environment, and with the unusual oceanographic feature of unidirectional flow. As such, we consider that the southerly extension of the plume represents an upper bound on the distances at which dredging might be expected to cause ‘measurable perturbations’ as defined by the *P*_50_–*P*_80_ approach.

## Supporting Information

S1 File Detailed results. Full subsets best model output (Tables A-C) and plotted best model fits (Figures A-D) for all variables examined statistically for distance decay relationships for each of the three dredging projects in the Pilbara.
(PDF)

S1 Table Detailed summary data. Max, 99th, 95th, 80th percentiles, median and mean NTU values over 1 h, 1 d, 14 d, and 21 d running average period at all sites during the baseline period or for during the duration of the dredging program.
(PDF)
