# Supplementary material for: Spatial Patterns in Water Quality Changes during Dredging in Tropical Environments
Source: PLoS One. 2015 Dec 2;10(12):e0143309. doi: 10.1371/journal.pone.0143309 (PMC4667927; doi:10.1371/journal.pone.0143309)
Supplement: S1 File — Full subsets best model output (Tables A-C) and plotted best model fits (Figures A-D) for all variables examined statistically for distance decay relationships for each of the three dredging projects in the Pilbara. (PDF) [file pone.0143309.s001.pdf]

S1 File - Detailed distance decay analysis results

Supplementary Tables 1.A through to 1.C show the full subsets best model output for all variables examined statistically for distance decay relationships for each of the three dredging projects in the Pilbara. The optimal model is considered that within 2 AICc of that model with the lowest AICc value that has the fewest parameters (Burnham & Anderson 2002). Where there was more than one model within 2 AICc of the best model (that with the lowest AICc), all models within 2 AICc are shown. In this instance the ωi values show the relative AICc weight of each model, which is an indication of the relative strength of evidence for a given model in the complete model set. Supplementary Figures 1.A through to 1.D show plotted best model fits.

References

R Core Team (2014) R: A language and environment for statistical computing. R Foundation for Statistical Computing, Vienna, Austria. URL http://www.R-project.org/.

Burnham KP, Anderson DR (2002) Model Selection and Multimodel Inference; A Practical Information-Theoretic Approach. Springer, New York

ESRI (2013) ArcGIS Desktop, v10.2. . Redlands CA, Environmental Systems Research Institute

Wood S, Scheipl F (2013) gamm4: Generalized additive mixed models using mgcv and lme4. R package version 0.2-2. http://CRANR-projectorg/package=gamm4

Table S1.A. Generalised Additive Mixed Models (GAMM) results for the Barrow Island project. Shown are all top models for each response variable examined, defined as those models within 2 AICc of the best model. ωi values show the relative weight of evidence for each model.

Response metric	Top models	AICc	BIC	ωi	Number of parameters
Mean NTU	NS:Dredging+Distance:NS:Dredging	-152.7	-111.7	1.00	11
Running 7 day mean NTU	NS:Dredging+Distance:NS:Dredging	-164.5	-123.4	1.00	11
Running 14 day mean NTU	NS:Dredging+Distance:NS:Dredging	-213.8	-172.7	1.00	11
Running 7 day median NTU	NS:Dredging+Distance:NS:Dredging	199.6	240.7	1.00	11
Running 14 day median NTU	NS:Dredging+Distance:NS:Dredging	100.8	141.9	0.94	11
Running 7 day 80th percentile NTU	NS:Dredging+Distance:NS:Dredging	-50.9	-9.8	1.00	11
Running 14 day 80th percentile NTU	NS:Dredging+Distance:NS:Dredging	-111.2	-70.1	1.00	11
Median NTU	NS:Dredging+Distance:NS:Dredging	-213.4	-172.4	1.00	11
80th percentile NTU	NS:Dredging+Distance:NS:Dredging	-102.4	-61.4	1.00	11
95th percentile NTUs	NS:Dredging+Distance:NS:Dredging	15.5	56.5	1.00	11
Maximum NTU	EW:Dredging+Distance:EW:Dredging	31.1	72.1	0.53	11
	NS:Dredging+Distance:NS:Dredging	31.6	72.6	0.41	11
Mean DLI	Depth+Dredging+Distance:Dredging	-48.6	-21.1	0.59	8
	Depth+NS:Dredging+Distance:NS:Dredging	-47.6	-6.7	0.35	12
7 day running mean DLI	Depth+Dredging+Distance:Dredging	-128.0	-100.5	0.91	8
14 day running mean DLI	Depth+Dredging+Distance:Dredging	-365.5	-337.9	0.91	8
Proportion day <5 µmol photons m^{-2} s^{-1}	Depth+NS:Dredging+Distance:NS:Dredging	-623.0	-582.1	1.00	12
Proportion day <10 µmol photons m^{-2} s^{-1}	Depth+NS:Dredging+Distance:NS:Dredging	-614.0	-573.1	1.00	12
Proportion day <15 µmol photons m^{-2} s^{-1}	Depth+NS:Dredging+Distance:NS:Dredging	-604.6	-563.7	0.99	12
Proportion day <20 µmol photons m^{-2} s^{-1}	Depth+NS:Dredging+Distance:NS:Dredging	-443.6	-402.7	0.96	12

Table S1.B. Generalised Additive Mixed Models (GAMM) results for the Cape Lambert project. Shown are all top models for each response variable examined, defined as those models within 2 AICc of the best model. ωi values show the relative weight of evidence for each model.

Response metric	Top models	AICc	BICc	ωi	Number of parameters
Mean NTU	NS+EW:Dredging	-52.7	-28.0	0.32	8
	NS+EW	-51.6	-32.9	0.18	6
	NS	-51.5	-35.9	0.17	5
Running 7 day mean NTU	EW+NS:Dredging	-69.4	-44.6	0.36	8
	NS+EW	-68.6	-49.9	0.25	6
Running 14 day mean NTU	EW+NS:Dredging	110.0	134.8	0.52	8
	NS:Dredging	112.0	133.7	0.20	7
Running 7 day median NTU	EW+NS:Dredging	-42.1	-17.4	0.30	8
	NS+EW	-41.7	-23.0	0.25	6
	NS:Dredging	-40.2	-18.5	0.12	7
Running 14 day median NTU	EW+NS:Dredging	-60.3	-35.6	0.26	8
	NS:Dredging	-59.8	-38.0	0.19	7
	NS+EW	-59.3	-40.6	0.16	6
	NS	-58.9	-43.3	0.13	5
Running 7 day 80th percentile NTU	EW+NS:Dredging	-11.1	13.6	0.37	8
	NS+EW	-10.2	8.5	0.23	6
Running 14 day 80th percentile NTU	EW+NS:Dredging	198.4	223.2	0.62	8
Median NTU	NS+EW:Dredging	-56.4	-31.7	0.25	8
	NS	-56.0	-40.4	0.20	5
	NS+EW	-55.9	-37.2	0.19	6
	Distance+NS	-54.8	-36.1	0.11	6
80th percentile NTU	NS+EW:Dredging	227.3	252.1	0.30	8
	NS+EW	228.1	246.8	0.20	6
	NS	229.0	244.7	0.13	5
95th percentile NTUs	NS+EW:Dredging	295.9	320.7	0.34	8
	NS+EW	297.0	315.7	0.20	6
Maximum NTU	EW+NS:Dredging	331.1	355.9	0.37	8
	NS:Dredging	332.3	354.0	0.21	7

Table S1.C. Distance from dredging Generalised Additive Mixed Models (GAMM) results for the Burrup Peninsula project for all response metrics examined. Shown are all top models for each response variable examined, defined as those models within 2 AICc of the best model. ωi values show the relative weight of evidence for each model.

Response metric	Top models	AICc	BICc	ωi	Number of parameters
Mean NTU	EW:Dredging+Distance:EW:Dredging	-224.8	-192.5	0.74	9
Running 7 day mean NTU	Distance+NS	-230.7	-209.0	0.38	6
	Dredging+Distance:Dredging	-229.8	-204.6	0.24	7
	EW:Dredging+Distance:EW:Dredging	-229.7	-197.4	0.23	9
Running 14 day mean NTU	EW:Dredging+Distance:EW:Dredging	-285.7	-253.4	0.62	9
	Dredging+Distance:Dredging	-284.0	-258.8	0.26	7
Running 7 day median NTU	EW:Dredging+Distance:EW:Dredging	-239.2	-206.9	0.39	9
	Distance+NS	-238.5	-216.8	0.27	6
	Dredging+Distance:Dredging	-237.7	-212.4	0.18	7
Running 14 day median NTU	EW:Dredging+Distance:EW:Dredging	-311.2	-278.8	0.84	9
Running 7 day 80th percentile NTU	Distance+NS	-102.4	-80.7	0.66	6
Running 14 day 80th percentile NTU	Distance+NS	-191.0	-169.3	0.34	6
	Dredging+Distance:Dredging	-190.4	-165.1	0.26	7
	EW:Dredging+Distance:EW:Dredging	-190.0	-157.7	0.21	9
Median NTU	EW:Dredging+Distance:EW:Dredging	-282.5	-250.2	0.65	9
80th percentile NTU	EW:Dredging+Distance:EW:Dredging	-178.4	-146.0	0.78	9
95th percentile NTUs	EW:Dredging+Distance:EW:Dredging	-44.7	-12.4	0.58	9
	Dredging+Distance:Dredging	-43.8	-18.6	0.37	7
Maximum NTU	EW:Dredging+Distance:EW:Dredging	-34.7	-2.4	0.62	9
	Dredging+Distance:Dredging	-33.5	-8.3	0.34	7

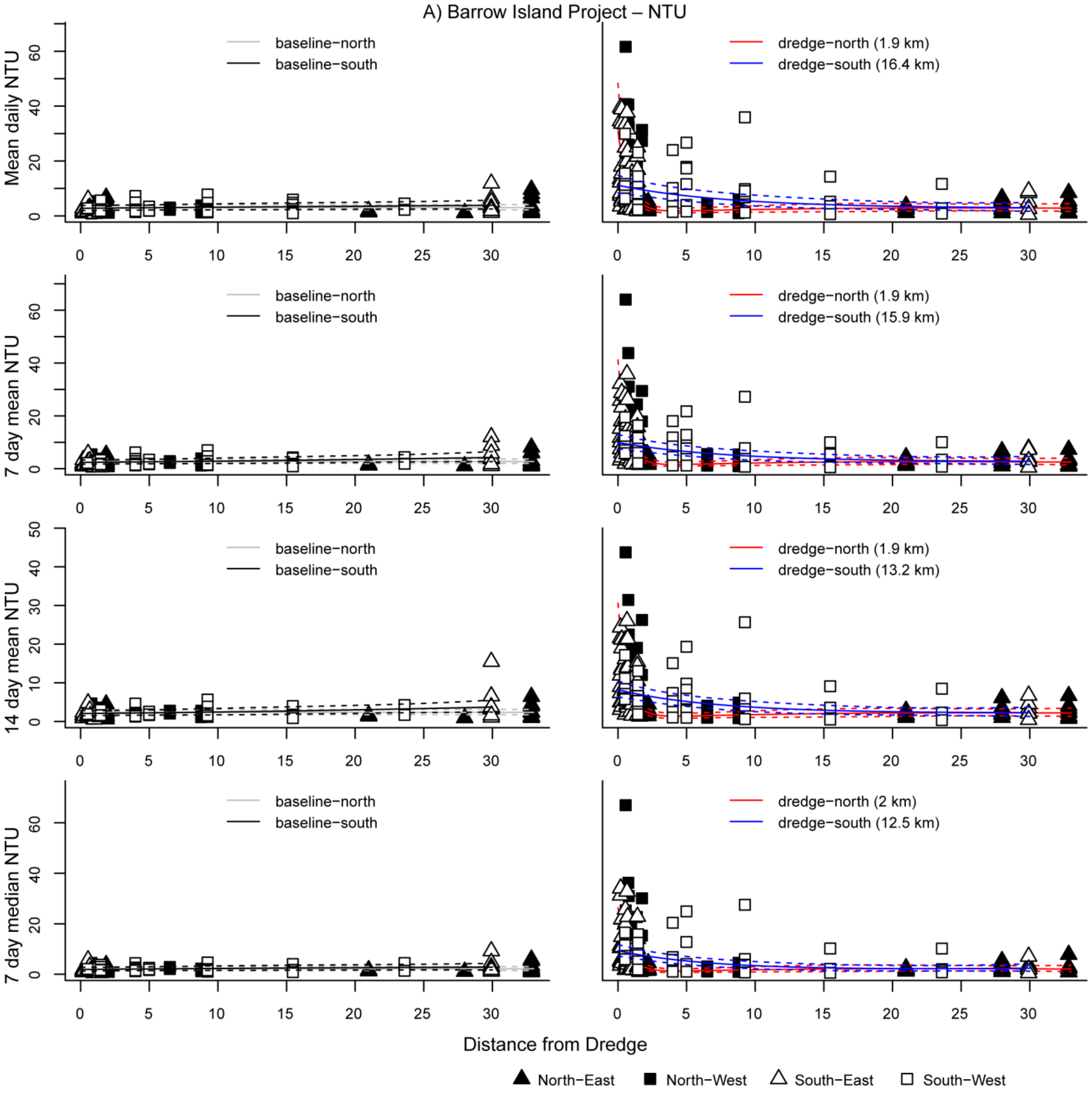

Figure S1.A (part 1). Distance decay relationships for turbidity (NTU) based water quality metrics at Barrow Island. Solid lines represent fitted best fit Generalised Additive Mixed Models (GAMM), with dashed lines indicating 95% confidence bounds for the fitted curves. Baseline and dredge periods were fitted as a two way interaction with distance from dredge, or as a three way interaction as appropriate (North/South or East/West of the location of the primary dredging activity, see methods for further details). Values in parentheses indicate the distance at which the fitted curve falls below the 80th percentile of the baseline value (i.e. the dredging effect becomes negligible). Data points represent quarterly 95th percentile values for each site and period (baseline or dredge).

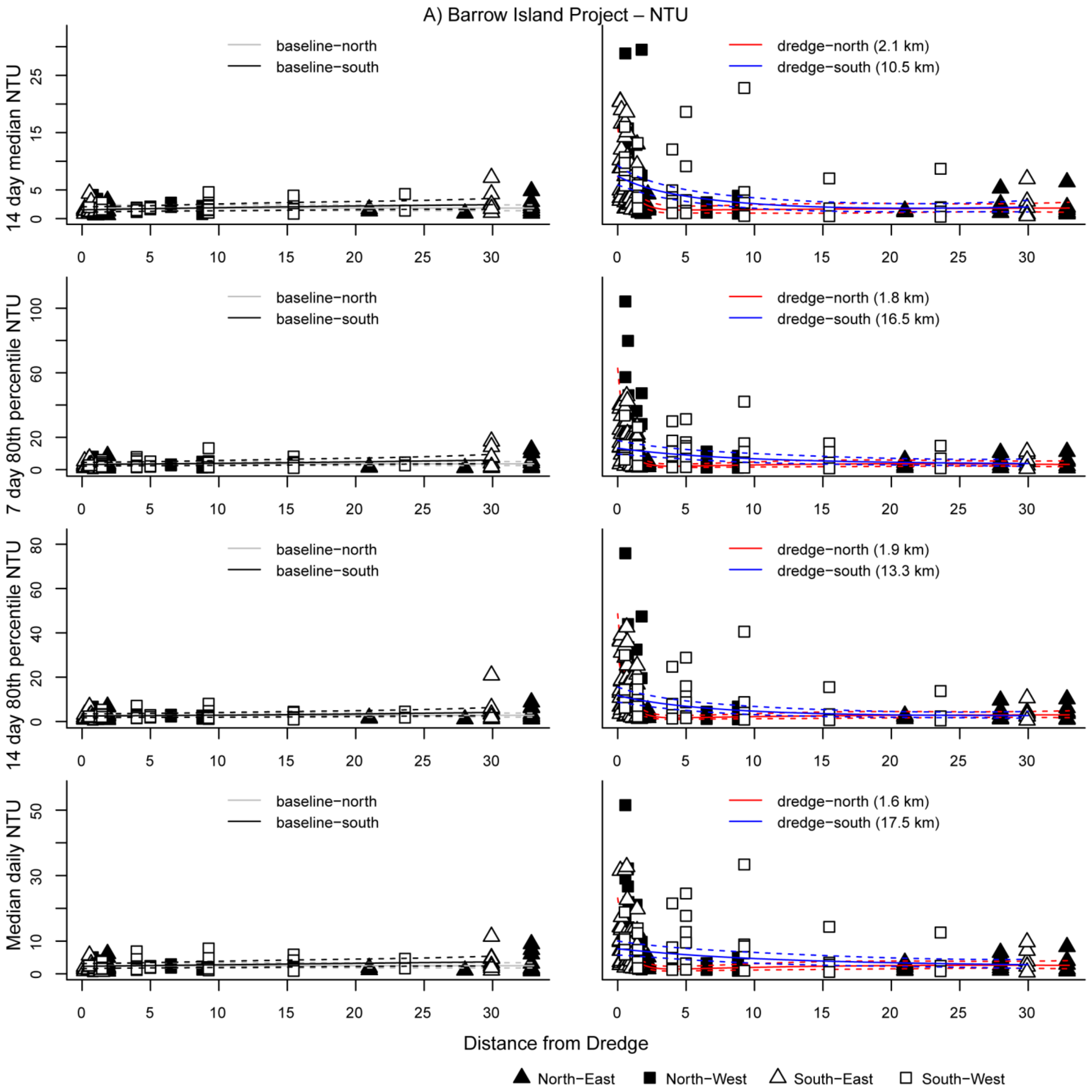

Figure S1.A (part 2). Distance decay relationships for turbidity (NTU) based water quality metrics at Barrow Island. Solid lines represent fitted best fit Generalised Additive Mixed Models (GAMM), with dashed lines indicating 95% confidence bounds for the fitted curves. Baseline and dredge periods were fitted as a two way interaction with distance from dredge, or as a three way interaction as appropriate (North/South or East/West of the location of the primary dredging activity, see methods for further details). Values in parentheses indicate the distance at which the fitted curve falls below the 80th percentile of the baseline value (i.e. the dredging effect becomes negligible). Data points represent quarterly 95th percentile values for each site and period (baseline or dredge).

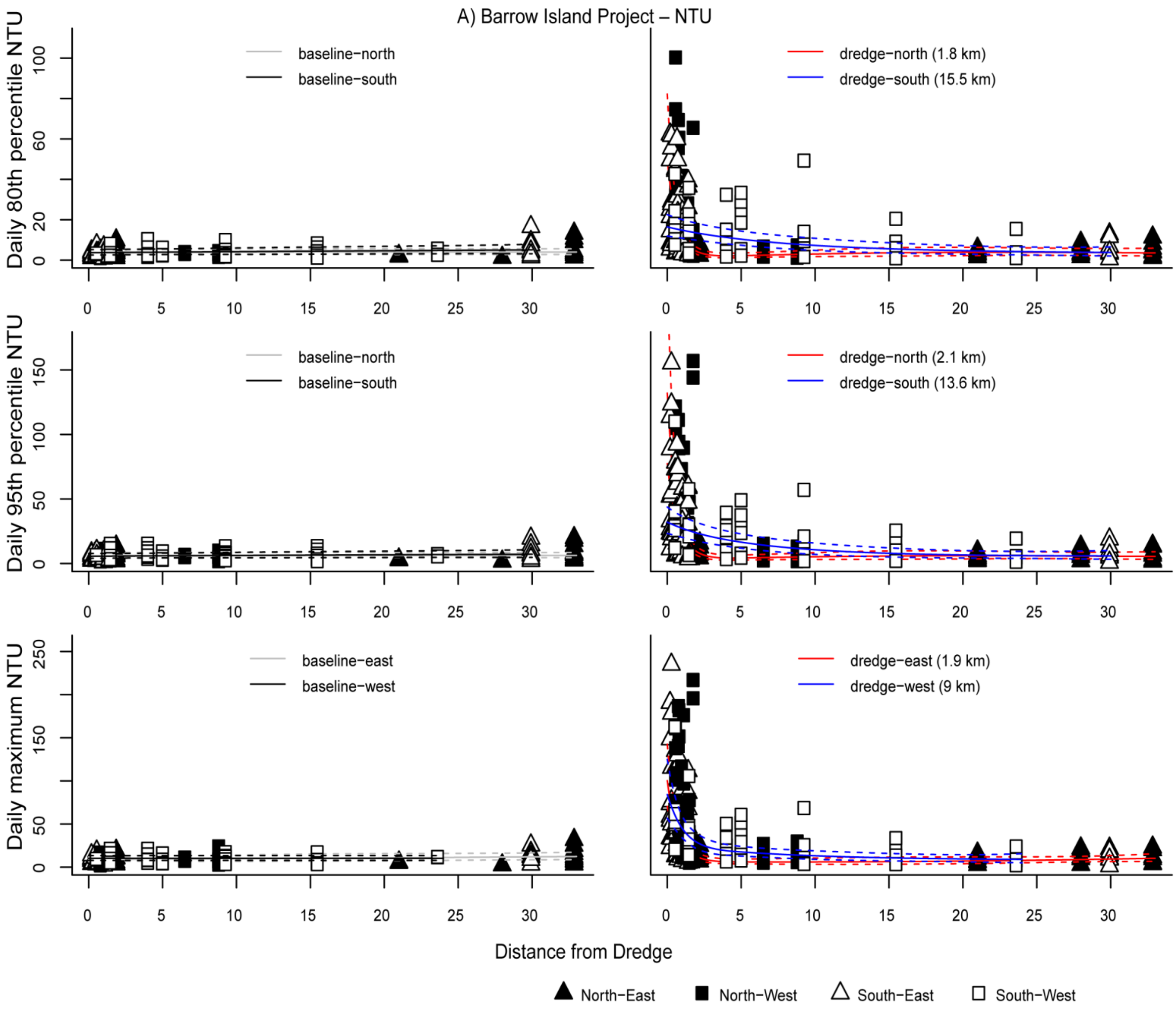

Figure S1.A (part 3). Distance decay relationships for turbidity (NTU) based water quality metrics at Barrow Island. Solid lines represent fitted best fit Generalised Additive Mixed Models (GAMM), with dashed lines indicating 95% confidence bounds for the fitted curves. Baseline and dredge periods were fitted as a two way interaction with distance from dredge, or as a three way interaction as appropriate (North/South or East/West of the location of the primary dredging activity, see methods for further details). Values in parentheses indicate the distance at which the fitted curve falls below the 80th percentile of the baseline value (i.e. the dredging effect becomes negligible). Data points represent quarterly 95th percentile values for each site and period (baseline or dredge).

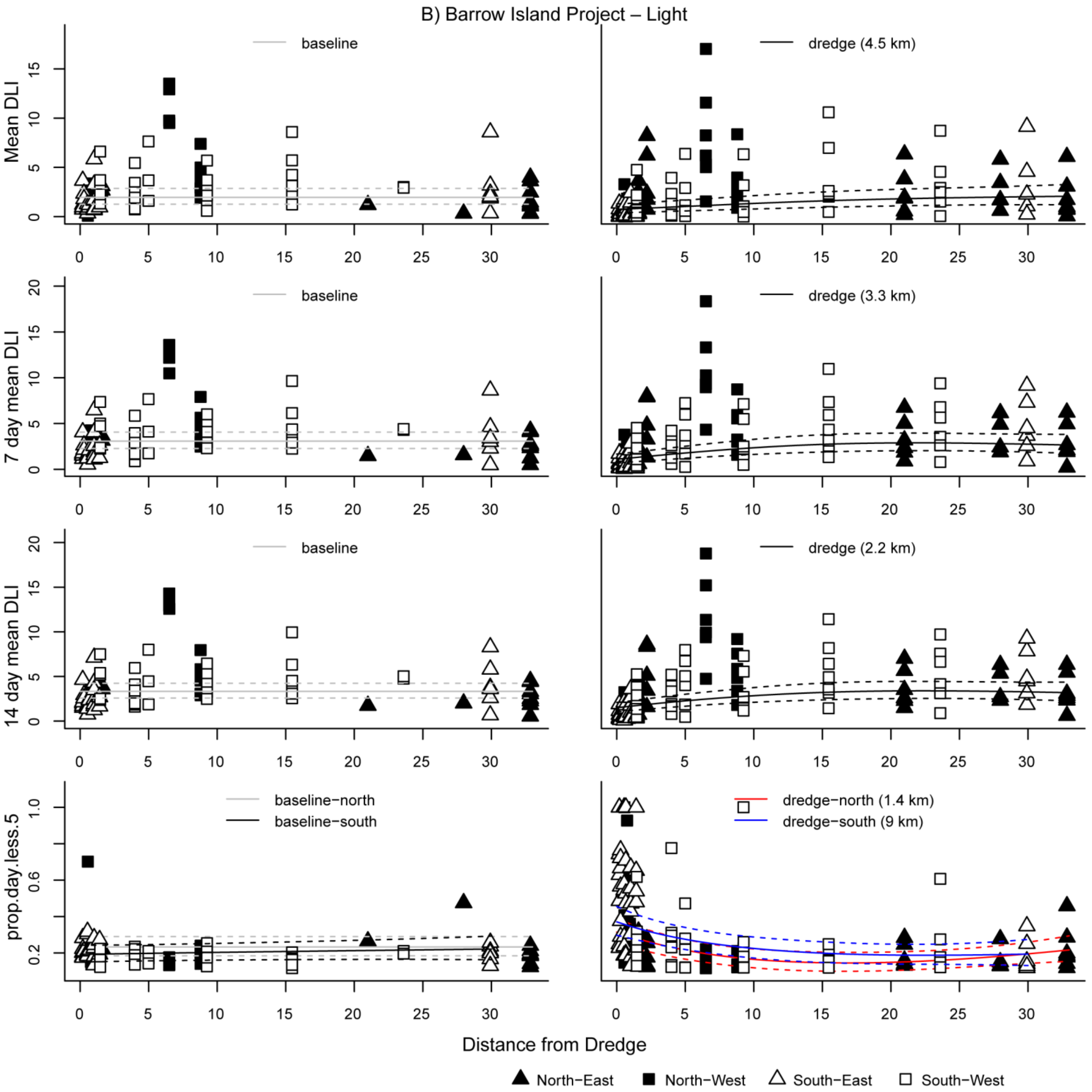

Figure S1.B (part 1). Distance decay relationships for light (PAR) based water quality metrics at Barrow Island. Solid lines represent fitted best fit Generalised Additive Mixed Models (GAMM), with dashed lines indicating 95% confidence bounds for the fitted curves. Baseline and dredge periods were fitted as a two way interaction with distance from dredge, or as a three way interaction as appropriate (North/South or East/West of the location of the primary dredging activity, see methods for further details). Values in parentheses indicate the distance at which the fitted curve falls below the 80th percentile of the baseline value (i.e. the dredging effect becomes negligible). Data points represent quarterly 5th percentile values for each site and period (baseline or dredge).

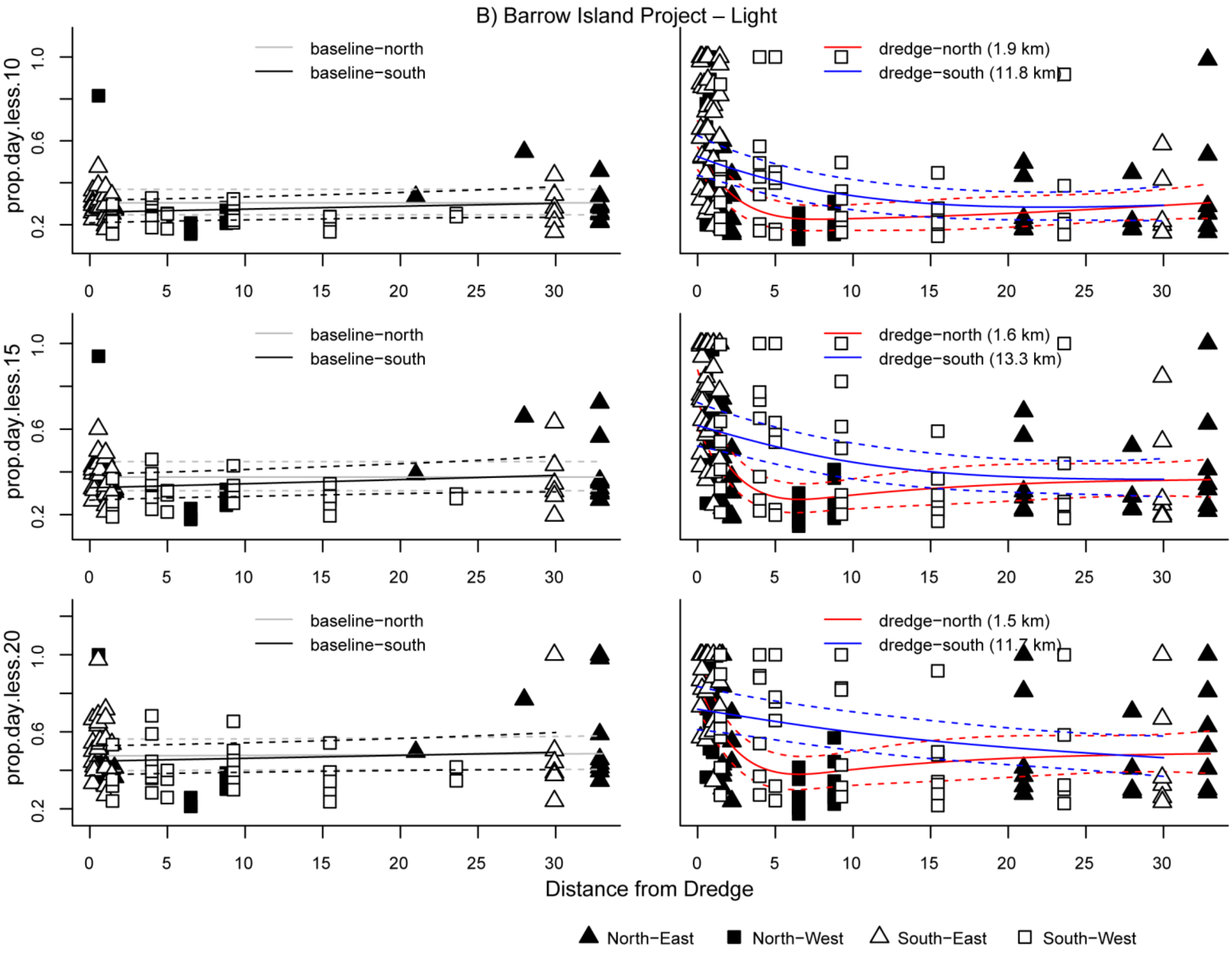

Figure S1.B (part 2). Distance decay relationships for light (PAR) based water quality metrics at Barrow Island. Solid lines represent fitted best fit Generalised Additive Mixed Models (GAMM), with dashed lines indicating 95% confidence bounds for the fitted curves. Baseline and dredge periods were fitted as a two way interaction with distance from dredge, or as a three way interaction as appropriate (North/South or East/West of the location of the primary dredging activity, see methods for further details). Values in parentheses indicate the distance at which the fitted curve falls below the 80th percentile of the baseline value (i.e. the dredging effect becomes negligible). Data points represent quarterly 5th percentile values for each site and period (baseline or dredge).

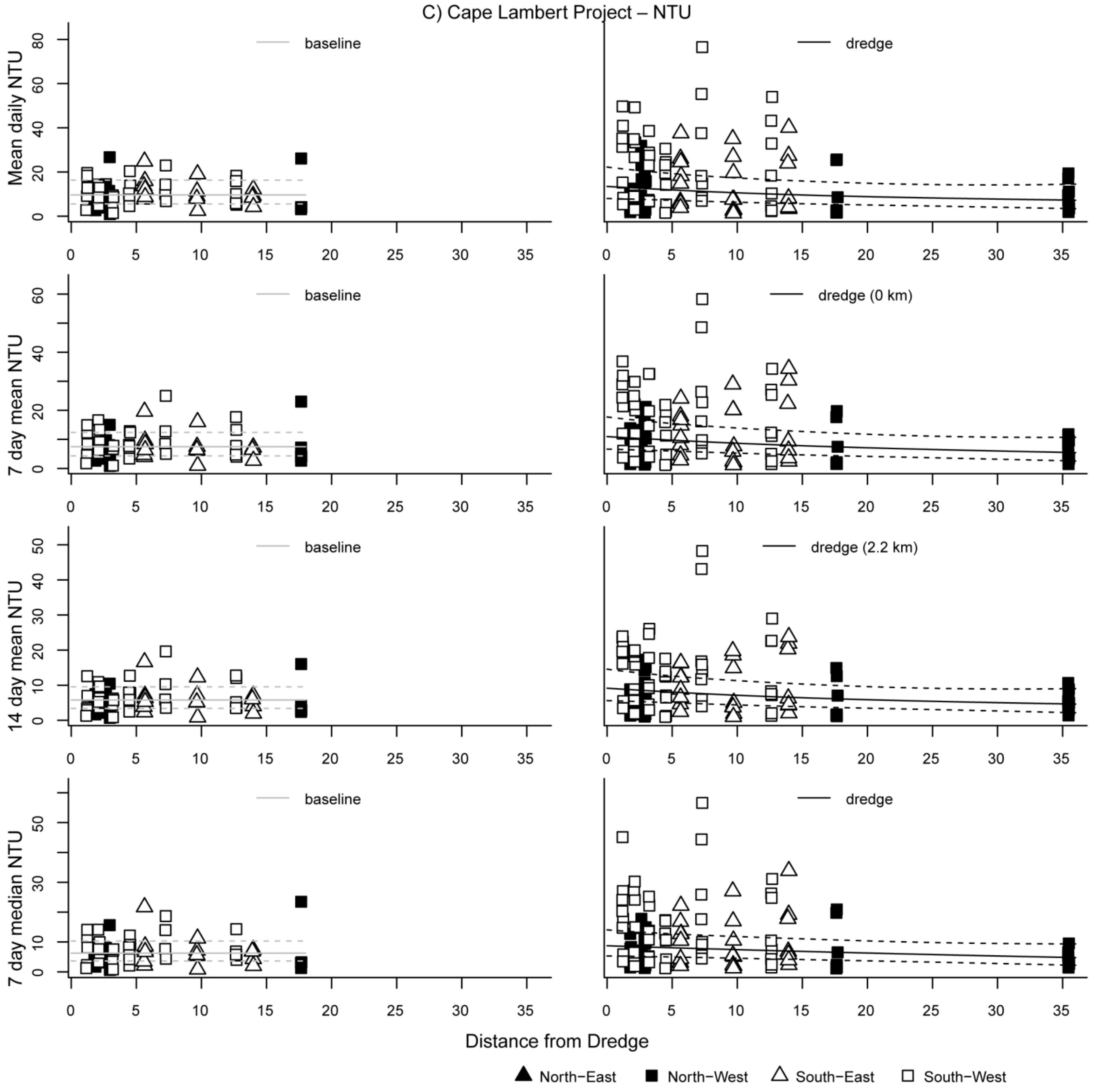

Figure S1.C (part 1). Distance decay relationships for turbidity (NTU) based water quality metrics at Cape Lambert. Solid lines represent fitted best fit Generalised Additive Mixed Models (GAMM), with dashed lines indicating 95% confidence bounds for the fitted curves. Baseline and dredge periods were fitted as a two way interaction with distance from dredge, or as a three way interaction as appropriate (North/South or East/West of the location of the primary dredging activity, see methods for further details). Values in parentheses indicate the distance at which the fitted curve falls below the 80th percentile of the baseline value (i.e. the dredging effect becomes negligible). Data points represent quarterly 95th percentile values for each site and period (baseline or dredge).

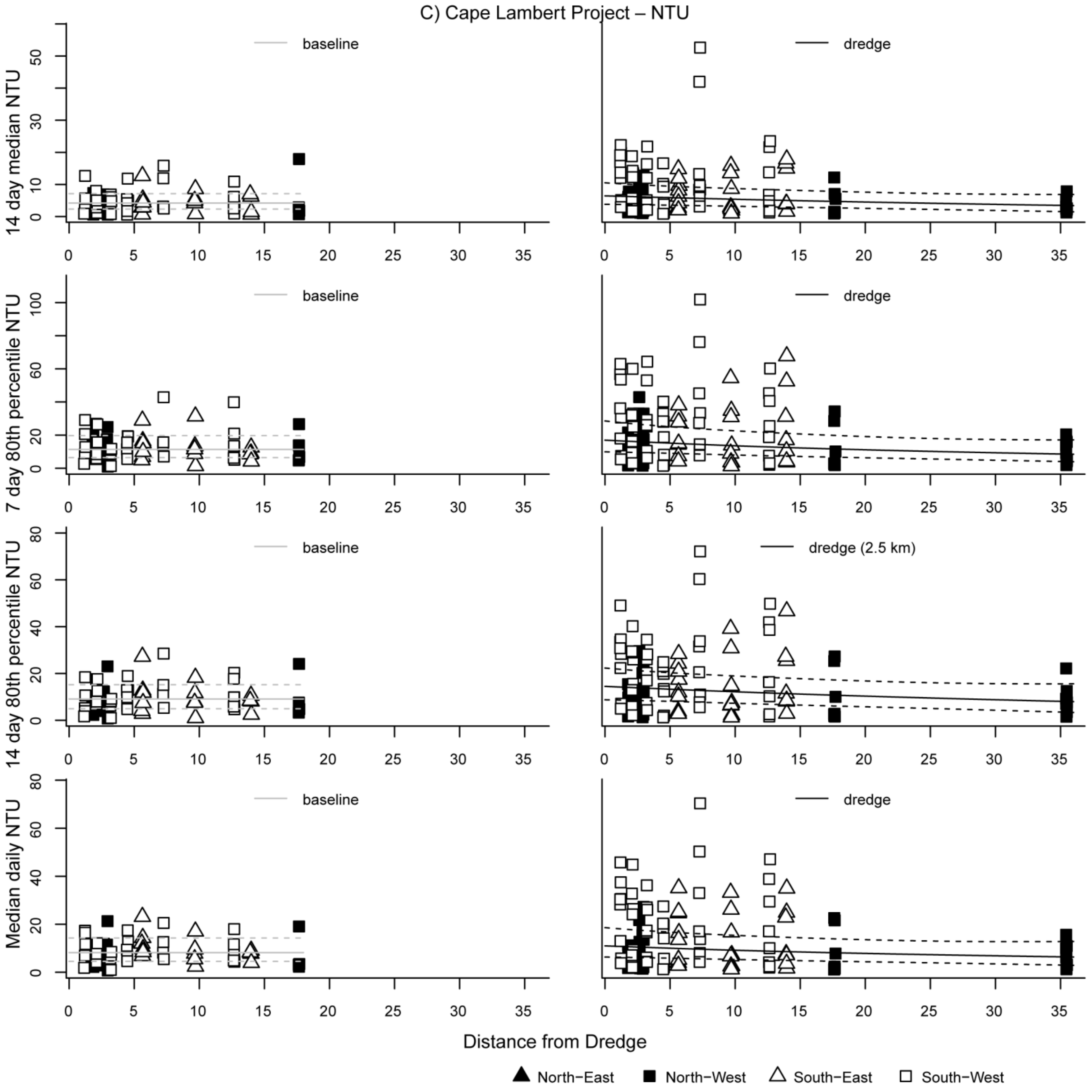

Figure S1.C (part 2). Distance decay relationships for turbidity (NTU) based water quality metrics at Cape Lambert. Solid lines represent fitted best fit Generalised Additive Mixed Models (GAMM), with dashed lines indicating 95% confidence bounds for the fitted curves. Baseline and dredge periods were fitted as a two way interaction with distance from dredge, or as a three way interaction as appropriate (North/South or East/West of the location of the primary dredging activity, see methods for further details). Values in parentheses indicate the distance at which the fitted curve falls below the 80th percentile of the baseline value (i.e. the dredging effect becomes negligible). Data points represent quarterly 95th percentile values for each site and period (baseline or dredge).

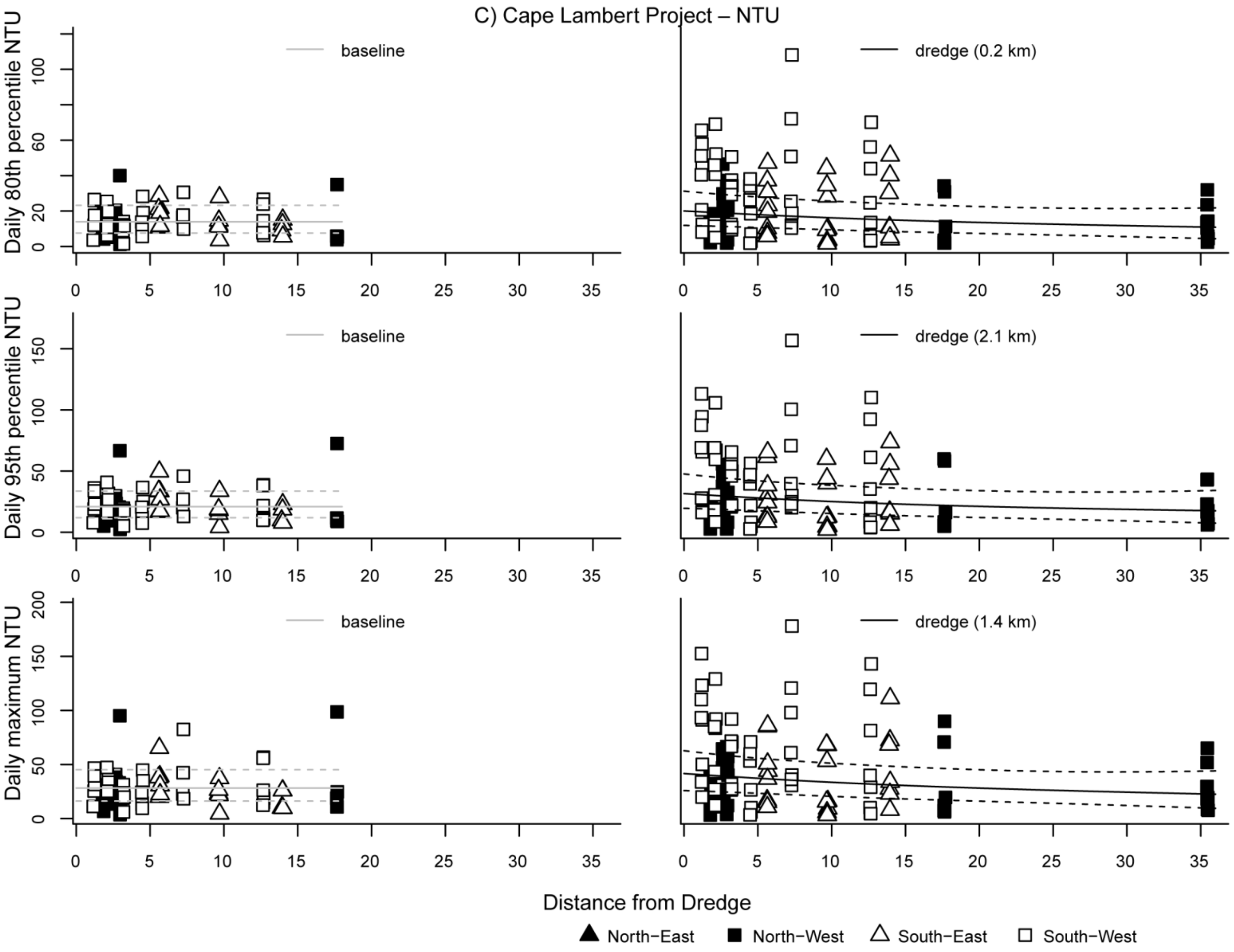

Figure S1.C (part 3). Distance decay relationships for turbidity (NTU) based water quality metrics at Cape Lambert. Solid lines represent fitted best fit Generalised Additive Mixed Models (GAMM), with dashed lines indicating 95% confidence bounds for the fitted curves. Baseline and dredge periods were fitted as a two way interaction with distance from dredge, or as a three way interaction as appropriate (North/South or East/West of the location of the primary dredging activity, see methods for further details). Values in parentheses indicate the distance at which the fitted curve falls below the 80th percentile of the baseline value (i.e. the dredging effect becomes negligible). Data points represent quarterly 95th percentile values for each site and period (baseline or dredge).

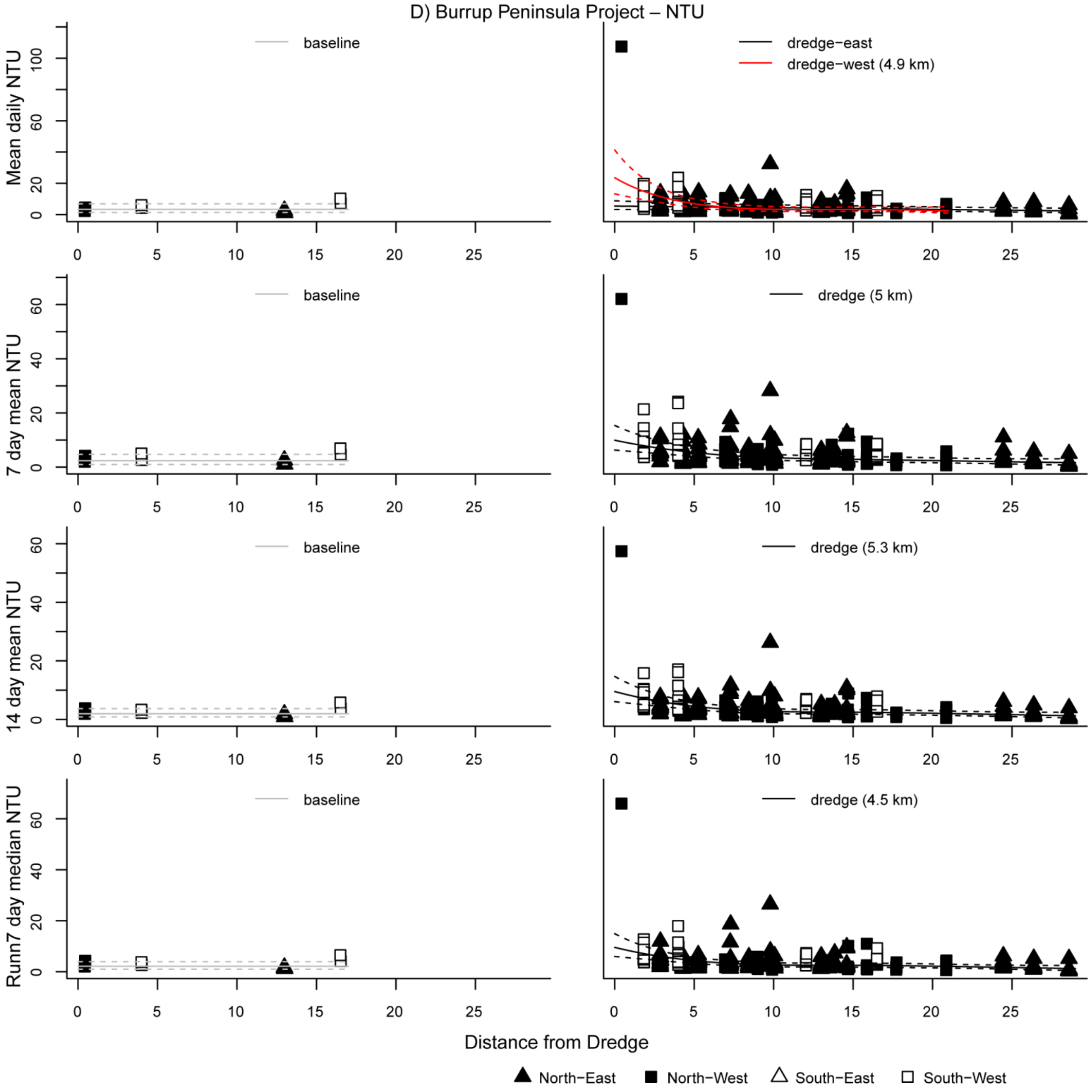

Figure S1.D (part 1). Distance decay relationships for turbidity (NTU) based water quality metrics at Burrup Peninsula. Solid lines represent fitted best fit Generalised Additive Mixed Models (GAMM), with dashed lines indicating 95% confidence bounds for the fitted curves. Baseline and dredge periods were fitted as a two way interaction with distance from dredge, or as a three way interaction as appropriate (North/South or East/West of the location of the primary dredging activity, see methods for further details). Values in parentheses indicate the distance at which the fitted curve falls below the 80th percentile of the baseline value (i.e. the dredging effect becomes negligible). Data points represent quarterly 95th percentile values for each site and period (baseline or dredge).

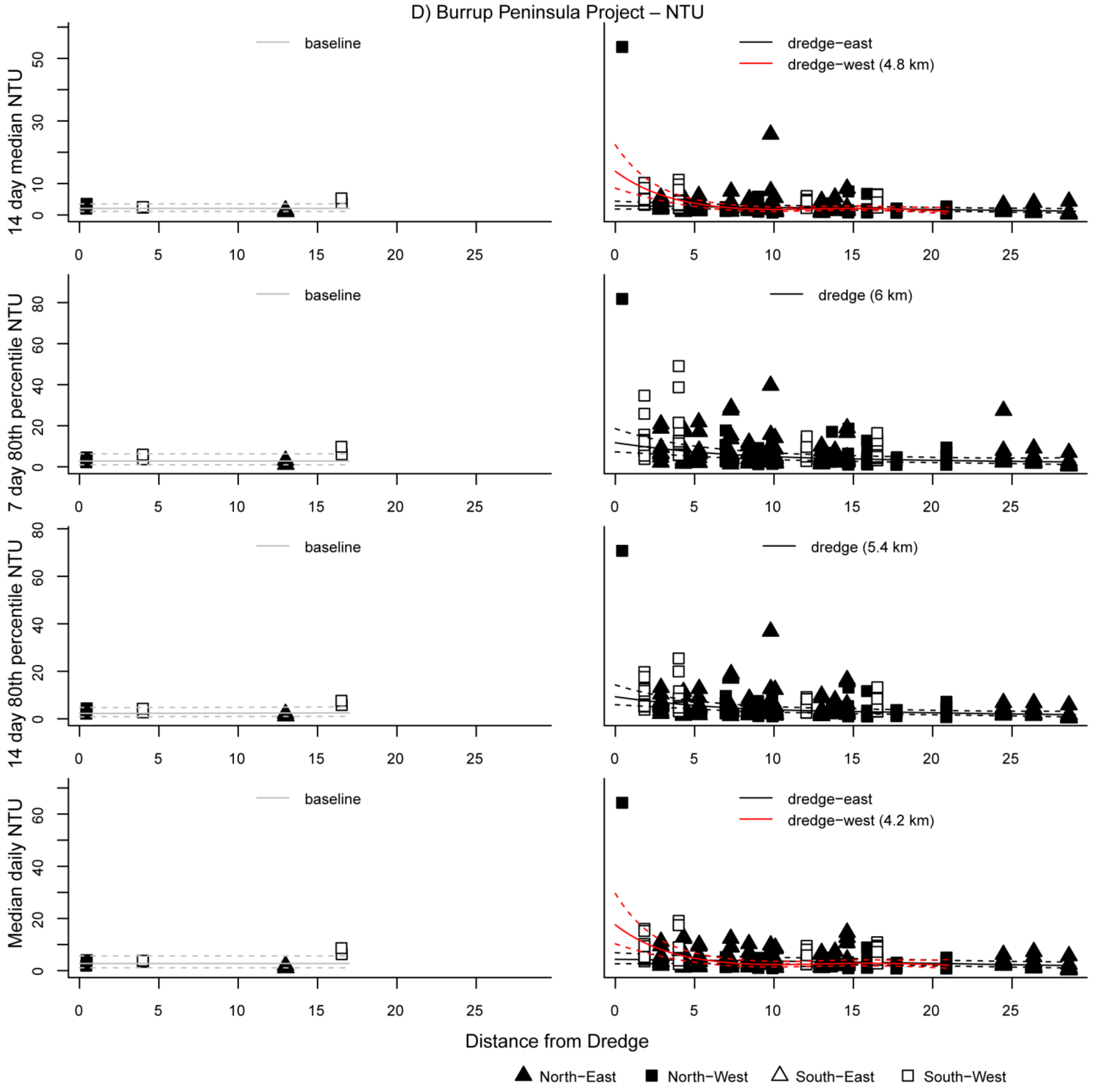

Figure S1.D (part 2). Distance decay relationships for turbidity (NTU) based water quality metrics at Burrup Peninsula. Solid lines represent fitted best fit Generalised Additive Mixed Models (GAMM), with dashed lines indicating 95% confidence bounds for the fitted curves. Baseline and dredge periods were fitted as a two way interaction with distance from dredge, or as a three way interaction as appropriate (North/South or East/West of the location of the primary dredging activity, see methods for further details). Values in parentheses indicate the distance at which the fitted curve falls below the 80th percentile of the baseline value (i.e. the dredging effect becomes negligible). Data points represent quarterly 95th percentile values for each site and period (baseline or dredge).

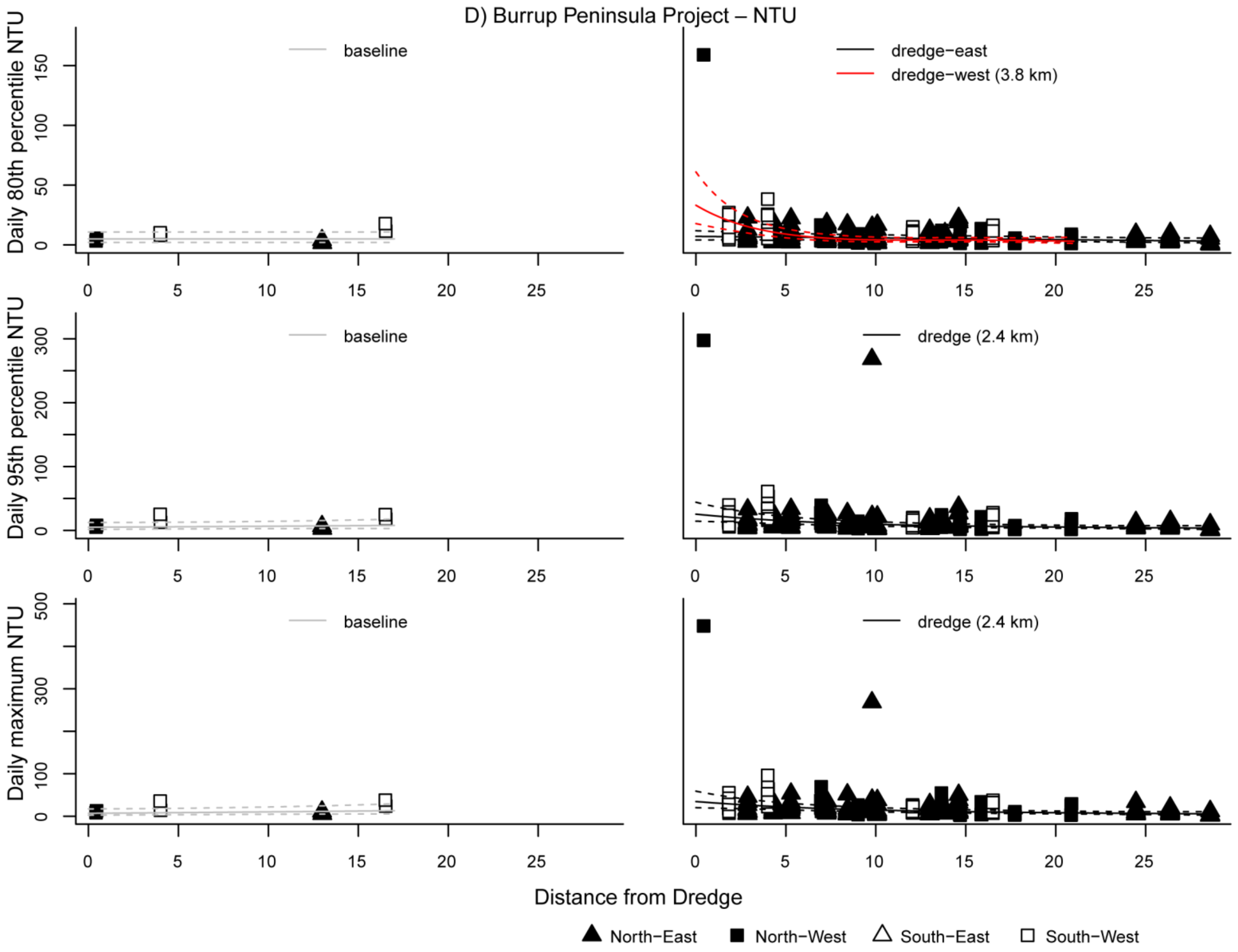

Figure S1.D (part 3). Distance decay relationships for turbidity (NTU) based water quality metrics at Burrup Peninsula. Solid lines represent fitted best fit Generalised Additive Mixed Models (GAMM), with dashed lines indicating 95% confidence bounds for the fitted curves. Baseline and dredge periods were fitted as a two way interaction with distance from dredge, or as a three way interaction as appropriate (North/South or East/West of the location of the primary dredging activity, see methods for further details). Values in parentheses indicate the distance at which the fitted curve falls below the 80th percentile of the baseline value (i.e. the dredging effect becomes negligible). Data points represent quarterly 95th percentile values for each site and period (baseline or dredge).
