# Supplementary material for: Spatial Patterns in Water Quality Changes during Dredging in Tropical Environments
Source: PLoS One. 2015 Dec 2;10(12):e0143309. doi: 10.1371/journal.pone.0143309 (PMC4667927; doi:10.1371/journal.pone.0143309)
Supplement: S1 Table — Max, 99th, 95th, 80th percentiles, median and mean NTU values over 1 h, 1 d, 14 d, and 21 d running average period at all sites during the baseline period or for during the duration of the dredging program. (PDF) [file pone.0143309.s002.pdf]

S1 Table - Detailed summary data

Table S1.A. Max, 99th, 95th, 80th percentiles, median and mean NTU values over 1 h, 1 d, 14 d, and 21 d running average period at all sites during the baseline period ("B", before dredging) or for during the duration of the dredging program ("D"). The ratio of dredging/baseline is also shown ("∆"). For each site the distance from dredging activities (Distn), the site depth (Depth) and the number of sampling days during baseline (Base) and dredging (Dred) are shown. NTU values approximate total suspendid solid concentrations with a linear conversion factor of between 1.1 and 2.1.

Site	Details	Stat.	B1H	D1H	∆1H	B1D	D1D	∆1D	B1W	D1W	∆1W	B2W	D2W	∆2W	B3W	D3W	∆3W
**Barrow**	**Island**	Max	40.1	82.9	2.1	19	16.7	0.9	8.3	8.4	1	6.7	4.4	0.7	6.1	3.5	0.6
Name:	**AHC**	99th	12.2	10.9	0.9	10.9	8.5	0.8	8	7.3	0.9	6.4	4	0.6	6	3.5	0.6
Distn:	32.8	95th	5.1	4.8	0.9	4.7	4.4	0.9	5.4	3.3	0.6	5	2.7	0.5	5.6	3.1	0.6
Depth:	6.9	80th	1.8	1.5	0.8	1.7	1.6	0.9	1.9	1.8	0.9	2.1	1.8	0.8	2.1	1.6	0.8
Base.:	668	Med.	0.9	0.7	0.8	0.9	0.7	0.8	0.9	0.9	1	1	0.9	0.9	1	1.1	1.1
Dred.:	500	Mean	1.6	1.3	0.8	1.5	1.2	0.8	1.6	1.2	0.8	1.6	1.1	0.7	1.6	1.2	0.7
**Barrow**	**Island**	Max	68.6	68.2	1	15	17.8	1.2	4.3	6.7	1.6	2.8	5.6	2	2.7	4.7	1.7
Name:	**ANT**	99th	4.6	9.3	2	4.2	8.1	1.9	4	6.4	1.6	2.8	5.5	2	2.5	4.7	1.9
Distn:	8.8	95th	2.5	3.8	1.5	2.4	3.7	1.6	2.4	4.7	1.9	2.1	4.1	1.9	2	4.3	2.1
Depth:	3.9	80th	1.7	1.8	1	1.9	2	1.1	1.7	2	1.2	1.6	2.1	1.3	1.6	2.4	1.5
Base.:	756	Med.	1.1	0.9	0.8	1.2	0.9	0.8	1.2	1	0.8	1.3	1	0.8	1.3	1.1	0.8
Dred.:	376	Mean	1.3	1.4	1.1	1.4	1.4	1	1.4	1.5	1.1	1.3	1.5	1.2	1.3	1.6	1.2
**Barrow**	**Island**	Max	31	72.6	2.3	12	33.3	2.8	5.1	9.1	1.8	4.2	6.5	1.6	4.1	4.6	1.1
Name:	**BAT**	99th	6.9	18.1	2.6	4.9	15.4	3.2	4.3	8	1.9	3.7	4.9	1.3	2.7	3.3	1.2
Distn:	15.5	95th	3.7	5.1	1.4	3.8	5.1	1.3	3.4	5.7	1.7	2.8	3.7	1.3	2.6	2.8	1.1
Depth:	3.7	80th	1.8	1.4	0.8	1.9	1.6	0.8	1.8	1.9	1.1	1.8	2	1.1	1.8	1.8	1
Base.:	611	Med.	1	0.6	0.6	1	0.7	0.6	1.1	0.7	0.6	1.1	0.7	0.6	1.1	0.7	0.6
Dred.:	416	Mean	1.4	1.5	1.1	1.4	1.5	1.1	1.4	1.5	1.1	1.3	1.3	1	1.3	1.1	0.9
**Barrow**	**Island**	Max	68.6	67.8	1	15	21	1.4	4.3	6.4	1.5	2.8	4.1	1.5	2.7	3	1.1
Name:	**DIW**	99th	4.8	7.9	1.6	4.1	6.3	1.5	4	5.9	1.5	2.8	4	1.5	2.5	3	1.2
Distn:	6.5	95th	2.9	3	1	2.4	3.2	1.3	2.5	3.5	1.4	2.1	3.2	1.5	2.1	2.8	1.3
Depth:	1.9	80th	1.9	1.8	0.9	1.9	1.8	1	1.9	2	1.1	1.8	1.8	1	1.7	1.7	1
Base.:	222	Med.	1.1	0.9	0.8	1.2	0.9	0.8	1.3	1	0.8	1.3	1	0.8	1.2	1	0.8
Dred.:	390	Mean	1.4	1.3	0.9	1.4	1.3	0.9	1.4	1.3	1	1.4	1.3	1	1.3	1.2	0.9
**Barrow**	**Island**	Max	38.1	82.1	2.2	12.4	56.3	4.6	4.8	31.3	6.6	2.9	29.1	10	2.5	20.6	8.3
Name:	**DUG**	99th	8	38.1	4.7	5.9	38.3	6.5	3.7	28.2	7.5	2.3	26.5	11.3	2.3	20.5	8.7
Distn:	9.2	95th	3.8	14	3.7	3.2	13.9	4.3	2.6	13.6	5.2	2.1	9.6	4.6	2.1	7.2	3.4
Depth:	6	80th	1.7	2.7	1.6	1.6	3.1	1.9	1.6	4.3	2.6	1.7	4.2	2.5	1.7	3.9	2.3
Base.:	786	Med.	1	1	1	1.1	1.1	1	1.1	1.4	1.3	1.2	2.1	1.7	1.3	2.5	1.9
Dred.:	464	Mean	1.4	3	2.2	1.3	3	2.3	1.3	3.2	2.5	1.3	3.2	2.6	1.3	3.1	2.4
**Barrow**	**Island**	Max	40.1	35.4	0.9	19	19.2	1	8.3	4.7	0.6	6.7	3.1	0.5	6.1	2.8	0.5
Name:	**ELS**	99th	12.1	6.3	0.5	10.9	5.3	0.5	8	4.6	0.6	6.4	3	0.5	6	2.7	0.4
Distn:	21	95th	5	2.7	0.5	4.7	2.7	0.6	5.2	2.2	0.4	5	2.5	0.5	5.3	2.4	0.5
Depth:	7	80th	1.8	1.2	0.7	1.8	1.2	0.7	1.8	1.4	0.8	1.7	1.6	0.9	2.1	1.5	0.7
Base.:	133	Med.	1.1	0.8	0.8	1.1	0.9	0.8	1.2	1	0.8	1.3	1	0.8	1.3	1.2	0.9
Dred.:	361	Mean	1.6	1	0.6	1.6	1.1	0.7	1.6	1.1	0.6	1.7	1.1	0.7	1.7	1.2	0.7
**Barrow**	**Island**	Max	18.3	180.5	9.9	8.5	66.7	7.8	3.7	34.9	9.3	1.9	25.4	13.3	1.8	21.3	12.2
Name:	**LNG0**	99th	5.6	64	11.4	4.9	42.5	8.7	3.5	31.9	9.1	1.9	24.2	13	1.7	20.6	12.4
Distn:	0.2	95th	2.5	24.6	9.8	2.3	21.7	9.3	2.2	18.6	8.5	1.7	19.6	11.3	1.6	18.2	11.3
Depth:	8.6	80th	1.4	6.4	4.7	1.3	9	6.8	1.5	10.2	7	1.5	10.8	7.3	1.5	11.3	7.6
Base.:	457	Med.	1	2.4	2.5	1.1	3.1	2.9	1.1	3.8	3.3	1.2	4.1	3.5	1.2	4.5	3.7
Dred.:	479	Mean	1.2	6.1	5.3	1.2	6.2	5.2	1.2	6.4	5.2	1.2	6.5	5.5	1.2	6.6	5.6
**Barrow**	**Island**	Max	25.6	224.3	8.8	13.5	67.3	5	6.9	32.6	4.7	5.1	23.3	4.6	4	18.9	4.7
Name:	**LNG1**	99th	6.3	51.4	8.2	6.5	35.6	5.4	6	27.3	4.6	4.9	21.1	4.3	3.9	18.4	4.7
Distn:	0.5	95th	2.9	20.9	7.3	2.4	18	7.5	2.6	14.6	5.7	2.7	15	5.7	3.6	15.2	4.2
Depth:	8.9	80th	1.4	5.6	4.1	1.4	7.1	5.1	1.3	8.1	6	1.4	8.1	5.8	1.3	7.8	5.8
Base.:	629	Med.	1	2.2	2.2	1	2.7	2.6	1.1	3.8	3.5	1.1	4.3	3.9	1.1	4.3	3.8
Dred.:	481	Mean	1.2	5.2	4.2	1.3	5.2	4.2	1.3	5.3	4.3	1.3	5.4	4.2	1.3	5.4	4
**Barrow**	**Island**	Max	21.4	128.7	6	8.5	36.7	4.3	2.7	14.3	5.3	2.4	11.3	4.8	2.1	9.5	4.6
Name:	**LNG2**	99th	5.2	31.4	6	3.8	21	5.5	2.6	13.4	5.2	2.3	10.2	4.4	2.1	8.9	4.3
Distn:	1	95th	2.4	13.3	5.7	2.3	11.5	5	1.9	9.8	5.1	1.6	8.4	5.1	1.8	7.6	4.1
Depth:	6.6	80th	1.3	4.5	3.6	1.3	5.4	4	1.4	5.8	4.2	1.4	5.4	4	1.3	5.5	4.2
Base.:	632	Med.	0.9	1.9	2.2	1	2.2	2.3	1.1	2.7	2.5	1.1	2.7	2.5	1.1	3.2	2.9
Dred.:	510	Mean	1	3.8	3.6	1.1	3.7	3.5	1.1	3.7	3.4	1.1	3.6	3.3	1.1	3.6	3.3
**Barrow**	**Island**	Max	25.7	104.4	4.1	17.9	60.6	3.4	3.2	23.7	7.5	2.2	18.4	8.2	2.3	13.4	5.7
Name:	**LNG3**	99th	8.6	32.4	3.8	5.2	28.3	5.4	3.1	19.3	6.2	2.2	16.4	7.3	2.3	13.3	5.7
Distn:	4	95th	3.2	12.8	4	2.5	12.4	5	2.4	11.9	5	2	9.5	4.6	2	9.5	4.7
Depth:	6.2	80th	1.6	3.2	2	1.6	3.7	2.3	1.5	4.6	3	1.7	4.1	2.4	1.7	4.3	2.5
Base.:	655	Med.	1.1	1.5	1.3	1.2	1.5	1.3	1.2	1.8	1.5	1.2	2	1.7	1.2	2.4	1.9
Dred.:	493	Mean	1.4	3.3	2.3	1.3	3.3	2.5	1.3	3.3	2.6	1.2	3.1	2.5	1.2	3.1	2.5
**Barrow**	**Island**	Max	18.3	212.6	11.6	8.5	75.2	8.8	3.7	34.3	9.2	1.9	22.4	11.7	1.8	16.5	9.4
Name:	**LNGA**	99th	5.6	71.6	12.8	5	50.6	10.1	3.5	29.8	8.5	1.9	22	11.7	1.7	16.5	9.9

Table S1.A. Max, 99th, 95th, 80th percentiles, median and mean NTU values over 1 h, 1 d, 14 d, and 21 d running average period at all sites during the baseline period ("B", before dredging) or for during the duration of the dredging program ("D"). The ratio of dredging/baseline is also shown ("Δ"). For each site the distance from dredging activities (Distn), the site depth (Depth) and the number of sampling days during baseline (Base) and dredging (Dred) are shown. NTU values approximate total suspendid solid concentrations with a linear conversion factor of between 1.1 and 2.1.

Site	Details	Stat.	B1H	D1H	Δ1H	B1D	D1D	Δ1D	B1W	D1W	Δ1W	B2W	D2W	Δ2W	B3W	D3W	Δ3W
Distn:	0.3	95th	2.5	24.7	9.8	2.3	21.9	9.6	2.2	19.7	8.9	1.7	18.8	10.9	1.6	15.4	9.5
Depth:	11.1	80th	1.5	6.2	4.2	1.5	8.7	6	1.6	10	6.4	1.5	10.9	7.3	1.5	10.7	7.2
Base.:	117	Med.	1.1	2.6	2.3	1.2	3.3	2.7	1.3	3.8	2.9	1.4	3.9	2.9	1.4	3.9	2.8
Dred.:	488	Mean	1.3	6.4	5.1	1.3	6.5	5	1.4	6.5	4.6	1.4	6.2	4.6	1.4	6	4.4
**Barrow**	**Island**	Max	18.3	335.6	18.4	8.5	89.1	10.5	3.7	42	11.2	2.9	27.8	9.5	2.9	22.1	7.7
Name:	**LNGB**	99th	5.6	52.2	9.3	5	40	7.9	3.5	35.4	10.1	2.9	26	8.9	2.8	21.6	7.6
Distn:	0.7	95th	3.1	20.9	6.7	3	18.7	6.3	2.9	14.6	5.1	2.8	11.5	4.2	2.7	10.4	3.8
Depth:	10.2	80th	2.1	5.8	2.7	2.3	7.4	3.2	2.5	9.1	3.7	2.5	7.7	3.1	2.5	6.9	2.8
Base.:	94	Med.	1.1	2.2	2.1	1.1	2.8	2.5	1.3	3.7	2.9	1.3	4.3	3.3	1.4	5.3	3.8
Dred.:	501	Mean	1.4	5.4	3.9	1.4	5.5	3.8	1.5	5.6	3.7	1.5	5.6	3.7	1.5	5.5	3.5
**Barrow**	**Island**	Max	18.3	173.2	9.5	8.5	63.3	7.4	3.7	25.1	6.7	1.9	18.6	9.7	1.8	13.9	7.9
Name:	**LNGC**	99th	5.3	47.4	8.9	4.8	33.5	7	3.5	20.8	6	1.9	15.4	8.3	1.7	13	7.9
Distn:	1.4	95th	2.3	18.1	7.7	2.2	18.3	8.3	2.2	14.1	6.4	1.7	12.2	7.1	1.6	11	6.9
Depth:	10.7	80th	1.4	5	3.6	1.4	6.3	4.6	1.4	7.3	5.1	1.4	7.1	5.1	1.4	6.8	4.8
Base.:	249	Med.	1.1	2	1.8	1.1	2.3	2.1	1.2	3.1	2.6	1.2	4	3.3	1.2	4.1	3.3
Dred.:	486	Mean	1.2	4.7	4	1.2	4.8	4	1.2	4.8	3.9	1.2	4.8	4	1.2	4.9	4.1
**Barrow**	**Island**	Max	28.7	34.6	1.2	13	21.6	1.7	3.8	6.3	1.7	2.9	4.2	1.4	2.7	3.4	1.3
Name:	**LOW**	99th	8.4	10.5	1.2	4.8	7.9	1.6	3.2	6.1	1.9	2.8	4.1	1.4	2.6	3.4	1.3
Distn:	1.9	95th	3.6	4.5	1.3	2.9	3.8	1.3	2.5	3.6	1.4	2.4	3.6	1.5	2.4	3.2	1.4
Depth:	2.9	80th	1.7	1.8	1.1	1.7	2	1.2	1.6	2	1.2	1.7	1.9	1.1	1.7	1.8	1.1
Base.:	692	Med.	1	0.8	0.8	1	0.9	0.9	1.2	1.1	0.9	1.3	1.1	0.9	1.3	1.2	0.9
Dred.:	459	Mean	1.4	1.4	1	1.3	1.4	1.1	1.3	1.4	1.1	1.3	1.4	1.1	1.4	1.4	1
**Barrow**	**Island**	Max	28.7	27.2	0.9	13	16.4	1.3	3.2	4.7	1.5	2.9	3.7	1.3	2.7	2.9	1.1
Name:	**LOW1**	99th	7.8	6.5	0.8	4.1	5.4	1.3	2.9	4.3	1.5	2.8	3.6	1.3	2.6	2.9	1.1
Distn:	1.6	95th	3.3	3	0.9	2.7	3	1.1	2.3	2.5	1.1	2.3	2.6	1.1	2.3	2.8	1.2
Depth:	6.9	80th	1.6	1.7	1.1	1.6	1.7	1.1	1.5	1.7	1.1	1.5	1.8	1.2	1.6	1.9	1.2
Base.:	226	Med.	1	1.1	1	1.1	1.1	1	1.2	1.3	1	1.3	1.3	1	1.2	1.3	1
Dred.:	449	Mean	1.4	1.3	1	1.3	1.3	1	1.3	1.4	1.1	1.3	1.4	1.1	1.3	1.5	1.1
**Barrow**	**Island**	Max	28.7	34.6	1.2	13	18.4	1.4	3.7	5.8	1.5	2.9	4.9	1.7	2.7	3.8	1.4
Name:	**LOW3**	99th	8.6	8.8	1	4.8	6.8	1.4	3.2	5.1	1.6	2.8	4.6	1.7	2.6	3.6	1.3
Distn:	2.2	95th	3.6	4.1	1.1	2.9	3.8	1.3	2.4	3.5	1.5	2.4	2.8	1.2	2.4	2.6	1.1
Depth:	4.5	80th	1.7	1.8	1.1	1.7	1.9	1.1	1.6	2	1.2	1.7	2	1.2	1.7	1.9	1.1
Base.:	2	Med.	1	1.1	1.1	1.1	1.2	1.1	1.3	1.3	1	1.3	1.2	0.9	1.3	1.2	0.9
Dred.:	460	Mean	1.4	1.5	1.1	1.3	1.5	1.1	1.3	1.5	1.1	1.3	1.4	1	1.4	1.4	1
**Barrow**	**Island**	Max	19.9	234.3	11.8	10.9	90.9	8.3	3.2	32.5	10	2.5	20	8	2.4	17.2	7.3
Name:	**MOF1**	99th	6.7	56.6	8.4	4.2	36.8	8.8	2.9	19.3	6.6	2.4	16.5	6.9	2.3	15.3	6.6
Distn:	0.8	95th	2.9	18.6	6.4	2.8	15.9	5.7	2.5	14.3	5.7	2.4	14.6	6.2	2.1	13.9	6.6
Depth:	6.2	80th	1.7	5.2	3	1.6	6.7	4.3	1.7	7.8	4.6	1.7	8.9	5.3	1.7	11	6.5
Base.:	678	Med.	1.1	1.9	1.8	1.1	2.5	2.3	1.2	2.7	2.3	1.3	3.1	2.5	1.3	4.2	3.2
Dred.:	505	Mean	1.3	4.9	3.8	1.2	4.9	3.9	1.3	4.7	3.7	1.3	5.2	4	1.3	5.9	4.6
**Barrow**	**Island**	Max	29.5	106.1	3.6	12.1	51.8	4.3	4.5	12.8	2.9	3.6	4.1	1.1	3.3	3.2	1
Name:	**MOF3**	99th	7.4	29.5	4	6	23.6	3.9	4.1	12.4	3	3.2	4	1.3	3	3.2	1.1
Distn:	1.5	95th	3.4	12.2	3.6	3.5	12.3	3.5	3.2	7.6	2.4	3	3.9	1.3	2.9	3.2	1.1
Depth:	4.8	80th	1.6	3.5	2.1	2	4.2	2.1	2.6	4.9	1.8	2.7	2.4	0.9	2.8	2.3	0.8
Base.:	657	Med.	1	1.5	1.5	1.1	1.8	1.6	1.3	2.1	1.6	1.3	1.9	1.5	1.3	1.9	1.5
Dred.:	487	Mean	1.3	3.2	2.4	1.5	3.4	2.3	1.6	3	1.9	1.6	2	1.2	1.7	2	1.2
**Barrow**	**Island**	Max	19.9	433.9	21.8	10.9	178.8	16.3	3.3	75.8	22.7	2.5	47.4	18.9	2.4	33.7	14.2
Name:	**MOFA**	99th	6.7	90.1	13.4	4.2	72.4	17.1	3	65	21.5	2.4	44.4	18.4	2.3	33.7	14.5
Distn:	0.6	95th	3	28.3	9.6	2.8	26.8	9.5	2.5	21.7	8.8	2.3	26.5	11.3	2.1	27.5	13
Depth:	4.9	80th	1.9	6	3.1	1.9	6.9	3.7	1.9	9.1	4.8	1.9	8.4	4.4	1.9	7.3	4
Base.:	144	Med.	1.3	2.3	1.9	1.3	2.9	2.3	1.4	3.2	2.3	1.4	3.3	2.3	1.4	3.4	2.5
Dred.:	471	Mean	1.5	7	4.9	1.4	7	5	1.4	6.8	4.8	1.4	6.4	4.5	1.4	6.1	4.3
**Barrow**	**Island**	Max	19.9	165.4	8.3	10.9	76.3	7	3.2	28.7	8.8	2.5	17.3	6.8	2.4	10.1	4.3
Name:	**MOFB**	99th	7	45	6.5	4.2	29.5	7.1	3	16.4	5.5	2.5	12.6	5.1	2.3	10	4.3
Distn:	1	95th	3.1	13.2	4.3	3	14.4	4.8	2.5	11	4.3	2.4	7.8	3.3	2.1	6.5	3.1
Depth:	7.5	80th	1.9	4.2	2.2	1.8	4.9	2.8	1.8	5.8	3.3	1.8	5.7	3.2	1.7	5.4	3.1
Base.:	130	Med.	1.3	1.9	1.5	1.3	2.3	1.8	1.4	2.8	2.1	1.4	3	2.2	1.4	3	2.1
Dred.:	521	Mean	1.5	4.1	2.8	1.4	4.1	2.9	1.4	4.1	2.9	1.4	3.8	2.8	1.4	3.6	2.6
**Barrow**	**Island**	Max	19.9	370.3	18.6	10.9	152.9	14	3.2	56	17.3	2.8	32.9	11.7	2.8	26.4	9.5
Name:	**MOFC**	99th	6.7	78.8	11.8	4.2	50.3	11.9	2.9	46.6	15.9	2.8	31.6	11.4	2.8	25.1	9
Distn:	0.8	95th	3.1	25.2	8.1	2.9	25.4	8.8	2.8	20.7	7.4	2.8	19	6.9	2.8	23.4	8.5
Depth:	6.9	80th	2.1	6.4	3	2.1	7.8	3.6	2.3	11.1	4.8	2.2	11.4	5.1	2.1	12.4	5.9
Base.:	121	Med.	1.2	2.2	1.8	1.2	3	2.4	1.3	3.7	2.8	1.4	5.3	3.9	1.4	7.2	5.2

Table S1.A. Max, 99th, 95th, 80th percentiles, median and mean NTU values over 1 h, 1 d, 14 d, and 21 d running average period at all sites during the baseline period ("B", before dredging) or for during the duration of the dredging program ("D"). The ratio of dredging/baseline is also shown ("Δ"). For each site the distance from dredging activities (Distn), the site depth (Depth) and the number of sampling days during baseline (Base) and dredging (Dred) are shown. NTU values approximate total suspendid solid concentrations with a linear conversion factor of between 1.1 and 2.1.

Site	Details	Stat.	B1H	D1H	Δ1H	B1D	D1D	Δ1D	B1W	D1W	Δ1W	B2W	D2W	Δ2W	B3W	D3W	Δ3W
Dred.:	460	Mean	1.5	6.5	4.4	1.5	6.6	4.5	1.5	6.9	4.7	1.5	7.4	4.9	1.5	8	5.2
**Barrow**	**Island**	Max	40.1	51.6	1.3	19	30.1	1.6	8.3	7.6	0.9	6.7	6.4	1	6.1	4.7	0.8
Name:	**REFN**	99th	12	12.4	1	10.8	12	1.1	8	6.8	0.9	6.4	6.3	1	6	4.7	0.8
Distn:	28	95th	5	4.8	0.9	4.7	4.4	0.9	5.3	5.7	1.1	5	3.7	0.7	5.6	3.4	0.6
Depth:	7.2	80th	1.7	1.9	1.1	1.7	2.1	1.2	1.9	2.2	1.2	2	2.4	1.2	2.2	2.4	1.1
Base.:	91	Med.	0.9	0.9	1	0.9	0.9	1.1	0.9	1	1.1	1	1.2	1.2	1	1.3	1.3
Dred.:	370	Mean	1.5	1.5	1	1.5	1.5	1	1.5	1.6	1	1.6	1.6	1	1.6	1.6	1
**Barrow**	**Island**	Max	75.2	37.5	0.5	30.4	20.9	0.7	12.3	11.5	0.9	9.2	7.9	0.9	8.3	6	0.7
Name:	**REFS**	99th	19.4	14.5	0.7	16.8	13.4	0.8	12	10.8	0.9	9.1	6.7	0.7	7.9	5.3	0.7
Distn:	23.6	95th	7.5	5.4	0.7	6.8	4.8	0.7	7.3	3.9	0.5	8	3.2	0.4	5.9	3.6	0.6
Depth:	5	80th	3.9	1.8	0.5	3.9	1.8	0.5	4	1.9	0.5	4	1.8	0.5	4.1	1.7	0.4
Base.:	146	Med.	1.8	0.8	0.5	1.8	0.9	0.5	2	1	0.5	2	1.1	0.5	2.2	1.4	0.7
Dred.:	374	Mean	2.8	1.5	0.5	2.6	1.5	0.6	2.6	1.4	0.5	2.8	1.4	0.5	2.9	1.4	0.5
**Barrow**	**Island**	Max	75.2	44.6	0.6	30.4	31.4	1	12.3	8.4	0.7	9.2	6.6	0.7	8.3	5.1	0.6
Name:	**SBS**	99th	19.7	12.2	0.6	17.8	11.2	0.6	12	7.7	0.6	9.1	5.2	0.6	7.9	4	0.5
Distn:	29.9	95th	7.7	5	0.6	7.1	4.7	0.7	7.6	3.8	0.5	7.9	3.2	0.4	5.9	2.9	0.5
Depth:	4.7	80th	3.4	2.1	0.6	3.4	2.1	0.6	3.5	2.4	0.7	3.6	2.3	0.6	3.5	2.1	0.6
Base.:	599	Med.	1.5	1	0.7	1.6	1.1	0.7	1.7	1.2	0.7	1.8	1.3	0.8	1.9	1.4	0.7
Dred.:	454	Mean	2.5	1.6	0.6	2.4	1.6	0.7	2.4	1.6	0.6	2.5	1.5	0.6	2.5	1.4	0.6
**Barrow**	**Island**	Max	28.7	242.8	8.5	5.1	81.3	16	2	24.8	12.3	1.4	3.6	2.6	1.2	2	1.6
Name:	**T1**	99th	4.1	33.6	8.3	4.1	22	5.3	2	15.4	7.7	1.4	3.4	2.5	1.2	2	1.6
Distn:	1.8	95th	2	5.1	2.6	1.8	4.7	2.6	1.9	3.5	1.8	1.4	3.2	2.4	1.2	2	1.6
Depth:	6.6	80th	1.2	2.8	2.3	1.1	2.8	2.5	1.4	2.7	1.9	1.4	2.1	1.6	1.2	2	1.6
Base.:	81	Med.	0.9	1.7	1.9	0.9	1.7	1.9	0.9	1.8	1.9	1.3	1.9	1.5	1.2	2	1.6
Dred.:	558	Mean	1.1	3.2	2.9	1.1	2.6	2.4	1.1	2.2	2	1.2	1.8	1.6	1.2	2	1.6
**Barrow**	**Island**	Max	70.6	161.8	2.3	7.7	20.4	2.7	1	3.7	3.7	-	2.9	-	-	1.8	-
Name:	**T2**	99th	4.6	11.5	2.5	7.4	10.1	1.4	1	3.6	3.7	-	2.7	-	-	1.8	-
Distn:	1.5	95th	2.1	4.5	2.1	3.5	4.2	1.2	1	3.2	3.2	-	2.6	-	-	1.8	-
Depth:	6.6	80th	1.2	2.8	2.3	1.1	2.8	2.6	1	2.6	2.6	-	2.2	-	-	1.7	-
Base.:	81	Med.	0.9	1.9	2.1	1	2.1	2.1	1	2.1	2.1	-	1.7	-	-	1.6	-
Dred.:	455	Mean	1.2	2.5	2.1	1.3	2.4	1.8	1	2.2	2.2	-	1.8	-	-	1.6	-
**Barrow**	**Island**	Max	16.1	372	23.1	4.8	63.1	13.1	3.7	16	4.3	1	9.1	9.3	-	6.8	-
Name:	**T3**	99th	5.9	17.5	2.9	3.8	18.6	4.9	3.7	15.6	4.3	1	9.1	9.3	-	6.8	-
Distn:	1.1	95th	3.7	5	1.3	3.6	5.5	1.6	3.5	6.9	1.9	1	7.9	8	-	6.8	-
Depth:	6.6	80th	3.2	2.8	0.9	3.2	3	0.9	3.4	3.3	1	1	4.5	4.6	-	6	-
Base.:	81	Med.	1.1	2	1.8	1.1	2.1	1.8	1	2.4	2.3	1	2.4	2.5	-	3.9	-
Dred.:	511	Mean	1.8	3	1.6	1.8	2.8	1.6	1.6	3.1	1.9	1	3.4	3.5	-	3.8	-
**Barrow**	**Island**	Max	5.7	227.1	40.1	1.9	33.3	17.5	1.2	15	12.4	1	8.6	8.3	-	8	-
Name:	**T4**	99th	2.5	24.1	9.7	1.7	17.8	10.2	1.2	14.7	12.5	1	7.3	7.1	-	7.9	-
Distn:	0.9	95th	1.7	10.9	6.3	1.5	9.4	6.4	1.1	8.5	7.7	1	7	7	-	7.3	-
Depth:	6.6	80th	1.1	5.9	5.2	1.1	6.4	6	1	6.4	6.6	0.9	6.2	7	-	7	-
Base.:	81	Med.	0.8	3.6	4.5	0.8	3.8	4.5	0.8	4.2	5	0.7	3.2	4.5	-	3.3	-
Dred.:	548	Mean	0.9	4.7	5.1	0.9	4.6	5.1	0.8	4.5	5.3	0.8	4.1	5.1	-	4.5	-
**Barrow**	**Island**	Max	16.5	237.6	14.4	3.1	62.8	20.3	1.2	23	18.9	1.2	17.9	15.4	-	13.6	-
Name:	**T5**	99th	3.6	48.7	13.6	3	39.7	13.2	1.2	17.3	14.3	1.2	15.9	13.7	-	11.1	-
Distn:	0.7	95th	2	19.4	9.5	2.8	18.2	6.5	1.2	14	11.5	1.2	11.3	9.7	-	9	-
Depth:	6.6	80th	1.4	6.7	4.7	1.3	8	6.1	1.2	8.7	7.3	1.2	9.2	7.9	-	8.3	-
Base.:	81	Med.	1.1	3.9	3.4	1.2	4.4	3.7	1.1	5.8	5.2	1.2	6.7	5.7	-	6.9	-
Dred.:	558	Mean	1.3	6.5	5.1	1.3	6.5	5	1.1	6.6	5.8	1.2	7.1	6.1	-	7.1	-
**Barrow**	**Island**	Max	23.7	261.1	11	3	47	15.6	-	19.2	-	-	14.2	-	-	-	-
Name:	**T6**	99th	5.6	43.5	7.8	3	30.6	10.2	-	18.5	-	-	14.1	-	-	-	-
Distn:	0.5	95th	3.6	18.7	5.2	2.9	17.2	5.8	-	14.9	-	-	14	-	-	-	-
Depth:	6.6	80th	2.3	8.3	3.5	2.2	8.9	4	-	10	-	-	7.5	-	-	-	-
Base.:	79	Med.	1.5	5	3.4	1.6	5.7	3.6	-	6.4	-	-	6.3	-	-	-	-
Dred.:	523	Mean	1.8	7.1	4	1.8	7.3	4.2	-	7.5	-	-	7.5	-	-	-	-
**Barrow**	**Island**	Max	15.4	202.9	13.2	5.5	65.7	12	1.1	25.1	23.5	-	8.6	-	-	6.5	-
Name:	**T7**	99th	7.1	37.6	5.3	5.3	28.6	5.4	1.1	14.1	13.2	-	8.5	-	-	6.5	-
Distn:	1.4	95th	2.2	13	5.9	4.3	13.8	3.2	1.1	10.8	10.2	-	8.2	-	-	6.5	-
Depth:	6.6	80th	1.4	5.1	3.8	1.5	5.7	3.9	1.1	7.2	6.8	-	5.8	-	-	6.3	-
Base.:	74	Med.	1.1	3.2	2.9	1.2	3.3	2.8	1.1	3.9	3.6	-	4.3	-	-	5.2	-
Dred.:	566	Mean	1.3	4.9	3.7	1.5	4.9	3.3	1.1	4.8	4.5	-	4.7	-	-	5.4	-
**Barrow**	**Island**	Max	38.1	119.4	3.1	12.4	72.3	5.9	4.8	27	5.7	2.3	22.3	9.5	2.4	16.2	6.9
Name:	**TR**	99th	8	35.4	4.4	5.8	31.2	5.3	3.6	22.6	6.2	2.3	20.6	9	2.2	16	7.2

Table S1.A. Max, 99th, 95th, 80th percentiles, median and mean NTU values over 1 h, 1 d, 14 d, and 21 d running average period at all sites during the baseline period ("B", before dredging) or for during the duration of the dredging program ("D"). The ratio of dredging/baseline is also shown ("Δ"). For each site the distance from dredging activities (Distn), the site depth (Depth) and the number of sampling days during baseline (Base) and dredging (Dred) are shown. NTU values approximate total suspendid solid concentrations with a linear conversion factor of between 1.1 and 2.1.

**Site**	**Details**	**Stat.**	**B1H**	**D1H**	**Δ1H**	**B1D**	**D1D**	**Δ1D**	**B1W**	**D1W**	**Δ1W**	**B2W**	**D2W**	**Δ2W**	**B3W**	**D3W**	**Δ3W**
Distn:	5	95th	3.7	17.3	4.7	3.1	17.4	5.6	2.6	12.8	4.9	2	10.7	5.3	2.1	10.5	5.1
Depth:	4.5	80th	1.6	3.5	2.2	1.6	3.9	2.4	1.6	5.8	3.6	1.7	4.9	2.9	1.7	4	2.4
Base.:	267	Med.	1	1.4	1.4	1	1.5	1.5	1.1	1.9	1.7	1.1	1.8	1.7	1.1	1.8	1.6
Dred.:	450	Mean	1.3	3.7	2.8	1.3	3.7	2.8	1.3	3.7	2.9	1.2	3.4	2.7	1.3	2.9	2.3

Table S1.A. Max, 99th, 95th, 80th percentiles, median and mean NTU values over 1 h, 1 d, 14 d, and 21 d running average period at all sites during the baseline period ("B", before dredging) or for during the duration of the dredging program ("D"). The ratio of dredging/baseline is also shown ("Δ"). For each site the distance from dredging activities (Distn), the site depth (Depth) and the number of sampling days during baseline (Base) and dredging (Dred) are shown. NTU values approximate total suspendid solid concentrations with a linear conversion factor of between 1.1 and 2.1.

Site	Details	Stat.	B1H	D1H	Δ1H	B1D	D1D	Δ1D	B1W	D1W	Δ1W	B2W	D2W	Δ2W	B3W	D3W	Δ3W
**Site**	**Details**	**Stat.**	**B1H**	**D1H**	**Δ1H**	**B1D**	**D1D**	**Δ1D**	**B1W**	**D1W**	**Δ1W**	**B2W**	**D2W**	**Δ2W**	**B3W**	**D3W**	**Δ3W**
**Burrup.**	**Penin.**	Max	13.4	53.1	4	7.8	26.1	3.4	2.7	11.2	4.1	1.7	8.4	4.9	1.3	7	5.3
Name:	**ANG2**	99th	5.5	11.3	2.1	5.3	9.8	1.8	2.7	8.6	3.2	1.7	7.8	4.7	1.3	6.6	5
Distn:	10.1	95th	2.4	4	1.6	1.9	4	2.1	2.2	4.3	2	1.5	4.5	2.9	1.3	4.3	3.3
Depth:	2.6	80th	1.5	1.5	1	1.5	1.5	1	1.5	1.8	1.2	1.3	1.6	1.2	1.2	1.5	1.2
Base.:	15	Med.	0.9	0.9	1	1	0.9	0.9	0.9	1	1.1	0.9	1	1.1	1.1	1	0.9
Dred.:	887	Mean	1.1	1.4	1.3	1.1	1.4	1.3	1	1.5	1.4	1	1.4	1.5	1	1.4	1.4
**Burrup.**	**Penin.**	Max	-	268.2	-	-	37.7	-	-	14	-	-	10.4	-	-	8.8	-
Name:	**ANG3**	99th	-	16.5	-	-	12.5	-	-	12.7	-	-	10.2	-	-	8.6	-
Distn:	9.8	95th	-	6.5	-	-	6.6	-	-	7.3	-	-	9	-	-	7.8	-
Depth:	0.8	80th	-	2.7	-	-	2.9	-	-	2.8	-	-	3	-	-	3.1	-
Base.:	0	Med.	-	1.9	-	-	2.1	-	-	2.2	-	-	2.2	-	-	2.3	-
Dred.:	892	Mean	-	2.9	-	-	2.7	-	-	2.8	-	-	2.9	-	-	3	-
**Burrup.**	**Penin.**	Max	13.4	96.6	7.2	7.8	19.5	2.5	2.7	8.5	3.1	1.7	5.7	3.3	1.3	4.1	3.1
Name:	**ANGI**	99th	5.4	9.5	1.8	5.3	10.4	1.9	2.7	6.3	2.3	1.7	5.4	3.2	1.3	4	3
Distn:	13	95th	2.4	3.6	1.5	1.9	3.7	2	2.2	4.4	2	1.5	3.8	2.5	1.3	3.8	2.9
Depth:	5.6	80th	1.4	1.3	0.9	1.4	1.3	0.9	1.4	1.4	1	1.3	1.4	1.1	1.2	1.8	1.4
Base.:	123	Med.	0.9	0.7	0.8	0.9	0.8	0.8	0.9	0.9	0.9	0.9	0.9	1	1.1	0.9	0.8
Dred.:	922	Mean	1.1	1.2	1.1	1.1	1.2	1.2	1	1.2	1.2	1	1.2	1.3	1	1.3	1.3
**Burrup.**	**Penin.**	Max	15.2	463.1	30.5	5.8	118.4	20.5	4.2	60.5	14.2	3.9	56.8	14.6	-	46.3	-
Name:	**CHC4**	99th	6.6	196.2	29.6	5.1	113.5	22.1	4.2	60.4	14.2	3.9	56.5	14.5	-	46.3	-
Distn:	0.4	95th	4.7	113.1	24.1	4.2	95.8	22.6	4.1	58.1	14	3.9	55.6	14.3	-	46.2	-
Depth:	1.9	80th	3	53	17.4	3.3	54.6	16.6	3.7	51.9	14.1	3.9	50.4	13.1	-	46.1	-
Base.:	96	Med.	1.4	21.4	15.4	1.4	27.6	19.4	2	43.9	22	3.8	45	11.8	-	45.9	-
Dred.:	49	Mean	1.9	35.4	18.4	2	34.9	17.7	2.5	34.6	13.7	3.8	42.3	11.1	-	45.8	-
**Burrup.**	**Penin.**	Max	13.4	183.1	13.7	7.8	26.2	3.4	2.7	10	3.7	1.9	6.6	3.4	1.3	5.5	4.2
Name:	**COBN**	99th	5.4	9.9	1.8	5.3	9.2	1.7	2.7	7	2.6	1.9	5.5	2.9	1.3	4.9	3.7
Distn:	8.4	95th	2.5	4.4	1.7	2.2	4.3	2	2.2	4.3	2	1.6	4.2	2.6	1.3	4.2	3.2
Depth:	2.5	80th	1.6	2.2	1.4	1.6	2.2	1.3	1.5	2.5	1.6	1.3	2.4	1.8	1.2	2.2	1.7
Base.:	15	Med.	0.9	1.3	1.4	1	1.3	1.4	0.9	1.3	1.5	0.9	1.3	1.4	1.1	1.2	1.1
Dred.:	971	Mean	1.2	1.8	1.6	1.1	1.8	1.6	1.1	1.8	1.7	1	1.8	1.8	1	1.7	1.7
**Burrup.**	**Penin.**	Max	-	143.4	-	-	50.2	-	-	16.2	-	-	9.1	-	-	1.7	-
Name:	**CONI**	99th	-	18.8	-	-	19.9	-	-	11.5	-	-	7.9	-	-	1.7	-
Distn:	7.3	95th	-	5.5	-	-	5.6	-	-	4.9	-	-	3.9	-	-	1.7	-
Depth:	2.8	80th	-	2.5	-	-	2.6	-	-	2	-	-	1.6	-	-	1.4	-
Base.:	0	Med.	-	1.5	-	-	1.3	-	-	1.1	-	-	1.1	-	-	1.1	-
Dred.:	884	Mean	-	2.3	-	-	2.3	-	-	1.7	-	-	1.5	-	-	1.1	-
**Burrup.**	**Penin.**	Max	-	92.3	-	-	38.1	-	-	14.7	-	-	9.2	-	-	6.5	-
Name:	**CRTS**	99th	-	9.9	-	-	10.3	-	-	11.6	-	-	8.8	-	-	6.5	-
Distn:	14.7	95th	-	2.3	-	-	2.1	-	-	3.4	-	-	5.6	-	-	5.9	-
Depth:	5.5	80th	-	1.2	-	-	1.2	-	-	1.2	-	-	1.2	-	-	1.5	-
Base.:	0	Med.	-	0.7	-	-	0.8	-	-	0.8	-	-	0.8	-	-	0.8	-
Dred.:	982	Mean	-	1.1	-	-	1.1	-	-	1.2	-	-	1.3	-	-	1.4	-
**Burrup.**	**Penin.**	Max	15.2	187.9	12.4	5.8	79	13.7	4.2	55.5	13.1	3.9	43.2	11.1	-	35.6	-
Name:	**DPAN**	99th	6.7	101.3	15.2	5.1	77.4	15.1	4.2	55.4	13.1	3.9	43	11	-	35.5	-
Distn:	0.6	95th	4.7	71.4	15.3	4.2	66.3	15.7	4.1	53	12.8	3.9	42.6	10.9	-	34.8	-
Depth:	0	80th	3	48.3	15.9	3.3	50.9	15.6	3.7	47.7	12.9	3.9	39.6	10.3	-	31.6	-
Base.:	5	Med.	1.4	29.3	21.3	1.4	30.7	21.5	2	34.4	17.2	3.8	37.5	9.9	-	30.2	-
Dred.:	47	Mean	1.9	31.3	16.5	2	31.2	16	2.5	32.9	13	3.8	36.3	9.6	-	30.6	-
**Burrup.**	**Penin.**	Max	-	111.3	-	-	32	-	-	10.6	-	-	6.6	-	-	5	-
Name:	**ELI1**	99th	-	16.2	-	-	11	-	-	6.8	-	-	5.5	-	-	4.9	-
Distn:	7	95th	-	4	-	-	4.3	-	-	3.3	-	-	2.5	-	-	2.2	-
Depth:	3.5	80th	-	1.8	-	-	2.1	-	-	2.2	-	-	2.1	-	-	1.9	-
Base.:	0	Med.	-	1.2	-	-	1.3	-	-	1.6	-	-	1.6	-	-	1.6	-
Dred.:	908	Mean	-	1.8	-	-	1.8	-	-	1.8	-	-	1.7	-	-	1.7	-
**Burrup.**	**Penin.**	Max	13.4	28.8	2.2	7.8	9.2	1.2	2.7	7.7	2.8	1.9	5.3	2.8	1.3	4.8	3.6
Name:	**FFP1**	99th	5.6	7.6	1.3	5.3	5.9	1.1	2.7	5.2	1.9	1.9	5.2	2.8	1.3	4.7	3.5
Distn:	13.8	95th	2.6	4.1	1.6	2.2	3.9	1.8	2.2	3.5	1.6	1.6	3.6	2.2	1.3	3.7	2.9
Depth:	2.5	80th	1.6	2.4	1.5	1.6	2.3	1.5	1.5	2.3	1.5	1.4	2.1	1.5	1.2	2.1	1.7
Base.:	16	Med.	0.9	1.5	1.6	1	1.6	1.6	0.9	1.7	1.8	0.9	1.7	1.9	1.1	1.7	1.5
Dred.:	984	Mean	1.2	1.9	1.6	1.1	1.9	1.6	1.1	1.9	1.7	1	1.9	1.9	1	1.9	1.9

Table S1.A. Max, 99th, 95th, 80th percentiles, median and mean NTU values over 1 h, 1 d, 14 d, and 21 d running average period at all sites during the baseline period ("B", before dredging) or for during the duration of the dredging program ("D"). The ratio of dredging/baseline is also shown ("Δ"). For each site the distance from dredging activities (Distn), the site depth (Depth) and the number of sampling days during baseline (Base) and dredging (Dred) are shown. NTU values approximate total suspendid solid concentrations with a linear conversion factor of between 1.1 and 2.1.

Site	Details	Stat.	B1H	D1H	Δ1H	B1D	D1D	Δ1D	B1W	D1W	Δ1W	B2W	D2W	Δ2W	B3W	D3W	Δ3W
**Burrup.**	**Penin.**	Max	13.4	253.5	19	7.8	40.6	5.2	2.7	16	5.9	1.7	11.3	6.6	1.3	8.7	6.6
Name:	**GIDI**	99th	5.4	16.3	3	5.4	16.1	3	2.7	11.7	4.3	1.7	10	6	1.3	8.5	6.4
Distn:	14.6	95th	2.4	5.6	2.4	1.9	5.4	2.8	2.4	7.1	3	1.5	6.7	4.4	1.3	5.7	4.3
Depth:	5	80th	1.4	2	1.4	1.4	2	1.4	1.4	2	1.4	1.3	1.9	1.4	1.2	2	1.6
Base.:	10	Med.	0.8	0.9	1.1	0.9	1.1	1.2	0.9	1.2	1.4	0.9	1.2	1.4	1.1	1.3	1.1
Dred.:	956	Mean	1.1	1.8	1.7	1	1.8	1.7	1	1.9	1.9	1	1.8	1.9	1	1.9	1.9
**Burrup.**	**Penin.**	Max	-	263.4	-	-	57.5	-	-	2.9	-	-	1.9	-	-	1.6	-
Name:	**HAM3**	99th	-	8.2	-	-	6.2	-	-	2.6	-	-	1.8	-	-	1.5	-
Distn:	24.5	95th	-	3.7	-	-	3.1	-	-	1.8	-	-	1.4	-	-	1.2	-
Depth:	7.4	80th	-	1.8	-	-	1.6	-	-	1.2	-	-	1.1	-	-	1	-
Base.:	0	Med.	-	1.1	-	-	1	-	-	0.9	-	-	0.9	-	-	0.9	-
Dred.:	843	Mean	-	1.6	-	-	1.3	-	-	1	-	-	0.9	-	-	0.9	-
**Burrup.**	**Penin.**	Max	13.4	36.9	2.8	7.8	13.8	1.8	2.7	6.6	2.4	1.7	5	2.9	1.3	4.2	3.2
Name:	**HAM4**	99th	5.4	7.9	1.4	5.3	6.1	1.1	2.7	5.3	2	1.7	4.6	2.8	1.3	4.1	3.1
Distn:	26.4	95th	2.3	3.6	1.5	1.9	3.2	1.7	2.2	2.6	1.2	1.5	2.7	1.8	1.3	2.4	1.8
Depth:	6.2	80th	1.3	1.5	1.1	1.3	1.5	1.1	1.3	1.5	1.1	1.3	1.3	1	1.2	1.3	1
Base.:	16	Med.	0.8	0.7	0.9	0.8	0.9	1	0.8	1	1.2	0.9	1	1.1	1.1	1	0.9
Dred.:	892	Mean	1	1.2	1.2	1	1.2	1.2	1	1.2	1.2	1	1.2	1.2	1	1.1	1.1
**Burrup.**	**Penin.**	Max	13.4	160.1	12	7.8	14.9	1.9	2.7	7.4	2.7	1.7	6.3	3.7	1.3	3.2	2.4
Name:	**HGPT**	99th	5.4	7.3	1.3	5.4	6.5	1.2	2.7	6	2.2	1.7	4.9	2.9	1.3	3.1	2.4
Distn:	9	95th	2.4	2.9	1.3	1.9	2.7	1.4	2.3	2.3	1	1.5	1.9	1.3	1.3	1.9	1.5
Depth:	2.8	80th	1.4	1.7	1.2	1.4	1.7	1.2	1.4	1.6	1.1	1.3	1.5	1.1	1.2	1.4	1.1
Base.:	12	Med.	0.9	1.1	1.2	1	1.1	1.2	0.9	1.1	1.3	0.9	1	1.2	1.1	1	0.9
Dred.:	914	Mean	1.1	1.4	1.3	1.1	1.4	1.3	1	1.3	1.3	1	1.2	1.2	1	1.1	1.1
**Burrup.**	**Penin.**	Max	15.2	425.1	28	5.8	247.2	42.9	4.2	104.8	24.7	3.9	55.5	14.3	-	34.1	-
Name:	**HOLD**	99th	6.6	227.4	34.6	5.1	189.2	37	4.2	103.9	24.5	3.9	55.5	14.2	-	34	-
Distn:	0.3	95th	4.6	97.9	21.3	4.2	96.4	22.9	4.1	84	20.3	3.9	53.8	13.8	-	33.3	-
Depth:	-	80th	3	36.4	12.2	3.3	39.8	12.2	3.7	36.5	9.9	3.9	32.7	8.5	-	31.1	-
Base.:	5	Med.	1.3	15.7	11.6	1.4	18.2	13.4	2	26.5	13.3	3.8	25.3	6.7	-	21.2	-
Dred.:	83	Mean	1.8	28	15.2	1.9	27.9	14.8	2.5	27.7	11	3.8	23.4	6.2	-	20.9	-
**Burrup.**	**Penin.**	Max	67	128.7	1.9	16.6	63.2	3.8	2.7	24.8	9.1	1.4	17.3	12.2	-	13.3	-
Name:	**KGBY**	99th	19.3	34.1	1.8	15.6	21.8	1.4	2.7	23.9	8.9	1.4	16.6	11.7	-	12.9	-
Distn:	4	95th	6.6	11.6	1.8	4.8	9.1	1.9	2.7	9.6	3.6	1.4	12.4	8.7	-	11.5	-
Depth:	1.4	80th	2.1	3.5	1.6	2.5	4.2	1.7	2.4	4.3	1.8	1.4	4.4	3.1	-	4.7	-
Base.:	114	Med.	1	1.7	1.6	1.4	2.2	1.6	1.5	2.6	1.8	1.4	2.9	2.1	-	2.9	-
Dred.:	920	Mean	2.1	3.4	1.6	2.1	3.4	1.6	1.7	3.6	2.1	1.4	3.9	2.8	-	4	-
**Burrup.**	**Penin.**	Max	-	56.8	-	-	15.9	-	-	6.2	-	-	4.3	-	-	3.4	-
Name:	**LANI**	99th	-	7.9	-	-	6.8	-	-	5.4	-	-	4.1	-	-	3.3	-
Distn:	20.9	95th	-	2.8	-	-	2.9	-	-	2.6	-	-	2.8	-	-	2.6	-
Depth:	1.8	80th	-	1	-	-	1	-	-	1.2	-	-	1.3	-	-	1.2	-
Base.:	0	Med.	-	0.5	-	-	0.6	-	-	0.6	-	-	0.7	-	-	0.7	-
Dred.:	983	Mean	-	0.9	-	-	0.9	-	-	0.9	-	-	1	-	-	1	-
**Burrup.**	**Penin.**	Max	13.4	17.3	1.3	7.8	9.2	1.2	2.7	6	2.2	1.7	4	2.4	1.3	3	2.3
Name:	**LEGD**	99th	5.5	4.6	0.8	5.4	4.6	0.9	2.7	3.9	1.4	1.7	3	1.8	1.3	2.3	1.7
Distn:	28.6	95th	2.4	1.3	0.5	1.9	1.2	0.6	2.4	1.3	0.5	1.5	1.1	0.7	1.3	1.3	1
Depth:	9.2	80th	1.3	0.6	0.5	1.4	0.6	0.5	1.4	0.7	0.5	1.3	0.7	0.5	1.2	0.7	0.5
Base.:	11	Med.	0.8	0.4	0.5	0.8	0.4	0.4	0.9	0.4	0.4	0.9	0.4	0.5	1.1	0.4	0.4
Dred.:	871	Mean	1	0.5	0.5	1	0.5	0.5	1	0.5	0.6	1	0.5	0.5	1	0.5	0.5
**Burrup.**	**Penin.**	Max	-	66.9	-	-	25.7	-	-	8.6	-	-	5.5	-	-	4.4	-
Name:	**MAL2**	99th	-	13.6	-	-	9.2	-	-	8.5	-	-	5.4	-	-	4.3	-
Distn:	13.7	95th	-	5	-	-	5	-	-	4.2	-	-	5.3	-	-	4.2	-
Depth:	-	80th	-	2.3	-	-	2.4	-	-	2.3	-	-	2.3	-	-	2.5	-
Base.:	0	Med.	-	1.5	-	-	1.6	-	-	1.6	-	-	1.7	-	-	1.7	-
Dred.:	902	Mean	-	2.1	-	-	2.1	-	-	2	-	-	2.1	-	-	2.2	-
**Burrup.**	**Penin.**	Max	-	61.8	-	-	28.7	-	-	9.8	-	-	6.3	-	-	4.7	-
Name:	**MALI**	99th	-	5.1	-	-	3.7	-	-	3.2	-	-	3.5	-	-	1.5	-
Distn:	9.9	95th	-	2.3	-	-	2.1	-	-	1.8	-	-	1.5	-	-	1.4	-
Depth:	3.5	80th	-	1.5	-	-	1.4	-	-	1.4	-	-	1.3	-	-	1.2	-
Base.:	0	Med.	-	1	-	-	1.1	-	-	1.1	-	-	1.1	-	-	1.1	-
Dred.:	923	Mean	-	1.3	-	-	1.3	-	-	1.2	-	-	1.2	-	-	1.1	-
**Burrup.**	**Penin.**	Max	-	75.3	-	-	26.7	-	-	6.3	-	-	4.6	-	-	4.1	-
Name:	**MIDI**	99th	-	14	-	-	11.2	-	-	5.9	-	-	4.5	-	-	4	-
Distn:	12.1	95th	-	6.9	-	-	6.2	-	-	5.3	-	-	3.3	-	-	3.2	-

Table S1.A. Max, 99th, 95th, 80th percentiles, median and mean NTU values over 1 h, 1 d, 14 d, and 21 d running average period at all sites during the baseline period ("B", before dredging) or for during the duration of the dredging program ("D"). The ratio of dredging/baseline is also shown ("Δ"). For each site the distance from dredging activities (Distn), the site depth (Depth) and the number of sampling days during baseline (Base) and dredging (Dred) are shown. NTU values approximate total suspendid solid concentrations with a linear conversion factor of between 1.1 and 2.1.

Site	Details	Stat.	B1H	D1H	Δ1H	B1D	D1D	Δ1D	B1W	D1W	Δ1W	B2W	D2W	Δ2W	B3W	D3W	Δ3W
Depth:	3.2	80th	-	4.2	-	-	4.1	-	-	3.1	-	-	3	-	-	3	-
Base.:	0	Med.	-	2.7	-	-	2.6	-	-	2.2	-	-	2.1	-	-	2	-
Dred.:	933	Mean	-	3.4	-	-	3.3	-	-	2.6	-	-	2.4	-	-	2.3	-
**Burrup.**	**Penin.**	Max	-	54.6	-	-	16.7	-	-	10.3	-	-	6.3	-	-	4.5	-
Name:	**MIDR**	99th	-	9.5	-	-	11.9	-	-	10.1	-	-	6.1	-	-	4.4	-
Distn:	15.9	95th	-	2.6	-	-	2.9	-	-	2.4	-	-	3.1	-	-	3.3	-
Depth:	4.2	80th	-	1.6	-	-	1.3	-	-	1.2	-	-	1.4	-	-	1.5	-
Base.:	0	Med.	-	1.1	-	-	0.9	-	-	0.8	-	-	0.8	-	-	0.8	-
Dred.:	860	Mean	-	1.4	-	-	1.3	-	-	1.2	-	-	1.1	-	-	1	-
**Burrup.**	**Penin.**	Max	-	120.2	-	-	9.1	-	-	3	-	-	2.5	-	-	2	-
Name:	**NELS**	99th	-	4	-	-	4	-	-	2.6	-	-	2.1	-	-	1.9	-
Distn:	17.7	95th	-	2.1	-	-	2.3	-	-	2.2	-	-	1.8	-	-	1.9	-
Depth:	3.2	80th	-	1.3	-	-	1.2	-	-	1.4	-	-	1.6	-	-	1.6	-
Base.:	0	Med.	-	0.9	-	-	0.9	-	-	0.7	-	-	0.7	-	-	0.7	-
Dred.:	867	Mean	-	1.1	-	-	1	-	-	1	-	-	1	-	-	1	-
**Burrup.**	**Penin.**	Max	20.7	81.2	3.9	5.8	42.6	7.4	4.2	13	3.1	3.9	8.2	2.1	-	6.7	-
Name:	**NWIT**	99th	7	14.3	2.1	5.1	13.7	2.7	4.2	10.7	2.5	3.9	8	2.1	-	6.1	-
Distn:	4.3	95th	4.7	4.8	1	4.2	4.5	1.1	4.1	5.8	1.4	3.9	6	1.6	-	5.7	-
Depth:	2.3	80th	3	1.9	0.6	3.2	1.9	0.6	3.6	2.1	0.6	3.9	2.3	0.6	-	3	-
Base.:	15	Med.	1.5	1.1	0.8	1.7	1.2	0.8	2.2	1.3	0.6	3.8	1.3	0.4	-	1.4	-
Dred.:	881	Mean	2	1.8	0.9	2	1.8	0.9	2.5	1.9	0.8	3.6	2	0.6	-	2.1	-
**Burrup.**	**Penin.**	Max	15.2	77.9	5.1	5.8	41.9	7.3	4.2	12.2	2.9	3.9	7.8	2	-	6.3	-
Name:	**SCON**	99th	7	16.1	2.3	5.1	13.3	2.6	4.2	9.1	2.2	3.9	7.5	1.9	-	6.2	-
Distn:	5.3	95th	4.7	6.2	1.3	4.2	5.7	1.4	4.1	5.3	1.3	3.9	5.4	1.4	-	5.6	-
Depth:	2	80th	3	2.2	0.7	3.2	2.6	0.8	3.6	2.8	0.8	3.9	2.9	0.8	-	2.7	-
Base.:	15	Med.	1.5	1.3	0.9	1.7	1.4	0.8	2.2	1.5	0.7	3.8	1.4	0.4	-	1.4	-
Dred.:	979	Mean	2	2.2	1.1	2	2.2	1.1	2.5	2.1	0.9	3.6	2.1	0.6	-	2.1	-
**Burrup.**	**Penin.**	Max	-	172.8	-	-	67.7	-	-	22	-	-	16.5	-	-	13.6	-
Name:	**SUP2**	99th	-	26	-	-	31.5	-	-	21.7	-	-	16.1	-	-	13.5	-
Distn:	1.8	95th	-	10.1	-	-	9.9	-	-	10.1	-	-	15.1	-	-	12.9	-
Depth:	2.2	80th	-	5.4	-	-	4.9	-	-	4.5	-	-	4.4	-	-	4.5	-
Base.:	0	Med.	-	3.6	-	-	3.2	-	-	2.7	-	-	2.7	-	-	2.6	-
Dred.:	866	Mean	-	4.7	-	-	4.4	-	-	4	-	-	4.1	-	-	4.1	-
**Burrup.**	**Penin.**	Max	-	71.1	-	-	31.5	-	-	11.4	-	-	8.3	-	-	6.4	-
Name:	**SWIT**	99th	-	14.2	-	-	11.5	-	-	10.5	-	-	8.2	-	-	6.4	-
Distn:	2.9	95th	-	5.8	-	-	5.6	-	-	6.5	-	-	7.3	-	-	6.1	-
Depth:	3	80th	-	2.9	-	-	2.8	-	-	2.3	-	-	2.2	-	-	2.3	-
Base.:	0	Med.	-	1.7	-	-	1.6	-	-	1.4	-	-	1.5	-	-	1.4	-
Dred.:	928	Mean	-	2.4	-	-	2.2	-	-	2	-	-	2.1	-	-	2	-
**Burrup.**	**Penin.**	Max	37.5	132.2	3.5	19.4	23.8	1.2	7.6	8.7	1.1	6	6	1	4.9	5.2	1.1
Name:	**WINI**	99th	19.8	17.4	0.9	13.6	13.7	1	7.4	8.1	1.1	6	5.9	1	4.9	5.1	1
Distn:	16.5	95th	11.7	8.1	0.7	8.6	6.7	0.8	6.7	3.9	0.6	5.6	5.7	1	4.9	4.9	1
Depth:	1.4	80th	4	3.9	1	4.8	3.5	0.7	4.7	3	0.6	4.8	2.8	0.6	4.5	2.6	0.6
Base.:	106	Med.	1.5	2.4	1.6	1.9	2.3	1.2	3	2.2	0.8	3.1	2.2	0.7	2.8	2.3	0.8
Dred.:	907	Mean	3.1	3.3	1.1	3.1	3	1	3.3	2.6	0.8	3.2	2.6	0.8	2.9	2.6	0.9

Table S1.A. Max, 99th, 95th, 80th percentiles, median and mean NTU values over 1 h, 1 d, 14 d, and 21 d running average period at all sites during the baseline period ("B", before dredging) or for during the duration of the dredging program ("D"). The ratio of dredging/baseline is also shown ("Δ"). For each site the distance from dredging activities (Distn), the site depth (Depth) and the number of sampling days during baseline (Base) and dredging (Dred) are shown. NTU values approximate total suspendid solid concentrations with a linear conversion factor of between 1.1 and 2.1.

Site	Details	Stat.	B1H	D1H	Δ1H	B1D	D1D	Δ1D	B1W	D1W	Δ1W	B2W	D2W	Δ2W	B3W	D3W	Δ3W
**Cape**	**Lamb.**	Max	553.4	166.7	0.3	332.7	85.9	0.3	14.4	26.4	1.8	13.2	14.5	1.1	10.6	13.1	1.2
Name:	**BLR**	99th	94.2	41.3	0.4	40.3	37.3	0.9	12.5	22.8	1.8	11.6	13.9	1.2	9.6	12.1	1.3
Distn:	4.5	95th	16.7	18.4	1.1	13.2	19	1.4	9.1	14.5	1.6	7.9	12.2	1.6	7	9.9	1.4
Depth:	3	80th	5.4	4	0.7	5.3	4.4	0.8	5.2	6	1.2	4.6	6.5	1.4	3.7	6.1	1.7
Base.:	457	Med.	1.4	1.2	0.8	1.6	1.2	0.8	1.6	1.5	0.9	1.3	2.1	1.6	0.9	2.8	3.2
Dred.:	699	Mean	6.1	3.9	0.6	4.8	4	0.8	2.9	3.8	1.3	2.6	3.7	1.4	2.2	3.7	1.7
**Cape**	**Lamb.**	Max	81	97	1.2	26.6	38.1	1.4	10.6	16.1	1.5	6.3	9.7	1.5	4.7	7.8	1.6
Name:	**BTR**	99th	20.7	20.6	1	17.7	19	1.1	9.5	12.2	1.3	6.3	9	1.4	4.5	7.3	1.6
Distn:	1.8	95th	9.6	9.3	1	7.5	8.9	1.2	7.2	8.8	1.2	5.3	7.4	1.4	4.1	6.4	1.5
Depth:	12	80th	3.5	3.5	1	3.3	3.5	1.1	3.6	4.3	1.2	3.9	3.8	1	3	3.8	1.2
Base.:	467	Med.	1.4	1.5	1.1	1.5	1.7	1.1	1.6	1.9	1.2	1.4	2.2	1.5	0.6	2.6	4.1
Dred.:	646	Mean	2.7	2.8	1	2.5	2.8	1.1	2.4	2.8	1.2	2.1	2.7	1.3	1.5	2.7	1.8
**Cape**	**Lamb.**	Max	153.7	162.9	1.1	32.6	65.8	2	11.3	27.1	2.4	5	16.4	3.3	4	11.4	2.8
Name:	**BZI**	99th	20.1	43.1	2.2	17.7	36.9	2.1	7.9	19.5	2.5	4.9	15.8	3.2	3.9	11.2	2.9
Distn:	2.6	95th	8.8	13.7	1.6	7.4	13.1	1.8	5.4	10.9	2	3.5	9.2	2.7	3.7	7.9	2.2
Depth:	3	80th	2.8	2.9	1	3	3.3	1.1	2.8	3.8	1.3	2.5	5	2	2.5	4.8	1.9
Base.:	536	Med.	0.9	1	1.2	1.2	1.3	1.1	1.5	1.7	1.1	1.8	2	1.1	1.8	2.5	1.4
Dred.:	689	Mean	2.3	3.2	1.4	2.2	3.3	1.5	2	3.2	1.6	1.8	3.2	1.8	1.8	3.2	1.8
**Cape**	**Lamb.**	Max	166.6	120.7	0.7	54.4	58.3	1.1	28	24.2	0.9	19.8	15.7	0.8	13.8	11.5	0.8
Name:	**BZR**	99th	26.8	38.5	1.4	30.1	34.3	1.1	16.1	20.5	1.3	12.3	15.2	1.2	4.4	11.4	2.6
Distn:	2.9	95th	10.1	15.1	1.5	9	15	1.7	7.9	13.9	1.7	5.8	11.8	2.1	3.8	10.5	2.8
Depth:	4	80th	3	4	1.3	2.9	4.3	1.5	2.9	6.1	2.1	2.9	6.8	2.4	2.6	6.6	2.6
Base.:	388	Med.	1	1.4	1.5	1.1	1.6	1.4	1.3	1.8	1.4	1.3	2.1	1.6	1.2	2.6	2.2
Dred.:	689	Mean	2.7	3.7	1.4	2.7	3.7	1.4	2.2	3.9	1.7	2	3.9	2	1.7	3.9	2.4
**Cape**	**Lamb.**	Max	79.5	207.6	2.6	23.6	103	4.4	9.7	39.9	4.1	6.6	27.4	4.1	5.6	20.2	3.6
Name:	**CLW**	99th	17.2	58.5	3.4	13.9	53	3.8	8.8	33.7	3.9	6.4	25.6	4	5.4	19.7	3.6
Distn:	3.2	95th	9.4	24	2.6	8.3	24.2	2.9	7.1	20.3	2.8	5.7	18.9	3.3	4.9	17.1	3.5
Depth:	4	80th	3.5	5.5	1.6	3.5	6	1.7	3.2	7.4	2.3	3.7	8.9	2.4	3.8	9.5	2.5
Base.:	381	Med.	1.1	1.6	1.4	1.3	1.8	1.4	1.5	2.5	1.7	1.7	2.9	1.7	1.7	3.4	2
Dred.:	682	Mean	2.5	5.3	2.1	2.4	5.3	2.2	2.3	5.4	2.4	2.2	5.5	2.5	2.2	5.5	2.5
**Cape**	**Lamb.**	Max	165.7	217.3	1.3	52.6	82.1	1.6	20.1	32.3	1.6	14.1	26.7	1.9	10.4	20	1.9
Name:	**DIE**	99th	27.6	58.2	2.1	25.4	55	2.2	18.7	29.1	1.6	12.9	24.7	1.9	10.3	18.5	1.8
Distn:	12.7	95th	11.2	20.2	1.8	11.6	21.5	1.8	10.7	20.9	1.9	8.5	16.7	2	8.7	15	1.7
Depth:	3	80th	3.3	2.9	0.9	3.4	3.3	1	4.3	5.1	1.2	4.3	6.5	1.5	3.9	6.8	1.7
Base.:	448	Med.	1.3	1	0.8	1.5	1.1	0.7	1.8	1.3	0.7	2.1	1.7	0.8	2.3	2.3	1
Dred.:	695	Mean	3	4.2	1.4	3	4.2	1.4	3.1	4	1.3	3	4.2	1.4	2.8	4.3	1.5
**Cape**	**Lamb.**	Max	114.4	183.3	1.6	46.4	66.1	1.4	28	24.5	0.9	20.5	16.8	0.8	3.4	12.7	3.7
Name:	**DLI**	99th	26.2	30.9	1.2	23.5	30.6	1.3	21.7	21.2	1	15	14.9	1	3.4	12.4	3.7
Distn:	17.7	95th	8.3	7.8	0.9	7.6	8.5	1.1	4.5	11.9	2.6	3	11.4	3.8	2.6	8.9	3.4
Depth:	9	80th	2	1.7	0.8	2.1	1.8	0.9	2.2	1.8	0.8	2.3	3.2	1.4	2.1	4.5	2.2
Base.:	425	Med.	0.7	0.8	1.1	0.8	0.9	1	1.2	0.9	0.8	1.3	1	0.8	1.4	1.1	0.8
Dred.:	675	Mean	2.1	2.2	1	2.1	2.2	1.1	2	2.3	1.2	1.7	2.5	1.4	1.5	2.6	1.7
**Cape**	**Lamb.**	Max	6.5	207.9	31.9	1.8	58.3	32	1.4	18.3	13.4	-	10.8	-	-	8.4	-
Name:	**DOI**	99th	2.5	26.9	10.7	1.8	21	11.9	1.4	12.4	9.1	-	10.6	-	-	7.9	-
Distn:	35.4	95th	2.2	8.9	4	1.7	8.7	5	1.4	9	6.6	-	7.1	-	-	7.3	-
Depth:	7	80th	1.5	2.8	1.9	1.5	3.1	2	1.3	3.5	2.7	-	3.6	-	-	3.9	-
Base.:	13	Med.	1.2	1.7	1.4	1.2	1.8	1.5	1.1	2.1	1.9	-	2.3	-	-	2.5	-
Dred.:	686	Mean	1.2	2.9	2.3	1.2	2.9	2.4	1.2	2.9	2.5	-	2.9	-	-	2.9	-
**Cape**	**Lamb.**	Max	77.4	174.5	2.3	32.5	101.5	3.1	17.9	40	2.2	9.9	25.2	2.5	-	18.8	-
Name:	**DPI**	99th	44.5	41.1	0.9	32.2	37.2	1.2	17.9	26	1.5	9.9	20.3	2	-	14.1	-
Distn:	51.3	95th	34.6	13.7	0.4	29.1	14	0.5	17.7	13.6	0.8	9.9	10.2	1	-	9	-
Depth:	5	80th	18.4	3.5	0.2	18.6	3.7	0.2	16.7	4.1	0.2	9.9	3.6	0.4	-	3.5	-
Base.:	15	Med.	5.5	1.1	0.2	8.2	1.1	0.1	12.5	1.4	0.1	9.9	1.5	0.2	-	1.4	-
Dred.:	644	Mean	9.8	3.5	0.4	10.1	3.4	0.3	11.8	3.1	0.3	9.9	2.8	0.3	-	2.6	-
**Cape**	**Lamb.**	Max	86.3	182.4	2.1	38.2	93.7	2.5	18.6	44.9	2.4	11	23.9	2.2	8.1	18.8	2.3
Name:	**HAT**	99th	23.3	50.7	2.2	23	48.1	2.1	18.3	35.1	1.9	11	22.7	2.1	8.1	18	2.2
Distn:	13.9	95th	10.6	19	1.8	9.7	18.7	1.9	11.9	20.3	1.7	10.6	18.5	1.7	3.4	14.9	4.4
Depth:	4	80th	4.5	5.4	1.2	4.3	6.1	1.4	3.9	6.9	1.8	3.1	7.2	2.3	0.6	6.6	10.2
Base.:	389	Med.	1.4	1.3	0.9	1.4	1.4	1	1.2	1.8	1.5	0.6	3	5	0.6	3.4	6.1
Dred.:	661	Mean	3.1	4.6	1.5	2.9	4.6	1.6	2.7	4.9	1.8	2	4.8	2.4	1	4.8	4.9

Table S1.A. Max, 99th, 95th, 80th percentiles, median and mean NTU values over 1 h, 1 d, 14 d, and 21 d running average period at all sites during the baseline period ("B", before dredging) or for during the duration of the dredging program ("D"). The ratio of dredging/baseline is also shown ("Δ"). For each site the distance from dredging activities (Distn), the site depth (Depth) and the number of sampling days during baseline (Base) and dredging (Dred) are shown. NTU values approximate total suspendid solid concentrations with a linear conversion factor of between 1.1 and 2.1.

**Site**	**Details**	**Stat.**	**B1H**	**D1H**	**Δ1H**	**B1D**	**D1D**	**Δ1D**	**B1W**	**D1W**	**Δ1W**	**B2W**	**D2W**	**Δ2W**	**B3W**	**D3W**	**Δ3W**
**Cape**	**Lamb.**	Max	206.8	247.2	1.2	61.8	127.3	2.1	27.6	77.7	2.8	21	50.3	2.4	15.1	37.2	2.5
Name:	**MAN**	99th	38.1	85.4	2.2	39.7	81.6	2.1	27	56.8	2.1	20.8	49.4	2.4	14.9	35.5	2.4
Distn:	7.3	95th	17.5	34.5	2	16	34.2	2.1	15	35.6	2.4	14.9	31.7	2.1	13	31.6	2.4
Depth:	3	80th	7	7.8	1.1	6.8	7.8	1.1	5.8	10.4	1.8	5.4	13.6	2.5	5.3	13.4	2.5
Base.:	354	Med.	2.3	2.3	1	2.5	2.8	1.1	2.8	3.5	1.2	3.1	4.7	1.5	2.7	6	2.2
Dred.:	654	Mean	5.1	7.7	1.5	4.9	7.7	1.6	4.6	8.2	1.8	4.4	8.7	2	4	8.9	2.2
**Cape**	**Lamb.**	Max	51.7	238.9	4.6	31.7	88.4	2.8	10	36.1	3.6	7.4	21.3	2.9	6.7	21.3	3.2
Name:	**MDR**	99th	32.4	64.9	2	18.1	55.3	3.1	9.9	30.1	3	7	20.4	2.9	6.6	18.9	2.9
Distn:	2.1	95th	13.2	27.1	2.1	11.4	28.6	2.5	8.3	23	2.8	6.4	18.9	3	6.4	15.6	2.4
Depth:	3	80th	4.2	7.4	1.8	4.4	8.8	2	5.2	12.1	2.3	4.8	13.2	2.7	4.2	13.3	3.1
Base.:	382	Med.	1.4	2.1	1.5	1.8	2.3	1.3	2.3	3.6	1.5	2.3	5.2	2.3	2.1	6.8	3.2
Dred.:	685	Mean	3.4	6.4	1.9	3.1	6.5	2.1	3.1	7	2.3	2.9	7.2	2.5	2.7	7.4	2.7
**Cape**	**Lamb.**	Max	63.3	142.7	2.3	37.7	77	2	19	37.9	2	13.7	20.9	1.5	10.1	16.4	1.6
Name:	**PLR**	99th	27.1	44.6	1.6	24	40.8	1.7	18.5	27.9	1.5	13.5	20	1.5	10	16.1	1.6
Distn:	9.7	95th	10.6	15.4	1.5	9.7	14.5	1.5	9.5	18.6	2	10.5	16	1.5	9.7	13.7	1.4
Depth:	3	80th	3.8	4	1.1	3.6	4.4	1.2	4.7	5.9	1.2	5.7	5.9	1	6	8.9	1.5
Base.:	399	Med.	1.4	1.3	0.9	1.4	1.4	1	1.7	1.7	1	2.4	2.2	0.9	2.3	3.1	1.4
Dred.:	692	Mean	3	3.8	1.3	2.8	3.9	1.4	3.1	4.1	1.3	3.3	4.3	1.3	3.5	4.7	1.4
**Cape**	**Lamb.**	Max	172.6	220	1.3	54.6	76.2	1.4	17.1	40.3	2.4	14.4	23.5	1.6	11	16.5	1.5
Name:	**PWR**	99th	29.2	74.5	2.6	24.8	59.6	2.4	16.7	37.1	2.2	14.2	23.4	1.6	11	16.5	1.5
Distn:	1.2	95th	15.7	36.1	2.3	14.3	32.4	2.3	11.4	26.8	2.3	12.1	21.3	1.8	10.5	16.3	1.6
Depth:	6	80th	4.8	12.4	2.6	4.5	14.6	3.2	4.6	17.7	3.9	6.8	17.2	2.5	7.2	12.3	1.7
Base.:	399	Med.	1.3	3.3	2.5	1.6	4.1	2.5	2.2	7.1	3.3	2.7	10.3	3.8	4.2	6.7	1.6
Dred.:	629	Mean	3.7	8.9	2.4	3.6	9	2.5	3.4	10.1	3	4.3	10.6	2.5	4.8	8.3	1.7
**Cape**	**Lamb.**	Max	101.5	137.7	1.4	45.4	63.4	1.4	25.8	26.6	1	16.1	18.4	1.1	8.2	16.3	2
Name:	**SMSB**	99th	32.7	46.7	1.4	28.4	40.5	1.4	18.8	24.2	1.3	12.9	17.1	1.3	8	15.8	2
Distn:	5.6	95th	14.2	21.1	1.5	12.6	20.1	1.6	8.1	17.9	2.2	6.4	14.2	2.2	6	12.5	2.1
Depth:	4	80th	4.9	6.7	1.4	4.8	7.4	1.6	4.5	8.2	1.8	4.5	8.3	1.8	3.8	8.2	2.2
Base.:	481	Med.	1.4	2.2	1.5	1.7	2.5	1.5	2.1	3.3	1.6	2.1	4.3	2.1	2.1	4.9	2.3
Dred.:	698	Mean	3.7	5.3	1.4	3.5	5.3	1.5	3.1	5.4	1.7	2.8	5.5	2	2.5	5.5	2.2

Table S1.B. Min, 1st, 5th, 20th percentiles, median and mean DLI values (mol photons/m2) over 1 d, 14 d, 21 d and 30 d (1mth) running average period at all sites during the baseline period ("B", before dredging) or for during the duration of the dredging program ("D"). The ratio of dredging/baseline is also shown ("Δ"). For each site the Distace from dredging activities (Distn), the site depth (Depth) and the number of sampling days during baseline (Base) and dredging (Dred) are shown.

Site	Details	Stat.	B1D	D1D	Δ1D	B1W	D1W	Δ1W	B2W	D2W	Δ2W	B3W	D3W	Δ3W	B4W	D4W	Δ4W
**Barrow**	**Island**	Min	0.1	0	0	0.4	0.2	0.4	0.5	0.7	1.4	0.5	1.4	2.6	0.8	2.1	2.9
Name:	**AHC**	1st	0.3	0.1	0.3	0.5	0.5	0.9	0.5	1.4	2.5	0.6	2.1	3.5	0.8	2.3	2.8
Distn:	32.8	5th	0.6	0.7	1.1	1	1.9	2	1.1	2.3	2	1.2	2.5	2.1	1.3	2.6	2
Depth:	6.9	20th	2.1	2.8	1.3	2.3	3	1.3	2.3	3.1	1.4	2.3	3.2	1.4	2.3	3.2	1.4
Base.:	612	Med.	3.8	5	1.3	3.6	4.8	1.3	3.5	4.7	1.3	3.5	4.7	1.3	3.5	4.8	1.4
Dred.:	548	Mean	3.5	4.8	1.4	3.5	4.8	1.4	3.4	4.9	1.4	3.4	4.9	1.4	3.4	5	1.4
**Barrow**	**Island**	Min	0.5	0.1	0.2	2.2	1.1	0.5	2.8	1.5	0.6	2.8	1.8	0.7	3	2.2	0.7
Name:	**ANT**	1st	1.7	0.6	0.4	2.5	1.6	0.6	2.8	1.7	0.6	2.8	1.9	0.7	3	2.4	0.8
Distn:	8.8	5th	2.4	1.9	0.8	2.9	2.5	0.9	3	2.4	0.8	3	2.7	0.9	3.1	2.7	0.9
Depth:	3.9	20th	4	4.1	1	3.9	4.1	1.1	3.7	4.2	1.1	3.7	4	1.1	3.7	4.1	1.1
Base.:	692	Med.	6.1	6.8	1.1	5.8	6.9	1.2	5.7	7	1.2	5.6	6.9	1.2	5.4	6.9	1.3
Dred.:	388	Mean	6	6.7	1.1	5.8	6.6	1.2	5.6	6.6	1.2	5.4	6.6	1.2	5.2	6.5	1.3
**Barrow**	**Island**	Min	0	0.1	4	1.2	0.9	0.7	1.9	1.2	0.6	2.3	1.8	0.8	2.5	3	1.2
Name:	**BAT**	1st	0.8	0.5	0.6	1.6	1.4	0.9	2.1	1.6	0.8	2.4	2.1	0.9	2.6	3.4	1.3
Distn:	15.5	5th	2	2	1	2.8	2.7	1	2.8	3.3	1.2	2.6	3.8	1.5	2.7	3.9	1.4
Depth:	3.7	20th	4.1	4.7	1.1	4.1	4.8	1.2	4.2	4.9	1.2	4.2	4.7	1.1	4.4	4.6	1.1
Base.:	621	Med.	6.5	8.2	1.3	6.6	8	1.2	6.4	8	1.2	6.5	8	1.2	6.7	8.1	1.2
Dred.:	488	Mean	6.8	8	1.2	6.8	7.9	1.2	6.8	7.9	1.2	6.9	8	1.2	7	8.1	1.2
**Barrow**	**Island**	Min	6.2	0.9	0.1	10.2	3.6	0.3	12.1	4.3	0.4	13.2	4.7	0.4	13.3	5.3	0.4
Name:	**DIW**	1st	8.9	1.5	0.2	10.6	4.4	0.4	12.8	4.8	0.4	13.2	5.1	0.4	14.4	5.6	0.4
Distn:	6.5	5th	11.2	5.2	0.5	12.2	5.9	0.5	13.5	6.2	0.5	13.6	6.5	0.5	14.6	6.2	0.4
Depth:	1.9	20th	13.1	9.1	0.7	13.7	10.2	0.8	14.3	11	0.8	15.1	11	0.7	15.2	11.6	0.8
Base.:	238	Med.	16.1	15	0.9	16.5	14.6	0.9	17	14.9	0.9	17.2	15	0.9	17.3	14.7	0.8
Dred.:	507	Mean	16.7	14.6	0.9	17.1	14.6	0.9	17.5	14.6	0.8	17.7	14.6	0.8	18.1	14.5	0.8
**Barrow**	**Island**	Min	0	0	0	1.3	0.2	0.1	2.3	1	0.5	2.1	1.3	0.6	2.6	1.9	0.7
Name:	**DUG**	1st	0.4	0	0	1.7	0.5	0.3	2.4	1.3	0.5	2.3	1.8	0.8	2.6	1.9	0.7
Distn:	9.2	5th	1.3	0.3	0.2	2.5	1.7	0.7	2.6	2.3	0.9	2.8	2.5	0.9	3	3.2	1.1
Depth:	6	20th	3.4	2.5	0.8	3.6	3	0.8	3.7	3.4	0.9	3.8	3.7	1	3.9	4.2	1.1
Base.:	561	Med.	5.3	5	0.9	6	5.1	0.9	6.2	5.1	0.8	6.1	5.1	0.8	6	5.2	0.9
Dred.:	439	Mean	5.4	4.9	0.9	5.6	5	0.9	5.6	5.1	0.9	5.6	5.2	0.9	5.6	5.4	1
**Barrow**	**Island**	Min	1	0.1	0.1	1.4	1.5	1.1	1.7	2.1	1.3	1.8	2.5	1.4	2	2.6	1.3
Name:	**ELS**	1st	1	0.2	0.2	1.4	1.7	1.2	1.7	2.2	1.3	1.8	2.6	1.4	2	2.7	1.4
Distn:	21	5th	1.4	0.8	0.6	1.5	2.2	1.5	1.7	2.5	1.4	1.9	2.7	1.5	2.1	3	1.4
Depth:	7	20th	2.8	3.1	1.1	2.1	3.2	1.5	2	3.3	1.6	2	3.1	1.6	2.4	3.2	1.3
Base.:	133	Med.	4.8	5	1.1	3.9	4.9	1.3	3.7	5	1.4	3.6	5.3	1.5	3.3	5.2	1.6
Dred.:	411	Mean	4.5	5	1.1	3.8	5	1.3	3.3	5	1.5	3.3	5	1.5	3.3	5	1.5
**Barrow**	**Island**	Min	0.1	0	0	1	0.1	0.1	1.5	0.2	0.2	1.4	0.3	0.2	1.8	0.4	0.2
Name:	**LNG0**	1st	0.3	0	0	1.2	0.2	0.1	1.5	0.3	0.2	1.6	0.3	0.2	1.8	0.4	0.2
Distn:	0.2	5th	1	0	0	1.6	0.3	0.2	1.8	0.4	0.2	1.9	0.4	0.2	1.9	0.4	0.2
Depth:	8.6	20th	1.9	0.3	0.2	2	0.6	0.3	2.1	0.6	0.3	2.2	0.6	0.3	2.2	0.7	0.3
Base.:	476	Med.	2.9	1.1	0.4	2.7	1.2	0.4	2.5	1.3	0.5	2.5	1.3	0.5	2.4	1.3	0.5
Dred.:	482	Mean	3.1	1.5	0.5	3.1	1.5	0.5	3.2	1.6	0.5	3.2	1.5	0.5	3.2	1.5	0.5
**Barrow**	**Island**	Min	0	0	0	0.5	0.1	0.1	0.8	0.2	0.3	0.8	0.3	0.4	1.2	0.4	0.3
Name:	**LNG1**	1st	0.1	0	0	0.7	0.1	0.2	0.9	0.3	0.4	0.9	0.5	0.5	1.2	0.5	0.4
Distn:	0.5	5th	0.7	0.1	0.1	1	0.3	0.3	1	0.4	0.4	1.2	0.6	0.5	1.3	0.7	0.5
Depth:	8.9	20th	1.4	0.4	0.3	1.5	0.7	0.5	1.7	0.8	0.5	1.7	0.9	0.5	1.8	0.9	0.5
Base.:	450	Med.	2.8	1.2	0.4	2.8	1.5	0.5	2.9	1.5	0.5	2.9	1.5	0.5	2.9	1.9	0.6
Dred.:	478	Mean	3.1	1.7	0.5	3.1	1.7	0.5	3.1	1.7	0.5	3.2	1.7	0.5	3.3	1.8	0.5
**Barrow**	**Island**	Min	0	0	0	0.9	0.2	0.3	1.2	0.3	0.3	1.3	0.3	0.3	1.8	0.4	0.2
Name:	**LNG2**	1st	0.2	0	0.2	1.1	0.3	0.3	1.2	0.4	0.3	1.4	0.4	0.3	1.8	0.5	0.3
Distn:	1	5th	0.9	0.2	0.2	1.3	0.4	0.3	1.7	0.5	0.3	1.8	0.5	0.3	1.9	0.5	0.3
Depth:	6.6	20th	2	0.8	0.4	2.1	1.1	0.5	2.1	1.2	0.6	2.5	1.2	0.5	2.7	1.2	0.4
Base.:	636	Med.	3.9	2.2	0.6	4.1	2.3	0.6	4.2	2.4	0.6	4.2	2.5	0.6	4.3	2.6	0.6
Dred.:	442	Mean	4	2.6	0.6	4	2.6	0.6	4.2	2.6	0.6	4.2	2.6	0.6	4.3	2.6	0.6
**Barrow**	**Island**	Min	0	0	0	0.7	0.5	0.6	1.3	0.7	0.5	1.6	1.6	1	1.9	1.9	1
Name:	**LNG3**	1st	0.2	0	0.1	1.1	0.6	0.5	1.7	1.2	0.7	1.9	1.7	0.9	2	1.9	0.9
Distn:	4	5th	0.9	0.4	0.4	1.8	1.1	0.6	2	1.8	0.9	2.1	2	0.9	2.1	2.1	1
Depth:	6.2	20th	2.3	1.6	0.7	2.7	2.1	0.8	2.9	2.3	0.8	3	2.4	0.8	3.4	2.4	0.7
Base.:	628	Med.	4	3.6	0.9	4.5	3.6	0.8	4.5	4.1	0.9	4.9	4.7	1	5.3	4.8	0.9
Dred.:	466	Mean	4.2	3.8	0.9	4.6	3.9	0.9	4.7	4.2	0.9	4.8	4.3	0.9	5	4.5	0.9
**Barrow**	**Island**	Min	0.3	0	0	1.9	0.1	0.1	2.5	0.2	0.1	2.7	0.3	0.1	2.7	0.3	0.1
Name:	**LNGA**	1st	0.4	0	0	1.9	0.2	0.1	2.5	0.2	0.1	2.7	0.3	0.1	2.7	0.3	0.1
Distn:	0.3	5th	1.6	0.1	0	2.1	0.2	0.1	2.6	0.3	0.1	2.7	0.3	0.1	2.8	0.4	0.1
Depth:	11.1	20th	2.4	0.3	0.1	2.6	0.5	0.2	2.8	0.5	0.2	2.8	0.5	0.2	2.8	0.5	0.2
Base.:	113	Med.	2.8	0.8	0.3	2.9	0.9	0.3	2.9	1	0.3	2.9	1.1	0.4	2.9	1.2	0.4

Table S1.B. Min, 1st, 5th, 20th percentiles, median and mean DLI values (mol photons/m2) over 1 d, 14 d, 21 d and 30 d (1mth) running average period at all sites during the baseline period ("B", before dredging) or for during the duration of the dredging program ("D"). The ratio of dredging/baseline is also shown ("Δ"). For each site the Distace from dredging activities (Distn), the site depth (Depth) and the number of sampling days during baseline (Base) and dredging (Dred) are shown.

Site	Details	Stat.	B1D	D1D	Δ1D	B1W	D1W	Δ1W	B2W	D2W	Δ2W	B3W	D3W	Δ3W	B4W	D4W	Δ4W
Dred.:	468	Mean	2.8	1	0.4	2.9	1	0.4	2.9	1.1	0.4	2.9	1.1	0.4	2.9	1.2	0.4
**Barrow**	**Island**	Min	0.5	0	0	1.4	0.1	0.1	2.2	0.2	0.1	2.4	0.3	0.1	2.6	0.4	0.1
Name:	**LNGB**	1st	0.6	0	0	1.4	0.1	0.1	2.2	0.3	0.1	2.4	0.4	0.2	2.6	0.5	0.2
Distn:	0.7	5th	1	0.1	0.1	2	0.3	0.2	2.4	0.5	0.2	2.5	0.6	0.2	2.7	0.6	0.2
Depth:	10.2	20th	2.4	0.4	0.2	2.7	0.7	0.2	2.8	0.8	0.3	2.8	0.8	0.3	2.9	0.8	0.3
Base.:	115	Med.	3	1.2	0.4	3	1.3	0.4	3	1.3	0.4	3.1	1.5	0.5	3.2	2	0.6
Dred.:	481	Mean	3.1	1.6	0.5	3.4	1.6	0.5	3.4	1.7	0.5	3.4	1.7	0.5	3.4	1.8	0.5
**Barrow**	**Island**	Min	0.1	0	0	0.7	0.1	0.1	1.6	0.3	0.2	1.5	0.4	0.3	2	0.4	0.2
Name:	**LNGC**	1st	0.5	0	0	1	0.1	0.1	1.6	0.3	0.2	1.6	0.5	0.3	2	0.5	0.2
Distn:	1.4	5th	1	0	0	1.5	0.2	0.2	1.7	0.5	0.3	2	0.5	0.3	2.2	0.6	0.3
Depth:	10.7	20th	2.5	0.3	0.1	2.5	0.5	0.2	2.6	0.7	0.3	2.6	0.6	0.2	2.8	0.7	0.2
Base.:	241	Med.	3.3	1	0.3	3.4	1.1	0.3	3.5	1	0.3	3.6	1.1	0.3	3.7	1	0.3
Dred.:	424	Mean	3.1	1.2	0.4	3.2	1.3	0.4	3.2	1.3	0.4	3.3	1.3	0.4	3.4	1.3	0.4
**Barrow**	**Island**	Min	2.1	-	-	5.4	-	-	-	-	-	-	-	-	-	-	-
Name:	**LOW**	1st	2.2	-	-	5.4	-	-	-	-	-	-	-	-	-	-	-
Distn:	1.9	5th	4.2	-	-	5.4	-	-	-	-	-	-	-	-	-	-	-
Depth:	2.9	20th	6.8	-	-	5.4	-	-	-	-	-	-	-	-	-	-	-
Base.:	75	Med.	11.6	-	-	9	-	-	-	-	-	-	-	-	-	-	-
Dred.:	0	Mean	10.3	-	-	9	-	-	-	-	-	-	-	-	-	-	-
**Barrow**	**Island**	Min	0.7	0	0	2.4	0.4	0.2	3.5	0.5	0.2	3.6	0.9	0.2	3.8	1.1	0.3
Name:	**LOW1**	1st	1	0.1	0.1	3	0.6	0.2	3.5	0.8	0.2	3.7	0.9	0.2	3.8	1.2	0.3
Distn:	1.6	5th	2.6	0.9	0.3	3.2	1.5	0.5	3.6	1.6	0.4	3.8	1.6	0.4	3.9	1.5	0.4
Depth:	6.9	20th	3.6	2.3	0.7	3.7	2.5	0.7	3.9	2.5	0.6	4.1	2.4	0.6	4.1	2.3	0.6
Base.:	173	Med.	4.3	3.9	0.9	4.3	3.8	0.9	4.3	3.9	0.9	4.3	3.9	0.9	4.3	3.7	0.9
Dred.:	524	Mean	4.2	4	1	4.2	3.9	0.9	4.3	3.8	0.9	4.3	3.7	0.9	4.3	3.6	0.8
**Barrow**	**Island**	Min	6.3	0	0	9.3	1.1	0.1	-	1.2	-	-	1.9	-	-	2.3	-
Name:	**LOW3**	1st	6.5	0.4	0.1	9.3	1.3	0.1	-	1.5	-	-	1.9	-	-	2.5	-
Distn:	2.2	5th	7.1	1.5	0.2	9.4	2.4	0.3	-	2.5	-	-	2.8	-	-	3.2	-
Depth:	4.5	20th	8.6	4.2	0.5	9.8	4.6	0.5	-	4.9	-	-	4.7	-	-	4.6	-
Base.:	10	Med.	10.4	7.7	0.7	10.3	7.4	0.7	-	7.2	-	-	7.1	-	-	7	-
Dred.:	447	Mean	9.7	7.5	0.8	10.1	7.5	0.7	-	7.4	-	-	7.3	-	-	7.1	-
**Barrow**	**Island**	Min	0.5	0	0	0.9	0.1	0.2	1	0.4	0.4	1	0.5	0.5	1.4	0.6	0.4
Name:	**MOF1**	1st	0.7	0	0.1	1	0.3	0.3	1	0.5	0.4	1	0.5	0.5	1.6	0.6	0.4
Distn:	0.8	5th	1.1	0.2	0.2	1.3	0.6	0.4	1.8	0.6	0.4	1.9	0.6	0.3	2.1	0.7	0.3
Depth:	6.2	20th	2.1	1	0.5	2.3	1.2	0.5	2.4	1.5	0.6	2.5	1.6	0.6	2.9	1.7	0.6
Base.:	562	Med.	3.6	2.2	0.6	3.6	2.2	0.6	3.6	2.3	0.6	3.6	2.4	0.6	3.5	2.5	0.7
Dred.:	512	Mean	3.6	2.2	0.6	3.6	2.2	0.6	3.6	2.2	0.6	3.7	2.3	0.6	3.7	2.3	0.6
**Barrow**	**Island**	Min	0.4	0	0	1.9	0.3	0.1	2.2	0.4	0.2	2.3	0.7	0.3	2.4	0.9	0.4
Name:	**MOF3**	1st	0.7	0.1	0.1	2.1	0.5	0.3	2.3	0.6	0.3	2.5	0.8	0.3	2.5	1	0.4
Distn:	1.5	5th	1.4	0.5	0.4	2.4	0.9	0.4	2.5	1	0.4	2.6	1.2	0.5	2.7	1.1	0.4
Depth:	4.8	20th	3	1.8	0.6	3.2	2.1	0.6	3.2	2.5	0.8	3.1	2.7	0.9	3.2	2.8	0.9
Base.:	549	Med.	4.9	3.8	0.8	5.1	3.7	0.7	5.2	3.9	0.8	5	4.1	0.8	5.1	4.5	0.9
Dred.:	487	Mean	4.9	3.9	0.8	5	3.9	0.8	5	4.1	0.8	5	4.2	0.8	4.9	4.4	0.9
**Barrow**	**Island**	Min	0	0	0	0.6	0	0	0.9	0.1	0.1	1.2	0.5	0.4	1.5	0.8	0.5
Name:	**MOFA**	1st	0	0	0.1	0.7	0.2	0.3	0.9	0.3	0.3	1.3	0.5	0.4	1.5	0.8	0.6
Distn:	0.6	5th	0.2	0.1	0.7	1.2	0.6	0.5	1.2	1	0.8	1.5	1.2	0.8	1.7	1.3	0.7
Depth:	4.9	20th	1.7	1.1	0.7	2	1.8	0.9	2.1	2	1	2	2.3	1.1	2	2.5	1.3
Base.:	137	Med.	2.8	2.9	1.1	2.6	2.9	1.1	2.4	3.2	1.3	2.5	3.3	1.3	2.6	3.3	1.3
Dred.:	456	Mean	3.5	3.1	0.9	3.4	3.2	0.9	3.1	3.3	1.1	3	3.4	1.1	3	3.4	1.2
**Barrow**	**Island**	Min	0.9	0	0	1.2	0.2	0.2	1.3	0.3	0.2	1.7	0.4	0.2	2.2	0.5	0.2
Name:	**MOFB**	1st	1.1	0.1	0.1	1.2	0.3	0.3	1.4	0.4	0.3	1.9	0.4	0.2	2.3	0.5	0.2
Distn:	1	5th	1.4	0.2	0.2	1.5	0.4	0.3	2.2	0.5	0.2	2.6	0.6	0.2	2.8	0.6	0.2
Depth:	7.5	20th	3.2	0.9	0.3	3.4	1.1	0.3	3.6	1.1	0.3	3.7	1	0.3	3.8	0.9	0.2
Base.:	211	Med.	4	2.3	0.6	4.1	2.4	0.6	4	2.4	0.6	4	2.4	0.6	4.1	2.4	0.6
Dred.:	488	Mean	3.8	2.2	0.6	3.9	2.2	0.6	3.9	2.2	0.6	3.9	2.2	0.6	4	2.2	0.5
**Barrow**	**Island**	Min	0.8	0	0	1.2	0	0	1.4	0.2	0.1	1.6	0.2	0.1	2	0.4	0.2
Name:	**MOFC**	1st	1	0	0	1.2	0.2	0.1	1.4	0.2	0.1	1.6	0.2	0.2	2	0.5	0.3
Distn:	0.8	5th	1.3	0.1	0.1	1.4	0.3	0.2	1.5	0.4	0.3	1.7	0.5	0.3	2.1	0.7	0.3
Depth:	6.9	20th	2.6	0.8	0.3	3	1.1	0.4	3.2	1.2	0.4	3.2	1.7	0.5	3.2	1.7	0.5
Base.:	154	Med.	3.9	2.3	0.6	3.9	2.4	0.6	4.1	2.6	0.6	4	2.7	0.7	4.2	2.6	0.6
Dred.:	471	Mean	3.8	2.2	0.6	3.9	2.3	0.6	3.9	2.4	0.6	3.9	2.4	0.6	4	2.4	0.6
**Barrow**	**Island**	Min	0.1	0	0	1.5	0.3	0.2	1.9	0.5	0.3	2.2	1.2	0.5	2.3	1.8	0.8
Name:	**REFN**	1st	0.1	0.1	1.1	1.6	0.4	0.3	1.9	0.8	0.4	2.2	1.2	0.6	2.3	3	1.3
Distn:	28	5th	1.1	0.8	0.7	1.7	2.1	1.2	2	2.3	1.1	2.2	2.7	1.2	2.3	3.3	1.4
Depth:	7.2	20th	2.2	3.4	1.6	2.3	3.5	1.6	2.2	3.8	1.7	2.3	3.9	1.7	2.4	4.2	1.8

Table S1.B. Min, 1st, 5th, 20th percentiles, median and mean DLI values (mol photons/m2) over 1 d, 14 d, 21 d and 30 d (1mth) running average period at all sites during the baseline period ("B", before dredging) or for during the duration of the dredging program ("D"). The ratio of dredging/baseline is also shown ("Δ"). For each site the Distace from dredging activities (Distn), the site depth (Depth) and the number of sampling days during baseline (Base) and dredging (Dred) are shown.

**Site**	**Details**	**Stat.**	**B1D**	**D1D**	**Δ1D**	**B1W**	**D1W**	**Δ1W**	**B2W**	**D2W**	**Δ2W**	**B3W**	**D3W**	**Δ3W**	**B4W**	**D4W**	**Δ4W**
Base.:	93	Med.	3.3	5.9	1.8	3.2	6.1	1.9	2.6	6.1	2.4	2.4	5.9	2.5	2.5	6	2.4
Dred.:	426	Mean	3.4	5.3	1.6	3.2	5.4	1.7	2.9	5.5	1.9	2.8	5.6	2	2.6	5.8	2.2
**Barrow**	**Island**	Min	1.2	0	0	3.4	0.4	0.1	4.4	0.7	0.2	4.8	1.4	0.3	5.4	1.9	0.3
Name:	**REFS**	1st	1.6	0.1	0	3.7	0.8	0.2	4.6	0.9	0.2	4.9	1.5	0.3	5.4	2	0.4
Distn:	23.6	5th	3.8	1.3	0.4	4.4	2.6	0.6	4.8	2.3	0.5	5.3	2.4	0.5	5.8	2.5	0.4
Depth:	5	20th	5.6	4.4	0.8	5.8	4.7	0.8	5.9	4.7	0.8	6.1	4.8	0.8	6.3	4.8	0.8
Base.:	144	Med.	7	7.2	1	6.8	7.2	1.1	6.7	7.2	1.1	6.7	7.5	1.1	6.7	7.6	1.1
Dred.:	429	Mean	7.1	7	1	6.9	7	1	6.6	7.1	1.1	6.6	7.1	1.1	6.6	7.2	1.1
**Barrow**	**Island**	Min	0.1	0	0.4	0.4	0.7	1.7	0.5	1.6	3.5	0.7	2.5	3.6	0.8	3	3.7
Name:	**SBS**	1st	0.2	0.1	0.5	0.5	0.9	1.9	0.7	2.1	3.2	0.8	3.2	4.2	0.9	3.3	3.5
Distn:	29.9	5th	0.7	1.4	1.9	1.1	2.6	2.5	1.3	3.2	2.4	1.3	3.6	2.9	1.4	3.9	2.8
Depth:	4.7	20th	2.9	4.6	1.6	3.1	5.1	1.7	3.4	5.3	1.5	3.7	5.2	1.4	3.9	5.5	1.4
Base.:	605	Med.	5.9	7.2	1.2	5.7	6.9	1.2	5.8	7.2	1.2	5.9	7.5	1.3	5.6	7.8	1.4
Dred.:	502	Mean	6	7	1.2	5.9	7.1	1.2	5.8	7.2	1.2	5.6	7.3	1.3	5.6	7.4	1.3
**Barrow**	**Island**	Min	0.9	0	0	1.7	0.1	0	1.8	0.2	0.1	1.9	0.6	0.3	2.8	1	0.4
Name:	**TR**	1st	1.5	0.1	0	1.7	0.3	0.2	1.9	0.4	0.2	1.9	0.8	0.4	3.1	1.2	0.4
Distn:	5	5th	2	0.3	0.1	2.1	1.2	0.6	2.1	1.4	0.6	3.3	1.6	0.5	4.3	1.6	0.4
Depth:	4.5	20th	5.6	2.4	0.4	5.1	3.4	0.7	5.4	3	0.6	5.7	2.6	0.5	6.3	2.4	0.4
Base.:	241	Med.	7.8	5.6	0.7	7.9	5.4	0.7	8	5.6	0.7	8.1	5.5	0.7	8.6	5.3	0.6
Dred.:	464	Mean	7.7	5.7	0.7	7.8	5.6	0.7	8	5.5	0.7	8.2	5.4	0.7	8.5	5.2	0.6
